# Thermally and Light-Induced Spin-Crossover in Iron(III) Complexes with Benzophenone-Based Saltrien Ligands: Hysteresis, Two-Step Transitions, and the LIESST Effect

**DOI:** 10.1021/acs.inorgchem.5c04707

**Published:** 2025-12-29

**Authors:** Lukáš Pogány, Kamil Kotrle, Ivan Nemec, Ján Moncol, Milan Mazúr, Ivan Šalitroš

**Affiliations:** † Department of InorganicChemistry, Faculty of Chemical and Food Technology, Slovak University of Technology in Bratislava, Bratislava SK-81237, Slovakia; ‡ Department of Inorganic Chemistry, Facultyof Science, Palacký University, 17. listopadu 12, Olomouc 771 46, Czech Republic; § Department of Physical Chemistry, Facultyof Chemical and Food Technology, SlovakUniversity of Technology in Bratislava, Bratislava SK-81237, Slovakia; # Central European Institute of Technology, Brno University of Technology, Purkyňova 123, Brno 61200, Czech Republic

## Abstract

The synthesis and characterization of four new iron­(III) coordination compounds with *saltrien*-like hexadentate Schiff base ligands L_*n*_, prepared by condensation between triethylenetetramine and 2-hydroxy-3,5-dimethylbenzophenone (L_1_, **C1**–**C3**) or 2-hydroxy-5-methoxybenzophenone (L_2_, **C4**), are reported. The complexes [Fe­(L_*n*_)]­X·mCH_3_CN (X = SeCN^–^ for **C1** and **C4**, SCN^–^ for **C2** and BPh_4_^–^ for **C3**, *m* = 0 for **C3**, *m* = 1 for **C1** and **C4**, *m* = 2 for **C2**) were structurally characterized, and their spin-crossover (SCO) was monitored by magnetic measurements, X-ray powder diffraction analysis, and EPR spectroscopy. Intermolecular interactions relevant to SCO were analyzed through Hirshfeld surface maps and QT-AIM calculations. All compounds exhibit SCO above room temperature in their solvated forms, and ab initio calculations were employed to probe their electronic structures. While the computed ^2^ *T*_2g_–^6^ *A*_1g_ gaps and 10Dq energies are consistent across the whole series, the experimental *T*_1/2_ values do not directly reflect these energy differences. Instead, SCO is predominantly controlled by crystal packing effects, including intermolecular connectivity, internal pressure, lattice rigidity, and solvation. Upon heating, solvent removal in **C1** and **C2** shifts their SCO to below room-temperature. The desolvated compounds **C1d** and **C2d** exhibit sharp SCO with wide hysteresis, while **C2d** additionally features a second gradual step (**C1d**: *T*_1/2_ = 82 K/166 K; **C2d**: *T*(1)_1/2_ = 170 K/153 K, *T*(2)_1/2_ = 110 K). Furthermore, both compounds are LIESST active upon blue light irradiation (*T*(LIESST)=57 K for **C1d** and 36 K for **C2d**). These results underscore the crucial role of ligand flexibility, solvation, and intermolecular interactions on SCO and highlight the potential of these iron­(III) complexes in molecular switching applications.

## Graphical abstract

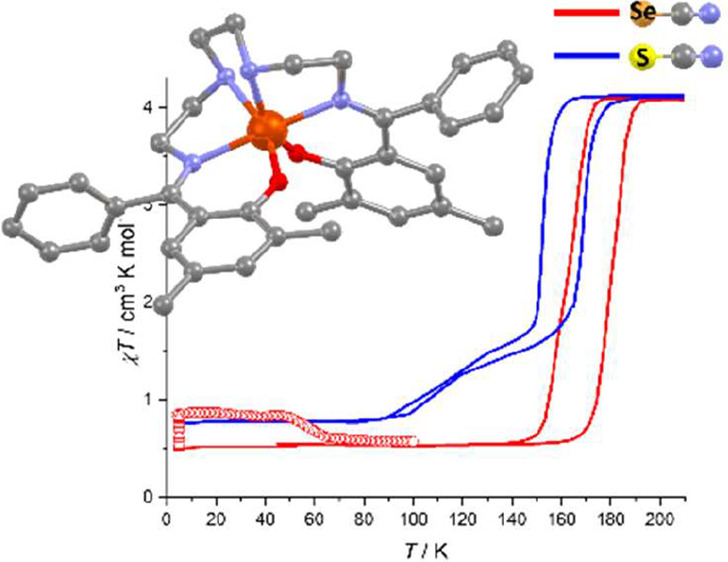

## Introduction

First-row transition metal complexes with a 3*d* ^*n*^ (*n* = 4–7) electronic configuration and pseudo-octahedral symmetry of the central ion can exhibit spin-crossover (SCO) effect. These complexes function as molecular switches exhibiting reversible and reproducible transitions between high-spin (HS) and low-spin (LS) states under external stimuli such as temperature, pressure, light, and magnetic fields.^1^ The SCO behavior can be further finely tuned through various chemical modifications, including ligand substitution,^2^ variation of cocrystallized solvents,^3^ and the choice of counter-anions.^4^ Remarkably, the SCO switching is retained even at the single-molecule level, making SCO complexes highly promising for next-generation technologies, particularly in areas such as spin-based data processing^5^ and high-density data storage.^6^ Moreover, the HS–LS transition in SCO materials is often accompanied by pronounced changes in properties like color,^7^ fluorescence,^8^ electrical resistance,^9^ dielectric constant,^10^ and mechanical behavior,^11^ making them highly versatile for applications in optical and electronic devices.

In addition to the extensively studied iron­(II) SCO complexes, iron­(III) SCO complexes have also attracted significant interest,^12^ primarily due to their enhanced air stability, which makes them promising for practical applications. A particularly well-studied class of ligands that promote SCO behavior in iron­(III) complexes is hexadentate N_4_O_2_-donor derivatives of *saltrien* (2,2′-((1,11)-2,5,8,11-tetraazadodeca-1,11-diene-1,12-diyl)­diphenol).^13^ These Schiff base ligands offer an excellent platform for systematic investigations of thermal SCO, owing to their cost-effective synthesis, structural versatility, and the ability to fine-tune SCO properties through targeted ligand substitutions.^13a^ The literature reports numerous iron­(III) complexes with the unsubstituted *saltrien* ligand, in which the transition temperature and cooperativity have been systematically tuned through counteranion variation, ranging from simple inorganic anions,^14^ through radical or complex anions,^15^ to oxalate- and anilate-based polymeric anions.^16^ The choice of counteranion influences internal lattice pressure and modulates intermolecular interactions, both of which significantly affect the magnetic behavior of the iron­(III)–*saltrien* cation. As a result, these factors determine whether the complex adopts permanent HS behavior or displays thermal SCO. In parallel, the systematic functionalization of the *saltrien* ligand skeleton has also proven to be an effective strategy for tuning magnetic properties. Most studies have focused on introducing halide,^14c,17^ hydroxyl, and alkoxy^18^ substituents on the phenyl moieties. In addition, some reports describe iron­(III)–*saltrien* complexes bearing long *n*-alkyl chains on the triethylenetetramine backbone^14d,19^ or incorporating naphthalene rings^14c,20^ in place of the phenyl groups. The majority of to date reported iron­(III)–*saltrien* compounds with molecular structures are reviewed in Table S1 (see the ESI). This overview reveals that the occurrence of thermal SCO within this family of complexes is rather unpredictable, as all types of behavior are represented across different structural variants, including permanently HS compounds, those exhibiting SCO below room temperature, and others showing SCO above room temperature. Among these, several noteworthy examples exhibit desirable features such as abrupt^15a,17c,18d,18f,20^ and sometimes hysteretic^18b,18f^ SCO behavior. Despite these advances, drawing definitive conclusions regarding the influence of counteranions and/or *saltrien* substituents on SCO behavior remains highly challenging.

Halcrow and co-workers attributed the miscellaneous magnetic behavior of iron­(III)-*saltrien* analogues to the flexibility of the molecular structure, particularly the conformation of the coordinated ligand.^14c,21^ One of the key structural parameters influencing this behavior is the angle α formed between the least-squares planes of the phenolate rings (Figure 1). In general, with a few exceptions, permanently HS compounds tend to adopt ligand conformations where the α angle is greater than 100°, while those that remain LS up to room temperature typically exhibit α angles in the range of approximately 60°–80°. Additionally, intermolecular interactions in the crystal lattice promote cooperative effects, accounting for the abrupt spin transitions and, in some cases, thermal hysteresis.

**Figure 1:**
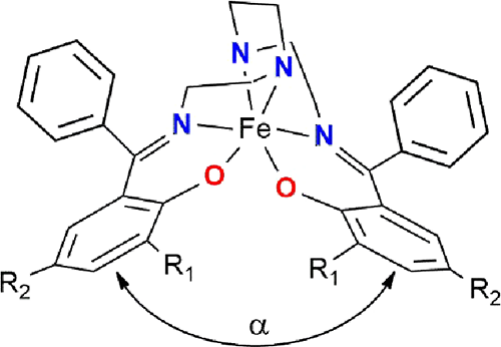
Structure of the [Fe­(L_n_)]^+^ complex cation (R_1_ = R_2_ = CH_3_ for **C1**-**C3**; R_1_ = H, R_2_ = OCH_3_ for **C4**).

Herein, we report on the synthesis and comprehensive characterization focused on the thermal and photoinduced SCO in a series of iron­(III) compounds featuring hexadentate *saltrien*-like ligands prepared from triethylenetetramine and derivatives of 2-hydroxybenzophenone. Four compounds with the general formula [Fe­(L_*n*_)]­X·*m*CH_3_CN (Figure 1) were characterized structurally, spectroscopically, and magnetically, with their SCO properties further elucidated by ab initio calculations. Furthermore, the influence of intermolecular interactions on SCO behavior was investigated by using Hirshfeld surface analysis and quantum theory of atoms in molecules (QT-AIM) approaches. All compounds exhibit SCO values above room temperature. In some cases, removing lattice solvents activated a sharp, hysteretic, and two-step SCO below room temperature, as evidenced by variable-temperature magnetic susceptibility measurements, powder X-ray diffraction, and EPR spectroscopy. Additionally, the solvent-free derivatives demonstrated LIESST activity at cryogenic temperatures.

## Experimental Part

### Materials and Physical Measurements

All purchased compounds (FeCl_3_.6H_2_O, KSeCN, KSCN, Et_3_N, and NaBPh_4_) and solvents (acetonitrile p.a., methanol p.a., and diethyl ether p.a.) were used as received without any further purification. Starting reagents 2-hydroxy-3,5-dimethylbenzophenone^22^ and 2-hydroxy-5-methoxybenzophenone^23^ were prepared according to the reported procedure. All solvents and purchased chemicals were used as received without further purification. The infrared spectra (4000–400 cm^–1^, ATR technique) were obtained at room temperature on a Magna FT-IR 750, Nicolet spectrophotometer. The electronic spectra (190–1100 nm) were recorded in nujol suspension at room temperature on a Specord 250 plus Analytical Jena spectrophotometer. Elemental analysis was performed by using an Eager 300 (Carlo Erba) elemental analyzer. Thermogravimetric analysis was carried out using a simultaneous TG/DTA analyzer SII EXSTAR 6300­(Seiko Instruments, Japan). Each sample (∼5 mg) was heated up in a nitrogen purge at a heating rate of 5 °C/min from 40 °C until a significant mass loss due to decomposition was observed.

### General Procedure for Preparation of Complexes

The Schiff base ligands H_2_L_*n*_ (*n* = 1–2) were obtained by condensation of 2-hydroxy-3,5-dimethylbenzophenone (4.0 mmol, 2 eq., for H_2_L_1_ and **C1**–**C3**) or 2-hydroxy-5-methoxybenzophenone (4.0 mmol, 2 eq., for H_2_L_1_ and **C4**) with triethylenetetramine (2.0 mmol, 1 equiv) in a methanol:acetonitrile mixture (1:1 v/v, 25 mL). The reaction mixture was refluxed for 45 min at 70 °C (Scheme S1, see the Supporting Information (ESI)). Prepared Schiff base ligands were subsequently used without isolation, purification, and characterization. In the next step, the solution of the corresponding ligand was combined with anhydrous iron­(III) chloride (0.324 g, 2.0 mmol, 1 equiv) dissolved in a methanol:acetonitrile mixture (1:1 v/v, 25 mL), followed by the addition of triethylamine (0.405 g, 4 mmol, 2 equiv). The solution of the in situ prepared [Fe­(L_n_)]­Cl precursor complex was refluxed for 15 min. Finally, solid sodium or potassium salt of corresponding counteranion (4.6 mmol, 2.3 eq., KSeCN for **C1** and **C4**, KSCN for **C2**, and NaBPh_4_ for **C3**) was added, and the reaction mixture was refluxed for another 45 min at 70 °C (Scheme S1). The resulting solution was filtered, and single crystals of complexes described by the general formula [Fe­(L_*n*_)]­X·*m*CH_3_CN suitable for X-ray diffraction analysis were collected after a few days of controlled evaporation at laboratory temperature.

**C1** ([Fe­(L_1_)]­SeCN·CH_3_CN): Complex **C1** was prepared according to the general procedure, starting from 2-hydroxy-3,5-methylbenzophenone (0.905 g, 4.0 mmol, 2 equiv). Potassium selenocyanate (0.648 g, 4.6 mmol, 2.3 equiv) was used in the last step of the synthesis. Yield 51%. FT–IR (ATR, *ṽ*_max_/cm^–1^): 3102 (w, N–H), 3057 (w, C_ar_–H),2912 (w, C_al_–H), 2866 (w, C–H_al_), 2063 (s, NCSe), 1573 (sh, C=N). UV–vis (nujol, λ_max_/nm): 531, 458, 357, 262. Elemental analysis for C_37_H_40_FeN_5_O_2_Se·CH_3_CN (755.01 g mol^–1^): Found % (Calc. %): C 61.78 (61.42), H 5.78 (5.68), N 10.88 (11.02).

**C2** ([Fe­(L_1_)]­SCN·2CH_3_CN): Complex **C2** was prepared according to the general procedure, starting from 2-hydroxy-3,5-methylbenzophenone (0.905 g, 4.0 mmol, 2 equiv). Potassium thiocyanate (0.437 g, 4.6 mmol, 2.3 equiv) was used in the last step of the synthesis. Yield 45%. FT–IR (ATR, *ṽ*_max_/cm^–1^): 3105 (w, N–H), 2932 (w, C_al_–H), 2899 (w, C_al_–H), 2061 (s, NCS), 1575 (sh, C=N). UV–vis (nujol, λ_max_/nm): 513, 460, 356, 251. Elemental analysis for C_37_H_40_FeN_5_O_2_S·2CH_3_CN (756.76 g·mol^–1^): Found % (Calc. %): C 64.78 (65.07); H, 5.95 (6.13); N, 13.01 (12.96).

**C3** ([Fe­(L_1_)]­BPh_4_): Complex **C3** was prepared according to the general procedure, starting from 2-hydroxy-3,5-methylbenzophenone (0.905 g, 4.0 mmol, 2 equiv). Sodium tetraphenylborate (1.54 g, 4.6 mmol, 2.3 equiv) was used in the last step of the synthesis. Yield 69%. FT–IR (ATR, *ṽ*_max_/cm^–1^): 3224 (w, N–H), 3055 (w, C–H_ar_),2998 (w, C_al_–H), 2912 (w, C_al_–H), 1577 (sh, C=N). UV–vis (nujol, λ_max_/nm): 632, 388, 245. Elemental analysis for C_60_H_60_BFeN_4_O_2_ (935.78 g mol^–1^): Found % (Calc. %): C 76.78 (77.01); H, 6.76 (6.46); N, 6.00 (5.99).

**C4** ([Fe­(L_2_)]­SeCN·CH_3_CN): Complex **C4** was prepared according to the general procedure, starting from 2-hydroxy-5-methoxybenzophenone (0.913 g, 4.0 mmol, 2 equiv). Potassium selenocyanate (0.648 g, 4.6 mmol, 2.3 equiv) was used in the last step of the synthesis. Yield 41%. FT–IR (ATR, *ṽ*_max_/cm^–1^): 3109 (w, N–H), 2928 (w, C–H_al_), 2903 (w, C_al_–H), 2056 (s, NCSe), 1585 (sh, C=N). UV–vis (nujol, λ_max_/nm): 629, 387, and 256. Elemental analysis for C_35_H_36_FeN_5_O_4_Se·CH_3_CN (766.55 g mol^–1^): Found % (Calc. %): C 67.87 (57.97); H, 5.05 (5.13); N, 10.52 (10.96).

### Diffraction Studies

#### SCXRD

Data collection and cell refinement of **C1–** **C4** were made by Stoe StadiVari diffractometer using Pilatus3R 300 K HPAD detector and microfocus source Xenocs Genix3D Cu HF (CuKα radiation λ = 1.54186 Å). The structures were solved by direct or charge-flipping methods using SHELXT^24^ or SUPERFLIP,^25^ and refined by the full-matrix least-squares procedure with SHELXL (version 2018/3).^26^ Geometrical analyses were performed with SHELXL. The structures were drawn using the MERCURY.^27^ Crystal data and conditions of data collection and refinement are listed in Table S1.

The data for **C1d** were collected on a Rigaku XtaLAB Synergy-I diffractometer (Rigaku Corporation, Tokyo, Japan), equipped with a HyPix3000 hybrid pixel array detector and a microfocused PhotonJet-I X-ray source (Cu Kα radiation), at 190.0(2) K. Data integration, scaling, and absorption correction were performed using the CrysAlisPro software (version 1.171.40.82a).^28^ The crystal structures were solved using SHELXT, and all non-hydrogen atoms were refined anisotropically on F^2^ using the full-matrix least-squares method implemented in Olex2.refine within the OLEX2 suite (version 1.5).^27^ Hydrogen atoms were located in difference Fourier maps and refined using a riding model with *U*_iso_(H) set to 1.2 *U*_eq_ for −CH_2_ groups and 1.5 *U*_eq_ for −CH_3_ groups. Nonroutine aspects of refinement: the quality of the desolvated single crystal of **C1d** was very poor, resulting in a weak data set and a model with relatively high Rint and R1 values.

#### PXRD

Variable-temperature powder diffraction experiments for **C1** and **C2** were conducted using a Rigaku XtaLAB Synergy-I diffractometer, the same instrument used for SC-XRD measurements. In both cases, the temperature was controlled with a Cryostream 800 unit, and measurements were taken after the temperature had stabilized. The samples were prepared as powder emulsions dispersed in high-viscosity oil with a consistency suitable for placement into a nylon loop. The composition of the emulsions was optimized to achieve the best possible resolution of the diffraction peaks. The powder samples of **C3** and **C4** were ground and placed into the 0.5 mm borosilicate glass capillary. The diffraction data were collected using a PANalytical Empyrean powder diffractometer in transmission mode with CuKα_1,2_ radiation focused by the incident focusing mirror.

#### Hirshfeld Surface Analysis

The software CrystalExplorer (ver. 21.5)^29^ was used to calculate Hirshfeld surfaces^30^ and associated fingerprint plots.^31^ The Hirshfeld surfaces have been calculated by including all orientations of the disordered molecules with their partial occupancies.

### Magnetic Measurements

Magnetic measurements of herein reported coordination compounds were performed on an MPMS-XL-7 SQUID magnetometer, Quantum Design Inc. The temperature dependency of magnetization was recorded at *B* = 0.1 T as an external magnetic field, and the sweeping rate of 1 K min^–1^ was the same for cooling and heating modes. Gelatin capsules were used as sample containers for the measurement in the temperature range of 5 ↔ 400 K. Desolvation of **C1** and **C2**–**C1d** and C**2d**, respectively, was obtained in situ within the magnetic measurements. After the first heating, three continuous cooling/heating cycles were applied until the last two measurements were identical. Thereby, the sample was maintained in the MPMS magnetometer at 380 K for 20 min before every cooling/heating cycle. The very small diamagnetic contribution of the gelatin capsule and high-temperature sample holder had a negligible contribution to the overall magnetization. The diamagnetic corrections of the molar magnetic susceptibilities were applied using Pascal’s constants.^32^

For LIESST experiments, samples **C1d** and **C2d** were prepared using a specialized method developed to enable the photomagnetic investigation of larger powder quantities. In this method, a precisely weighed amount (≈1 mg) of finely ground **C1d** or **C2d** powder was mixed with a weighed quantity of melted eicosane. This mixture was deposited onto the bottom end of a transparent straw, standardly used for magnetic measurements, and left to congeal into a thin solid disk. The bottom of this straw was then connected to a second straw and secured with transparent tape, forming an extended sample holder. Next, the total length of prepared straw was adjusted to match the standard holder used in the MPMS magnetometer, with the sample disk carefully positioned at its midpoint. Prior to magnetic characterization, each prepared sample was irradiated outside of the magnetometer to verify its optical transparency. The exact weight of samples was obtained by weighing and verified by comparison of the thermal χT vs *T* curve with a more accurately weighed sample of the same compound. After cooling to 5 K, the sample was irradiated, and the change in magnetization followed. The irradiation was performed by using a diode-pumped solid-state laser (DPSS) Kvant with three different wavelengths (λ = 637 nm, 300 mW; λ = 532 nm, 300 mW; λ = 405 nm, 150 mW) in order to test the highest yield of LS-to-HS phototransformation. Laser output irradiation was coupled through an optical fiber to the cavity of a MPMS SQUID XL7, and the power on the sample surface was adjusted to 10 mW cm^–2^. In the case of desolvated compounds **C1d** and **C2d**, the most intense increase of magnetic moment was observed under blue-light irradiation (405 nm). When the saturation point had been reached, the light was switched off, the temperature was increased at a rate of 0.3 K min^–1^, and the magnetization was measured at 1 K intervals. T­(LIESST) value was determined from the minimum of the ∂(χT)/∂*T* vs *T* curve for the relaxation process.

### Computational Studies

All theoretical calculations were performed with the use of ORCA 5.0.2. program package.^33^ Initial structures, obtained from X-ray, were treated by DFT hydrogen optimization, with BP86 functional,^34^ with basis sets from Ahlrich def2 basis set,^35^ TZVPP basis for Fe, and TZVP for other atoms, and def2/J auxiliary basis. CASSCF calculations were done with the TZVPP basis for Fe and the TZVP basis for all other atoms and with def2/J and def2-TZVP/C auxiliary basis sets. Dynamic correlation was treated by the RI-NEVPT2 method.^36^ CASSCF was performed for 5 electrons in 5 *d*-orbitals (selected by the ORCA keyword “actorbs dorbs”), which responds to the Fe­(III) valence electron sphere. The number of calculated roots responds to the maximal number of possible roots: 1 state with multiplicity *M*_S_ = 6, 24 roots with *M*_S_ = 4, and 75 roots with *M*_S_ = 2. CASSCF calculations were done with the “NoFrozenCore” keyword. DFT optimizations of LS/HS states were done on structures optimized for the corresponding spin state, with OPBE functional^37^ and CPCM^38^ explicit field for acetonitrile, to compensate effect of the crystal environment. Structures were optimized with frequency calculations and checked to have no imaginary frequencies. Calculations were done with the same basis set as that mentioned above on hydrogen optimization. All calculations were performed with the help of RIJCOSX approximation,^39^ with improved integral precision, enabled by “DEFGRID3” ORCA keyword, and strict convergence “TightSCF” settings. For visualization, the software Avogadro^40^ and MERCURY^40^ were used. Tanabe-Sugano diagrams were created with the help of a web application.^41^

### EPR Spectroscopy

The first derivative EPR spectra of powder samples were measured on an EMX Plus EPR spectrometer (Bruker BioSpin, Germany) in the temperature range 100–300 K. The samples were packed inside the thin-walled quartz EPR tubes and then precisely positioned within the microwave cavity by a special procedure as was described previously.^42^ The temperature was controlled by a Bruker temperature control unit ER 4111 VT with liquid nitrogen as the refrigerant. To examine a possible hysteresis of the thermally induced SCO effect, the temperature was cycled between 300 and 100 K three times. The temperature was changed in steps of 5 K with the time-delay of 5 min before each EPR measurement. The EPR spectra were processed, evaluated, and analyzed by Bruker software WinEPR.^43^ The relative integral intensity (*I*) of LS (*S* = 1/2) iron­(III) state EPR lines was calculated by the numerical double integration of the first derivative EPR spectra, with an error of 5% or less for LS state but 20% or more for EPR lines of HS state. Further details are given in our previous paper.^44^ The spin-Hamiltonian parameter values, which were obtained from the experimental EPR spectra, were further refined by computer simulation. The EPR spectra of LS state systems (*S* = 1/2) were computed using Bruker software SimFonia,^45^ while the EPR spectra of HS state systems (*S* = 5/2) were calculated by the original program “Spin”.^46^

## Results and Discussion

### Synthesis, Spectral, and Structural Characterization

The synthesis and characterization of the reported compounds are detailed in the Electronic Supporting Information (ESI). The hexadentate ligands H_2_L_n_ were prepared via Schiff base condensation of either 2-hydroxy-3,5-dimethylbenzophenone (H_2_L_1_= *N*,*N*′-bis­((2-hydroxy-3,5-dimethyl-phenyl)­phenyl)­methylidene-1,8-diamino-3,6-diazaoctane) or 2-hydroxy-5-methoxybenzo-phenone (H_2_L_2_=*N*,*N*′-bis­((2-hydroxy-5-methoxyphenyl)­phenyl)­methylidene-1,8-diamino-3,6-diaza-octane) with triethylenetetramine in a methanol/acetonitrile (1:1, v/v) mixture (Scheme S1, ESI). The resulting yellow solutions of the in situ-formed ligands were then reacted with anhydrous iron­(III) chloride in the presence of triethylamine as a base. Counterion exchange was performed using potassium selenocyanate (**C1**, **C4**), potassium thiocyanate (**C2**), or sodium tetraphenylborate (**C3**). Dark violet crystals of **C1**–**C4**, suitable for single-crystal X-ray diffraction (SCXRD) analysis, were obtained after several days of slow evaporation at room temperature. FT-IR spectroscopy of **C1**–**C4** (Figure S1) displayed characteristic absorption bands at 3224–3102 cm^–1^ (N–H stretching), 3059–3051 cm^–1^ (C_ar_–H stretching), 2998–2830 cm^–1^ (C_al_–H stretching), and 1602–1573 cm^–1^ (imine/*C*_ar_–*C*_ar_ vibrations). C≡N vibrations of SeCN^–^ and SCN^–^ were detected at 2063 cm^–1^ (**C1**), 2061 cm^–1^ (**C2**), and 2056 cm^–1^ (**C4**). Solid state UV/vis spectroscopy of **C1**–**C4** (Figure S2) revealed the presence of two intraligand (π → π* and n → π*) absorption bands in intervals 245–262 and 356–388 nm and one broad charge transfer band in the region 513–632 nm.

Thermogravimetric investigation (Figure S3) revealed about 8% (for **C1**), 7% (for **C2**) decrease of weight in the temperature range 308–370 K corresponding to the loss of about 1.3 and 1.5 molecules of acetonitrile per molecule of complex, respectively. This can be explained by the presence of additional lattice solvent molecules in **C1,** which were not possible to identify by structural refinement (vide infra), and by partial liberation of solvents before the thermogravimetric investigation in the case of **C2**. Nevertheless, the complete desolvation is obviously fully accessible within the temperature range of magnetic measurements (vide infra) and both desolvated compounds **C1d** and **C2d** are stable with further heating up to 493 K (220 °C). In contrast, the TGA of **C4** shows that solvent release begins only above 373 K (100 °C), which is significantly higher than those for **C1** and **C2**. Full desolvation of **C4** is achieved above 400 K and corresponds to a mass loss of ∼5%, consistent with the loss of one acetonitrile molecule per complex unit.

SCXRD analysis at **100 K** revealed that the reported coordination compounds crystallize in the **monoclinic** *P*2_1_/n space group (**C1**, **C2**, and **C4**) and the **orthorhombic** *Pcca* space group (**C3**). While the single crystals of **C1** remained stable during desolvation at elevated temperatures, enabling structural characterization of desolvated phase **C1d** at 190 K (which also adopts the *P*2_1_/*n* space group), all attempts to determine the structure below the SCO transition temperature (vide infra) were unsuccessful due to the loss of crystallinity upon cooling. Similarly, structural characterization of desolvated compound **C2d** proved impossible for the same reason. Selected crystallographic parameters are summarized in Table S2 (ESI). The asymmetric unit of **C1**, **C1d**, **C2**, and **C4** consists of one complex cation, one counteranion, and corresponding lattice solvent molecules. Their molecular structures can be expressed by formulas [Fe­(L_1_)]­SeCN·CH_3_CN (**C1**); [Fe­(L_1_)]­SCN·2CH_3_CN (**C2**) and [Fe­(L_2_)]­SeCN·CH_3_CN (**C4**). The asymmetric unit of **C3** contains half of the molecule [Fe­(L_1_)]­BPh_4_. The four molecules of each compound are involved in the unit cell.

Each complex cation contains corresponding Schiff base ligand anion coordinated on central atom via two phenoxy oxygen donor atoms, two imino (N^im^) and two amino (N^am^) nitrogen donor atoms forming the coordination environment {Fe­(N^im^)_2_(N^am^)_2_O_2_} (Figure 2). In agreement with previously reported iron­(III)-*saltrien* complexes, two oxygen donor atoms and two imino nitrogen donor atoms adopt the *cis*-O_2_/*trans*-N_2_^im^ and *mer*-{N^im^,N^am^,O} configuration. The presence of an inversion center in all structures results in both Δ and Λ enantiomeric forms occurring within the crystal lattice (Figure S4). A comparison of the crystal structures of **C1**–**C4** with those of **C1d** reveals distinct differences in their coordination polyhedra. For **C1–** **C4**, the bond lengths (Table S3) fall within the ranges of 2.00–2.01 Å (Fe–N^am^), 1.94–1.96 Å (Fe–N^im^), and 1.83–1.87 Å (Fe–O), consistent with a LS state of the iron­(III) center at 100 K. In contrast, **C1d** displays markedly elongated bonds of 2.16–2.19 Å (Fe–N^am^), 2.13–2.16 Å (Fe–N^im^), and 1.88–1.89 Å (Fe–O), characteristic of a HS iron­(III) metal center at 200 K. The average bond length differences between **C1d** (HS) and **C1** (LS) structures (Δ*d*(Fe–N^am^) ≈ 0.17 Å; Δ*d*(Fe–N^im^) ≈ 0.18 Å) are notably larger than those reported for analogous iron­(III)-*saltrien* complexes (Δ*d*(Fe–N^am^) ≈ 0.14 Å - 0.15 Å, Δ*d*(Fe–N^im^) ≈ 0.13–0.15 Å; Table S1). This pronounced structural response to spin-state switching suggests an enhanced ligand flexibility in the complex cation [Fe­(L_1_)]^+^. Quantitative analysis of polyhedral distortion using Σ and Θ parameters,^47^ along with SHAPE analysis,^48^ reveals minor deviations from ideal octahedral geometry in **C1**–**C4** (*S*(Oh) ≈ 0.3–0.4; Σ ≈ 45°–49°; Θ ≈ 80°–92°; Tables S4 and S5). These distortions become significantly more pronounced in **C1d** (*S*(Oh) = 3.2; Σ = 112°; Θ = 294°), confirming the LS state in **C1**–**C4** and the HS state in **C1d**. Additionally, the α angle between the least-squares planes of the phenolate rings further supports the LS configuration in **C1**–**C4** (69°–80°) and the HS configuration in **C1d** (106°).

**Figure 2:**
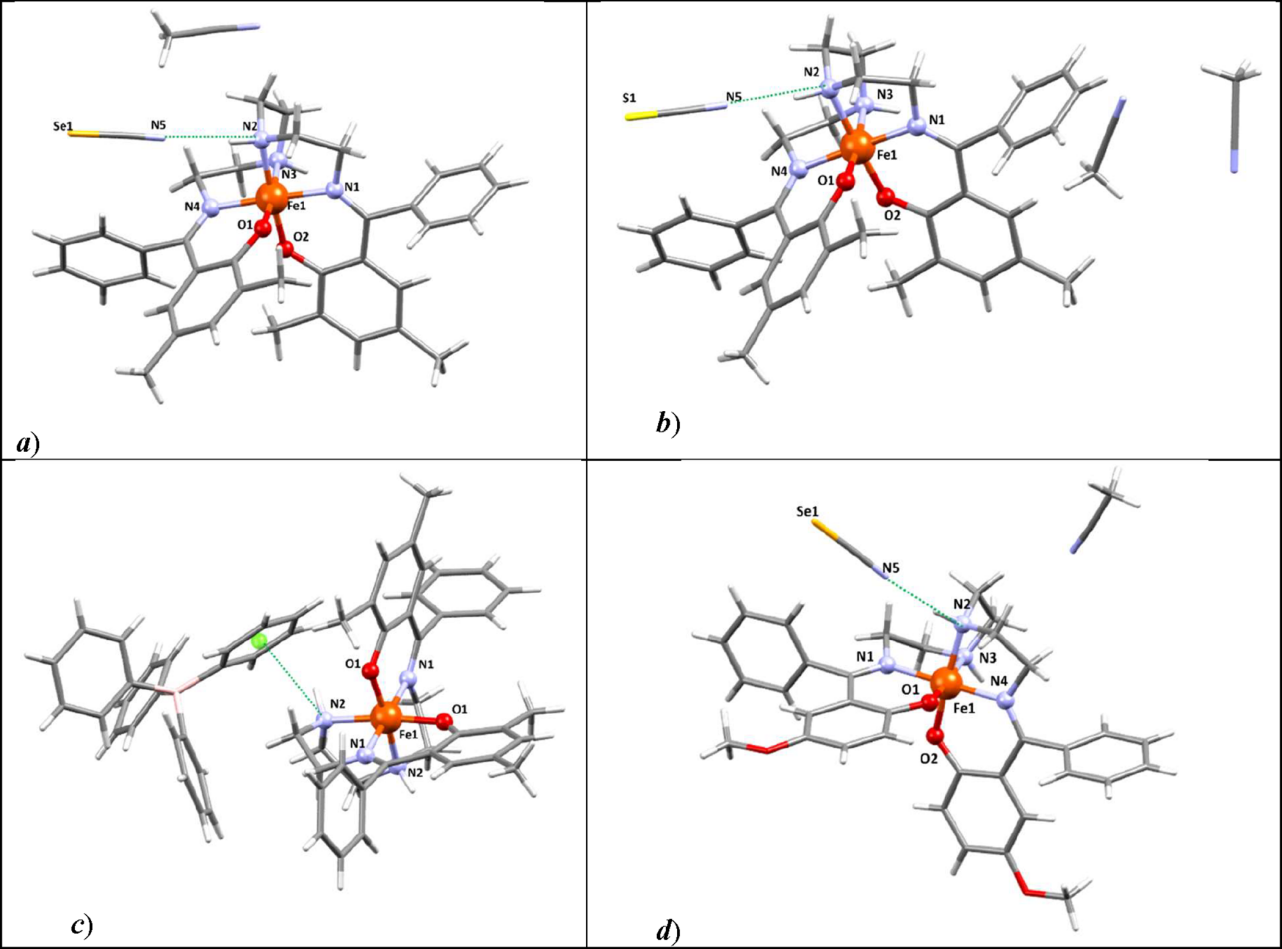
Molecular structures of (a) **C1**, (b) **C2**, (c) **C3** and (d) **C4**. Color code: C-gray, O-red, N-blue, Se-mustard yellow, S-pale yellow, B-pink, green dashed lines present noncovalent interactions, green ball-centroid of phenyl ring.

The crystal structures of **C1**, **C1d**, and **C2** exhibit similar noncovalent interactions responsible for the formation of supramolecular zigzag chains along the *a–b* (**C1**, **C2**) or *a–c* plane (**C1d**) (Figure S5a–c). The corresponding pseudohalide counteranions bridge neighboring complex cations via hydrogen bonds involving the secondary amino group of the Schiff base ligand. While the hydrogen bonds between the pseudohalide nitrogen atom (N5) and the amino group (N2–H2) are comparable across all three compounds (N5···N2 = 3.008(14) Å for **C1**, 2.906(14) Å for **C1d**, and 2.985(3) Å for **C2**), the short contacts between chalcogen atoms and the N3–H3 amino group are notably weaker and diminish upon substitution of sulfur with selenium (Se1···N3 = 3.397(7) Å for **C1**, 3.445(7) Å for **C1d**, and S1···N3 = 3.3262(19) Å for **C2**). A supramolecular zigzag chain along the *a–c* plane is also observed in the structure of **C3**, where neighboring complex cations are linked via weak interactions between the secondary amino group (N2–H2) and the benzene rings of the BPh_4_^–^ counteranion (centroid of C19–C24···N2 = 3.271(4) Å; Figure S5d). In contrast, the structure of **C4** features hydrogen bonds between the secondary amino group of the Schiff base ligand and the nitrogen atom of the NCSe^–^ anion (N5···N3 = 2.914(4) Å). However, no formation of a 1D supramolecular chain is observed, as the selenium atoms of NCSe^–^ do not participate in interactions with the amino group of neighboring complex cations (Se1···N2 = 5.082(2) Å).

The structures of the reported compounds also differ in their packing arrangements, as visualized in Figure S6. In **C1**, the [Fe­(L_1_)]^+^ cations form alternating sheets in the *a*–*b* plane, with interlayer spacings of 6.082(13) and 6.508(13) Å. The shortest intralayer Fe···Fe distance is 11.767(2) Å, while the longest interlayer separation is 9.027(2) Å. **C1d** displays a similar layered architecture but with shorter interlayer distances of 5.516(13) and 6.250(13) Å. The intralayer Fe···Fe spacing remains nearly identical at 11.772(3) Å, whereas the interlayer Fe···Fe distance decreases to 8.894(2) Å. **C2** also features alternating sheets in the *a*–*b* plane with interlayer spacings of 5.977(4) and 6.609(4) Å. The intralayer Fe···Fe distance is 11.7551(9) Å, and the shortest interlayer separation increases to 9.0602(7) Å. In **C3**, the [Fe­(L_1_)]^+^ cations form parallel tail-to-tail layers separated by 4.500(5) Å. The shortest intralayer and interlayer Fe···Fe distances measure 13.0714(6) and 10.7069(5) Å, respectively. Each cationic bilayer is further separated by bulky BPh_4_^–^ anion layers, which introduce an additional spacing of 11.124(6) Å. A second sequence of layers is also arranged along the *a*–*b* plane with an interlayer separation of 8.174(6) Å. For **C4**, alternating sheets of [Fe­(L_2_)]^+^ cations again appear in the *a*–*b* plane, with interlayer distances of 6.124(6) and 4.552(6) Å. The shortest intralayer Fe···Fe separation is 10.3475(7) Å, and the shortest interlayer distance is 8.4922(8) Å. Additionally, the connectivity between neighboring layers differs among the structures. In **C1**, **C1d** and **C2**, the adjacent layers of [Fe­(L_1_)]^+^ cations are linked via hydrogen bonds between the pseudohalide anions and the secondary amino groups of the Schiff base ligands. In **C3**, the [Fe­(L_1_)]^+^ cationic layers form short noncovalent contacts with adjacent layers of BPh_4_^–^ anions. In **C4**, however, the layers of [Fe­(L2)]^+^ cations do not engage in any significant intermolecular contacts with neighboring layers.

The free void and pore volume within the crystal lattices of the reported compounds was evaluated using the MoloVol software^49^ (Table S6). Calculations were performed on two sets of crystallographic data, and the total unoccupied volume was estimated as the sum of probe-excluded voids and regions accessible to small and large probes. First, the final refined structures were used, yielding unit-cell free volume fractions of 31% (**C1**), 32% (**C1d**), 27% (**C2**), 28% (**C3**), and 28% (**C4**). We also performed calculations on modified models of **C2** and **C3** in which structural disorders were removed to avoid artificially increased molecular volumes. Additionally, to better reflect the isostructural relationship between **C1** and **C2**, we modified the structure of **C2** by removing the second cocrystallized acetonitrile molecule. This adjustment aimed to achieve a more comparable solvation state and a more realistic estimation of the free volume in these two compounds. For these modified crystal structures, the free volume fractions increased to 32 and 34%, for **C2** and **C3**, respectively. In this corrected form, the values reflect the anticipated structural similarity between **C1** and **C2**. The comparable free volumes for **C1** and **C2** further suggest similar internal chemical pressures,^50^ which are noticeably lower than in **C4**. The largest free volume is observed in **C3**, attributable to the bulky BPh_4_^–^ anions occupying substantial space. However, the densely packed [Fe­(L1)]^+^ cationic layers in **C3** (Figure S6d) indicate that the internal chemical pressure within these cationic sublattices remains significant and may not be fully captured by the applied calculations.

### Hirshfeld Surface Analysis and QT-AIM Calculations

To quantitatively correlate structural features with intermolecular interactions in reported crystal structures, we performed a Hirshfeld surface analysis. The results substantiate the structural characteristics discussed previously. Using CrystalExplorer,^29^ we generated both *d*_norm_-mapped Hirshfeld surfaces (Figures S7–S11) and 2D fingerprint plots (Figures S12–S16) for crystal structures **C1**-**C4** and **C1d**. Intense red areas on the surfaces *d*_norm_ (with intermolecular contacts closer than van der Waals radii) indicate the presence of significant H···N, H···Se or H···S hydrogen bonds in **C1**, **C1d**, **C2** and **C4**. Additionally, **C4** exhibits H···O short contacts due to the methoxy group. The crystal structure of complex **C3**, due to the presence of the BPh_4_^–^ anion, does not contain these hydrogen bonds, which is also reflected in the Hirshfeld surfaces. Other close contacts are mainly of the H···H or H···C type and are prevalent in all crystal structures.

The 2D fingerprint plots provide a breakdown of contact types as a percentage of the total Hirshfeld surface area. A common feature of **C1**, **C1d**, **C2**, and **C4** is the presence of two sharp spikes at low *d*_e_/*d*_i_ values, corresponding to prominent H···N and H···Se/S contacts, which represent all observed hydrogen bonds (vide supra). Notably, in **C4**, the H···Se peak is less pronounced, while **C1d** exhibits an additional distinct peak associated with H···H contacts. These peaks are absent in **C3**, which lacks H···N and H···Se/S interactions. Instead, **C3** displays an elevated proportion of H···C contacts, reflecting the presence of CH/π and NH/π interactions involving the BPh_4_^–^ anion. The percentage contributions of individual contact types are illustrated in Figure 3 and detailed in the Supporting Information (Figures S12–S16). Across all structures, H···H and H···C interactions dominate the Hirshfeld surfaces, contributing the largest share of surface coverage. These contacts are particularly prominent in **C3**, where the bulky BPh_4_^–^ anion forms such interactions to a greater extent than those in the other structures containing smaller pseudohalide counterions. H···N interactions account for approximately 7% of the surface in solvated structures (**C1**, **C2**, and **C4**) but decrease to 5.5% in the desolvated **C1d**. The hydrogen-bonded networks in **C1**, **C1d**, **C2**, and **C4** are primarily stabilized by H···Se (∼4%) and H···S (2.6%) interactions.

**Figure 3:**
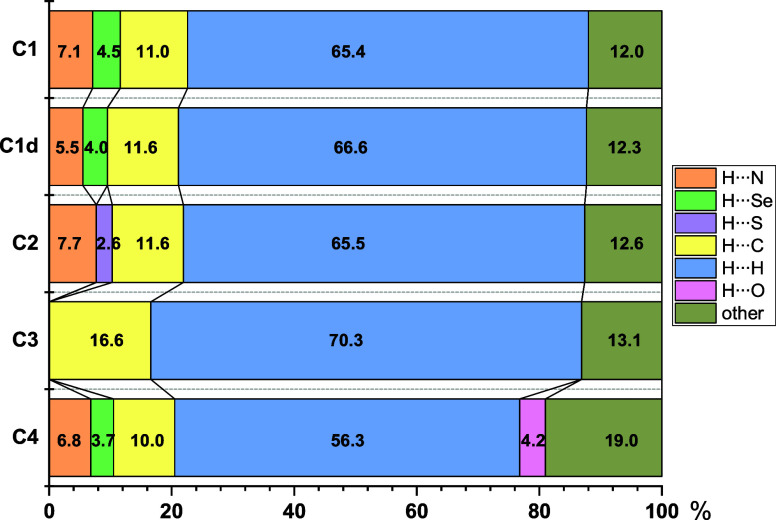
Percentage contributions of interactions for the reported crystal structures.

The quantitative analysis of noncovalent interactions present in the crystal structures of the studied complexes was performed using Quantum Theory Atoms In Molecules (QT-AIM) calculations (see Part S7 in ESI).^51^ Selected fragments from experimentally determined crystal structures were used to compute wave functions via DFT calculations,^33b,39a,39c,53^ carried out with the ORCA 6.0.0 software package.^33a^ The B3LYP functional^34,54^ and the Ahlrichs triple-ζ polarized basis set def2-TZVP were employed.^35b^ QT-AIM analyses and their visualizations were performed using the Multiwfn^55^ and AIMAll software packages.^56^

We focused on evaluating the first and second coordination spheres of the complexes. All four complexes contain two sets of donor atoms capable of forming not only coordination bonds but also noncovalent interactions of significant strengths, specifically, secondary amine groups as potential hydrogen bond donors and phenolic oxygen atoms as potential acceptors. In a previously studied series of similar Fe­(III) SCO complexes, [Fe­(napet)­(NCS)]·S, (H_2_napet = *N*,*N*′-[1,6-diamino-4-azahexane]­bis­(2-hydroxy-1-naphthaldimine), S = solvent molecule), we demonstrated that hydrogen bonding involving these atoms can significantly influence both the occurrence and the critical temperature of SCO.^57^ Specifically, stronger N–H···O/N hydrogen bonds were found to increase *T*_1/2_, while weak hydrogen bonding at these sites could suppress SCO entirely. Compounds having interaction energies (*E*_int_) of their N–H···O/N hydrogen bonds lower than 4 kcal.mol^–1^ did not exhibit SCO. Furthermore, the formation of a strong O–H···O hydrogen bond between the cocrystallized alcohol molecule and the phenolic oxygen atoms was shown to effectively quench SCO.

The first coordination sphere in **C1–C4** consists of two Fe–N^am^, two Fe–N^im^ bonds, and two Fe–O bonds. As described in the structural section of this article, the metal–ligand bond lengths are consistent with the LS state at the temperatures of the measurement. Therefore, all DFT and QT-AIM calculations were performed on the following LS structural fragments, which include the complex molecules along with the anions or solvent molecules forming hydrogen bonds with the amine group: {[Fe­(L_1_)]·2NCSe}^−^ (**C1**), {[Fe­(L_1_)]·2NCS}^−^ (**C2**), {[Fe­(L_1_)]·2BPh_4_}^−^ (**C3**) and [Fe­(L_2_)]·NCSe·CH_3_CN (**C4**). The phenolic oxygen atoms do not form any significant noncovalent interactions in the crystal structure of **C1–C4**. In all complex cations, the metal–ligand bonds exhibit significant covalent character, as indicated by the ratio of potential energy density to kinetic energy density (-*V*(**r**)/*G*(**r**)^58^ at the (3,-1) bond critical points (BCPs), which exceeds 1 for bonds of covalent nature (Tables S7–S10). The highest -*V*(**r**)/*G*(**r**) ratios were calculated for the Fe–N^am^ bonds (1.15 – 1.17), followed by slightly lower values for the Fe–N^im^ bonds (1.12–1.14). The Fe–O bonds showed the lowest covalency with ratios in the range of 1.10–1.11. Interestingly, the estimated interaction energies, calculated as *E*_int_ = *V*(**r**)/2,^59^ show the opposite trend. The largest |*E*_int_| values were found for the Fe–O bonds (59.8–68.3 kcal.mol^–1^), while the values for the Fe–N^im^ bonds were slightly larger than those calculated for the Fe–N^am^ bonds (48.6–52.2 vs 41.9–44.1 kcal.mol^–1^).

The N–H···N and N–H···S/Se hydrogen bonds in complexes **C1**, **C2**, and **C4** exhibit varying interaction energies. In **C1** and **C2**, both hydrogen bonds formed by the amine group connect to the respective pseudohalide anions and in both cases, |*E*_int_| are larger for the N–H···N (4.40 kcal/mol in **C1**, 4.77 kcal/mol in **C2**) than for the N–H···S/Se hydrogen bonds (3.67 kcal/mol in **C1**, 3.79 kcal/mol in **C2**). Overall, the |*E*_int_| values are slightly larger in **C1** than in **C2**. In **C4**, two N–H···N hydrogen bonds are present: one between the amine group and thiocyanate anion, and another involving a cocrystallized acetonitrile molecule. These interactions differ significantly in strength, with the charge-assisted hydrogen bond to the NCS^–^ anion being much stronger (6.68 kcal/mol) than that involving the neutral acetonitrile molecule (1.57 kcal/mol). Compound **C3** forms N–H···π interactions with tetraphenylborate anions, which exhibit relatively low |*E*_int_| (both at 2.25 kcal/mol). These results show that the interaction strengths vary considerably, with most exceeding 4 kcal/mol. The only exceptions are compound **C3**, which, as expected for N–H···π interactions, shows relatively weak interactions, and one of the N–H···N hydrogen bonds in **C4**, which is even weaker.

Direct comparison of *E*_int_ values calculated for compounds **C1–C4** with those reported for the [Fe­(napet)­(NCS)]·S series, and their respective roles in controlling *T*_1/2_, is not straightforward. The **C1–** **C4** compounds substantially differ in several structural aspects, including ligand architecture (e.g., two N–H groups instead of one), crystal symmetry, and the identity and number of cocrystallized solvent molecules. Even the most structurally similar compounds within the present series, **C1** and **C2**, which are nearly isostructural and share the same space group and similar unit cell parameters (Table S2), differ in the number of cocrystallized solvent molecules and the presence of anion disorder. Given these differences, it is unsurprising that no clear trend emerges between the calculated interaction energies and the experimentally observed *T*_1/2_ (vide infra). This suggests that the differences in crystal packing and overall structural organization affect more strongly the thermodynamic parameters (Δ*H* and Δ*S*) that govern *T*_1/2_ via the relationship *T*_1/2_= Δ*H*/Δ*S*.^12^ While hydrogen bonding interactions such as N–H···N and N–H···S/Se are present and often relatively strong, their impact on *T*_1/2_ appears to be secondary to the effects of crystal structure in the **C1–** **C4** series.

### Magnetic Properties and Theoretical Investigation

Variable temperature magnetic investigations of **C1** and **C2** revealed a solvent-dependent SCO behavior (Figure 4a). In the low-temperature range (2–200 K), the χT products are 0.40 cm^3^·mol^–1^·K (**C1**) and 0.56 cm^3^·mol^–1^·K (**C2**), slightly higher than the expected spin-only value for an *S* = 1/2 system (0.375 cm^3^·mol^–1^·K). The further increase of temperature cause an increase of χ*T* values, which indicates the presence of thermal SCO. However, this transition is accompanied by irreversible loss of lattice solvent molecules above room temperature and therefore is irreversible. The χ*T* values at 400 K (4.05 and 4.31 cm^3^·mol^–1^·K for **C1** and **C2**, respectively) suggest complete LS → HS conversion. In contrast, compounds **C3** and **C4** exhibit reproducible magnetic behavior upon heating to 400 K. Both remain in the LS state up to approximately 250 K, with χ*T* values reaching 0.47 (**C3**) and 0.66 (**C4**) cm^3^·mol^–1^·K. Further heating leads to a gradual increase in χ*T*, reaching values of 1.91 cm^3^·mol^–1^·K (**C3**) and 1.48 cm^3^·mol^–1^·K (**C4**) at 400 K, consistent with the onset of SCO, while subsequent cooling cycles showed no significant differences in magnetic behavior (Figure S21a). Notably, the stability of **C4** during thermal cycling reflects the higher temperature required for acetonitrile release in this compound compared to **C1** and **C2** (Figure S3). The LS plateaus for **C1**–**C4** were analyzed using the PHI software package,^60^ and the fitting results are summarized in Table S11 and visualized in Figure S22. For **C1**, a satisfactory fit was achieved by using only the isotropic *g*-factor. However, fitting the magnetic data for the other complexes required additional parameters. As previously noted, the χ*T* values for **C2** and **C3** exceed those expected for *S* = 1/2 systems. To account for this, temperature-independent paramagnetism (χ_TIP_) and a minor contribution from an *S* = 5/2 magnetic impurity (*x*_IMP_) were included to maintain reasonable *g*-factor values for **C2** and **C3**. Additionally, complexes **C2**-**C4** show a decrease in χ*T* at low temperatures, which is attributed to intermolecular antiferromagnetic exchange interactions; this was modeled by using the *zJ* parameter. As a result, the obtained parameters vary in the ranges *g*_iso_: 2.103 – 2.197, χ_TIP_: 0.181·10^–3^–0.246·10^–3^ cm^3^ mol^–1^, *x*_IMP_: 0.025–0.049, and *zJ*: −0.029 to −0.260 (Table S11).

**Figure 4:**
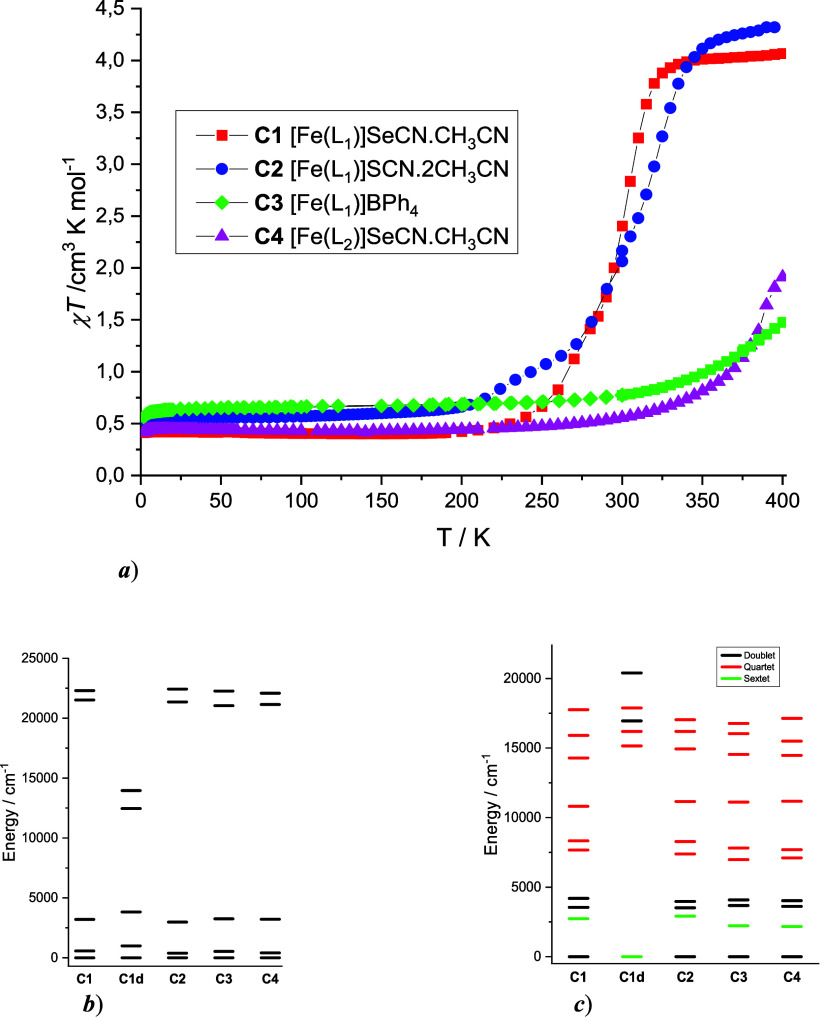
(a) Magnetic properties of **C1–** **C4**. CASSCF/NEVPT2 computed energies of (c) *d*-orbitals and (b) lowest LF terms calculated from X-ray structures of **C1–** **C4** and **C1d**.

To further elucidate the magnetic properties of the reported compounds, quantum chemical calculations were performed. Multireference CASSCF/NEVPT2 computations were performed on the LS structures **C1**-**C4** and on the HS structure **C1d**, all of which were obtained from X-ray diffraction analysis. The ab initio ligand field (AILFT) module^61^ was employed to extract the energies of the *d*-orbitals (Figure 4b). AILFT calculations provided access to the Racah parameters *B* and *C* (Table S12), as well as the ligand field splitting parameter 10 Dq, estimated as the average energy difference between the *t*_2g_ and *e*_g_ sets of orbitals. The calculated ligand field splitting patterns reveal no significant differences among **C1**–**C4**, consistent with their similar LS behavior up to room temperature. The computed ligand field terms confirm a ground-state doublet ^2^ *T*_2g_ and a first excited sextet ^6^ *A*_1g_ state (Figure 4c), separated by approximately 2200 cm^–1^ for **C3** and **C4**, and around 2731 cm^–1^ for **C1** and 2915 cm^–1^ for **C2**. This suggests that **C3** and **C4** should exhibit thermal SCO at lower temperatures compared to those of **C1** and **C2**. Consistent with the structural data, CASSCF/NEVPT2 calculations for **C1d** confirmed a HS configuration, revealing a well-isolated sextet ground state ^6^ *A*_1g_ with significant energy separation from higher-lying excited states.

Due to the deviation from perfect octahedral symmetry in the studied complexes, the *d* orbitals exhibit a nonideal splitting pattern. Nevertheless, the AILFT results show that the general distribution of *d* orbitals remains consistent with expectations for an ideal octahedral geometry and for the LS (**C1**–**C4**) and HS (**C1d**) states of the investigated structures. The *d*-orbital energy distributions and AILFT-derived ligand field parameters showed no significant variations across **C1**–**C4** (Table S12). However, **C3** and **C4** (10 Dq ≈ 20,400 cm^–1^) exhibited a modest reduction in 10 Dq values relative to those of **C1** and **C2** (10 Dq ≈ 20,670 and 20,772 cm^–1^, respectively), consistent with their narrower energy gap between the ground doublet and the first excited sextet term. As expected, the electronic structure of **C1d** is notably different, with the lower separation of *t*_2g_ and *e*_g_ orbitals, which results in a lower 10 Dq energy (≈11,605 cm^–1^). These trends are further supported by the visualization of Tanabe–Sugano diagrams (Figure S23) and the comparison between the Tanabe–Sugano and CASSCF energy levels (Figure S24). While the general trends agree across both approaches, minor discrepancies arise due to the real, distorted geometries of the complexes, which lead to the splitting and shifting of idealized ligand field energy levels in the CASSCF-derived terms.

The calculated ^2^ *T*_2g_–^6^ *A*_1g_ energy separations and 10Dq values indicate an increasing trend in *T*_1/2_ in the order **C4** ≈ **C3** < **C1** < **C2**, which does not reflect the experimentally observed behavior (Figure 4a). However, the computed energy differences across the series are modest: the largest ^2^ *T*_2g_–^6^ *A*_1g_ gap difference is 745 cm^–1^ (**C2** vs **C4**), and the maximum variation in 10Dq is 372 cm^–1^ (**C2** vs **C3**/**C4**). These values are relatively small and do not denote substantial changes in the ligand-field strength. Comparable variations in calculated ligand-field terms have been previously reported.^62^ It is important to note that the CASSCF calculations were performed using an active space that includes only the *d*-electrons of the Fe­(III) centers, excluding the π-electrons of the Schiff base ligands. In our earlier work,^2b^ we showed that delocalization of *e*_g_ orbitals into the aromatic π-system can notably influence the occurrence of SCO. In the present study, however, the CASSCF calculations were performed using a (5,5) active space that includes only the d-electrons of the Fe­(III) centers, excluding the π-electrons of the Schiff base ligands. As a result, all investigated compounds exhibit very similar ligand field characteristics within the AILFT framework, consistent across the series. While inclusion of ligand π-orbitals would likely improve alignment with experimental magnetic trends, it would simultaneously prevent the extraction of ligand-field parameters via AILFT. Additionally, ab initio calculations performed on isolated molecules do not account for supramolecular interactions or crystal packing effects, both of which play critical roles in determining SCO behavior in the solid state. Since hydrogen bonding could, in principle, affect the transition temperatures, we analyzed these interactions (vide supra); however, no correlation was found between hydrogen-bond metrics and the experimental *T*_1/2_ values. This supports the conclusion that hydrogen bonding is not the dominant factor and that crystal packing effects govern the observed trends. The influence of molecular and lattice rigidity, as a proxy for internal chemical pressure, is well recognized as a factor that can elevate the SCO temperature.^12,63^ As discussed in the structural section (vide supra), the packing of the SCO-active cations, reflected in interlayer arrangements and separations, free volume, and the closest Fe···Fe distances, is considerably denser in **C3** and **C4** compared to the isostructural pair **C1** and **C2**. This increased packing density likely accounts for the higher experimental *T*_1/2_ values observed for **C3** and **C4** relative to those of **C1** and **C2**.

To further investigate the SCO behavior of **C1**–**C4**, we performed DFT calculations using the OPBE functional^64^ to optimize both LS and HS geometries. The optimized structures show good agreement with experimental data for the LS forms, while the HS geometries display the expected bond elongation characteristic of SCO compounds (Tables S13 and S14), comparable to that observed in the HS structure of **C1d**. Crystal field parameters were calculated for all optimized structures (Table S15), with the corresponding *d*-orbital splitting patterns and ligand field terms shown in Figure S25. The parameters derived from the optimized LS geometries closely match those obtained from X-ray crystallography, whereas the transition to the HS state leads to a predictable decrease in the 10 Dq value. Importantly, both the LS and HS series exhibit consistent trends without significant deviations. Tanabe–Sugano diagrams based on these optimized geometries (Figure S26) indicate that the LS structures lie near the crossover point between the ^6^A and ^2^T ligand field terms, providing strong theoretical support for their observed SCO behavior.

Thermal cycling of **C1** and **C2** within the magnetic measurements enabled the formation of solvent-free **C1d** and **C2d** phases, consistent with thermogravimetric analysis showing complete acetonitrile removal at around 100 °C (vide supra). Both desolvated compounds remain in the HS state between 350 and 200 K (χ*T* = 4.07 cm^3^·mol^–1^·K for **C1d** and χ*T* = 4.13 cm^3^·mol^–1^·K for **C2d** at 300 K; Figure S21b). Upon further cooling, **C1d** exhibits a sharp, complete, one-step HS-to-LS transition, evidenced by a sudden χ*T* drop to 0.52 cm^3^·mol^–1^·K. Heating measurements reveal a stable thermal hysteresis loop over five consecutive cycles, with transition temperatures of *T*_1/2↑_ = 183 K and *T*_1/2↓_ = 166 K (Δ*T* = 17 K; Figures 5a and S21c). **C2d** also undergoes SCO below 200 K but in two distinct steps (Figures 5b and S21b,c). The first sharp step shows a 16 K hysteresis (*T*_1/2↑_ = 169 K and *T*_1/2↓_ = 153 K), slightly shifted to lower temperatures compared to **C1d**, while the second, more gradual transition is centered at *T*_1/2_ = 110 K. Analysis of χ*T* values indicates that ≈78% of **C2d** molecules participate in the abrupt, hysteretic SCO, while the remaining 22% undergo a gradual transition at lower temperatures. The decisive role of the lattice solvent in modulating SCO behavior has been extensively documented in the literature. Solvent molecules can modify packing density, internal chemical pressure, vibrational entropy, and cooperativity pathways within the crystal lattice, thereby shifting *T*_1/2_ values, altering hysteresis, or inducing multistep behavior.^65^ In particular, desolvation may trigger structural reorganization, partial amorphization, or phase transitions that strongly influence the magnetic response.^66^ Similar solvent-dependent SCO phenomena have been observed in ferrous and ferric systems where removal or exchange of lattice solvent led to abrupt transitions, loss or emergence of hysteresis, or significant temperature shifts in the HS⇄LS equilibrium.^67^ In our compounds, the contrasting behavior between solvated **C1**/**C2** and desolvated **C1d**/**C2d** is fully consistent with these established solvent-driven effects on supramolecular interactions and cooperativity.

**Figure 5:**
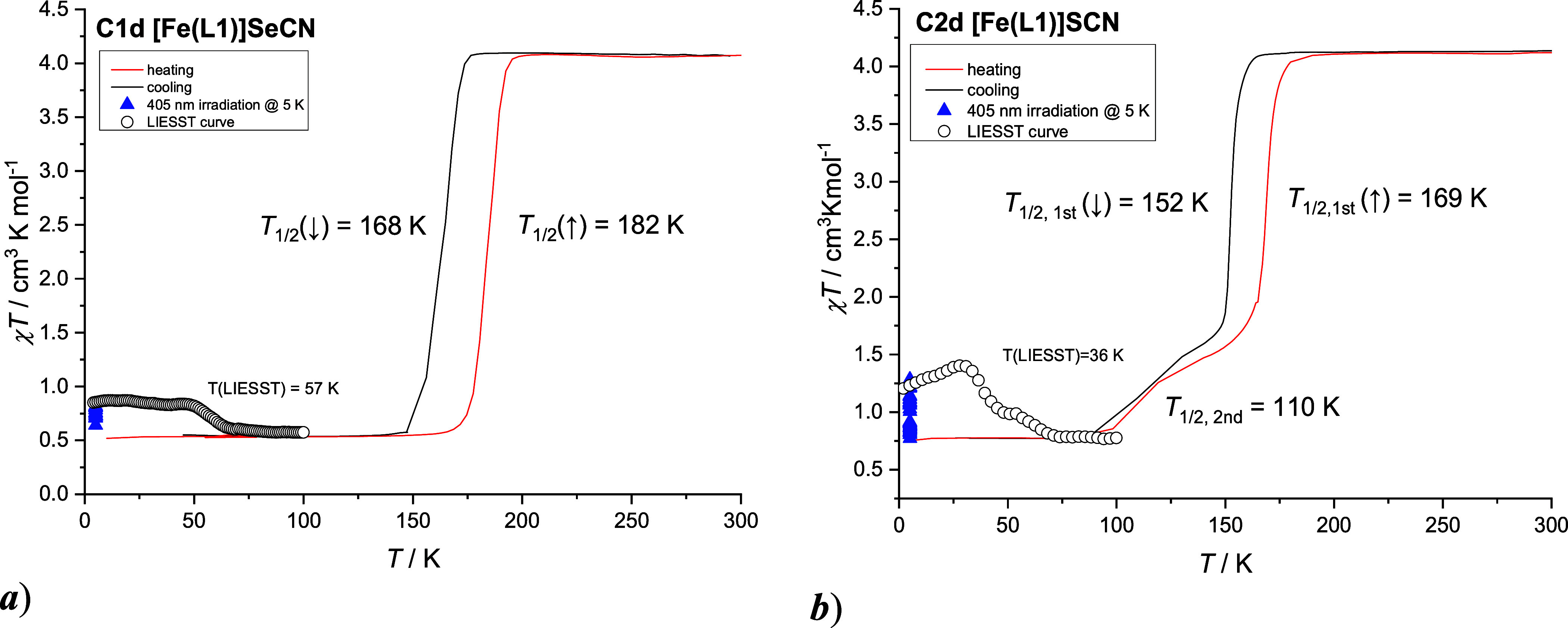
Temperature variable magnetic properties recorded within 4th heating and cooling cycle and photomagnetic properties for **C1d** (a) and **C2d** (b).

To probe photoinduced SCO, **C1d** and **C2d** dispersed in eicosane were irradiated at 5 K with red (637 nm), green (532 nm), and blue (405 nm) laser light. This starting temperature was chosen to facilitate the recording of χ*T* vs *T* curves from 5 K upward, allowing us to detect any very low-temperature phenomena, such as potential exchange interactions and ZFS in the photoexcited HS metastable state. Blue light yielded the highest LS-to-HS conversion efficiency, increasing χ*T* to 0.87 cm^3^·mol^–1^·K (**C1d**) and 1.4 cm^3^·mol^–1^·K (**C2d**), corresponding to ≈10 and ≈20% photoconversion of LS Fe­(III) centers, respectively. Such low efficiency of the light-induced LS-to-HS conversion observed in **C1d** and **C2d** is consistent with trends reported for other ferric SCO systems.^68^ This can be attributed to a combination of intrinsic molecular properties (i.e., deficient difference between the LS and HS Fe–N bond lengths) and experimental factors, including poor light penetration, a heterogeneous sample environment, or a suboptimal irradiation wavelength. Further measurements in the dark confirmed metastable HS-state stability up to *T*(LIESST) = 57 K (**C1d**) and 36 K (**C2d**). The small anomaly observed around 50 K in the LIESST curves of **C1d** and **C2d** is attributed to the antiferromagnetic ordering of trace adsorbed oxygen, a well-known and often persistent artifact in magnetic studies. The observation of LIESST effects in **C1d** and **C2d** is particularly noteworthy, as such a phenomenon remains rare for iron­(III) SCO compounds and its effectiveness correlates with structural distortion and intermolecular cooperativity.^69^ In the case of **C1d**, the pronounced distortion of the Fe­(III) coordination polyhedron, evidenced by significant changes in the Fe–N^am^ and Fe–N^im^ bond lengths between the LS (**C1**) and HS (**C1d**) structures (vide supra), may facilitate the light-induced transition. A similar explanation can be proposed for **C2d**, as temperature-dependent powder X-ray diffraction (vide infra) studies reveal that the desolvated compounds **C1d** and **C2d** in both LS and HS states are isostructural, suggesting a common structural framework conducive to the LIESST phenomenon.

### Temperature Variable X-ray Powder Diffraction and EPR Studies

To elucidate the solvent-dependent thermal SCO behavior observed in **C1**/**C1d** and **C2**/**C2d**, temperature-dependent powder X-ray diffraction (PXRD) studies were performed on powder emulsion dispersed in high viscosity oil using a single-crystal Synergy-i diffractometer. Both solvated samples, **C1** and **C2**, were first heated from 300 to 400 K (1st heating cycle), then cooled from 400 to 100 K (**C1**) or 90 K (**C2**) (1st cooling cycle), followed by a second heating cycle up to 400 K (Figures S27 and S28). Despite the limited resolution of the diffraction patterns, the diffractogram of **C2** recorded at the initial temperature of 300 K matches well with the calculated pattern from the SC-XRD data. In contrast, the diffractogram of **C1** at 300 K appears to be a superposition of the calculated patterns for **C1** and **C1d**, indicating that the partial loss of acetonitrile solvent had already occurred before the temperature-dependent experiment began. Upon heating, complete desolvation was observed, and the diffractogram of **C1** at 400 K after the second heating cycle matches that of the calculated pattern for **C1d**. A similar trend was noted for **C2**, whose final diffractogram at 400 K (after the second heating cycle) also corresponds to the calculated pattern for **C1d**, suggesting that the HS phases of **C1d** and **C2d** are isostructural (Figure S28g). The primary discrepancy between the experimental diffractograms of **C1d** and **C2d** at 100 K (LS, after the first heating and first cooling) and the calculated pattern for **C1d** (HS, at 190 K) is the low-angle reflection at 2θ ≈ 6.9°, which gradually diminishes with increasing temperature (Figures 6a and S27e,f). This indicates that the LS phases of **C1d** and **C2d** could potentially exhibit different crystal packing compared to their HS phases, a phenomenon often observed in SCO compounds that display steep thermal transitions and thermal hysteresis.^70^ The temperature dependence of the integrated area of this reflection enabled monitoring of the thermal SCO. Likewise, several other relatively well-separated reflections at 2θ ≈ 9.2°, 19.2°, and 21.1° showed increasing intensity upon heating and were also used to track the SCO event in **C1d** and **C2d** (Figure 6b). The calculated transition temperatures *T*_1/2_ are summarized in Table 2 and are consistent with those obtained from the magnetic measurements. Notably, the temperature-dependent behavior of all monitored peak areas indicates the presence of thermal hysteresis in both **C1d** and **C2d**. However, no evidence of a second SCO step was observed for **C2d**. This absence may be attributed to a partially amorphous phase formed upon desolvation, which lacks long-range order and thus does not contribute sharp reflections to the diffractogram (Figure S29d).

**Figure 6:**
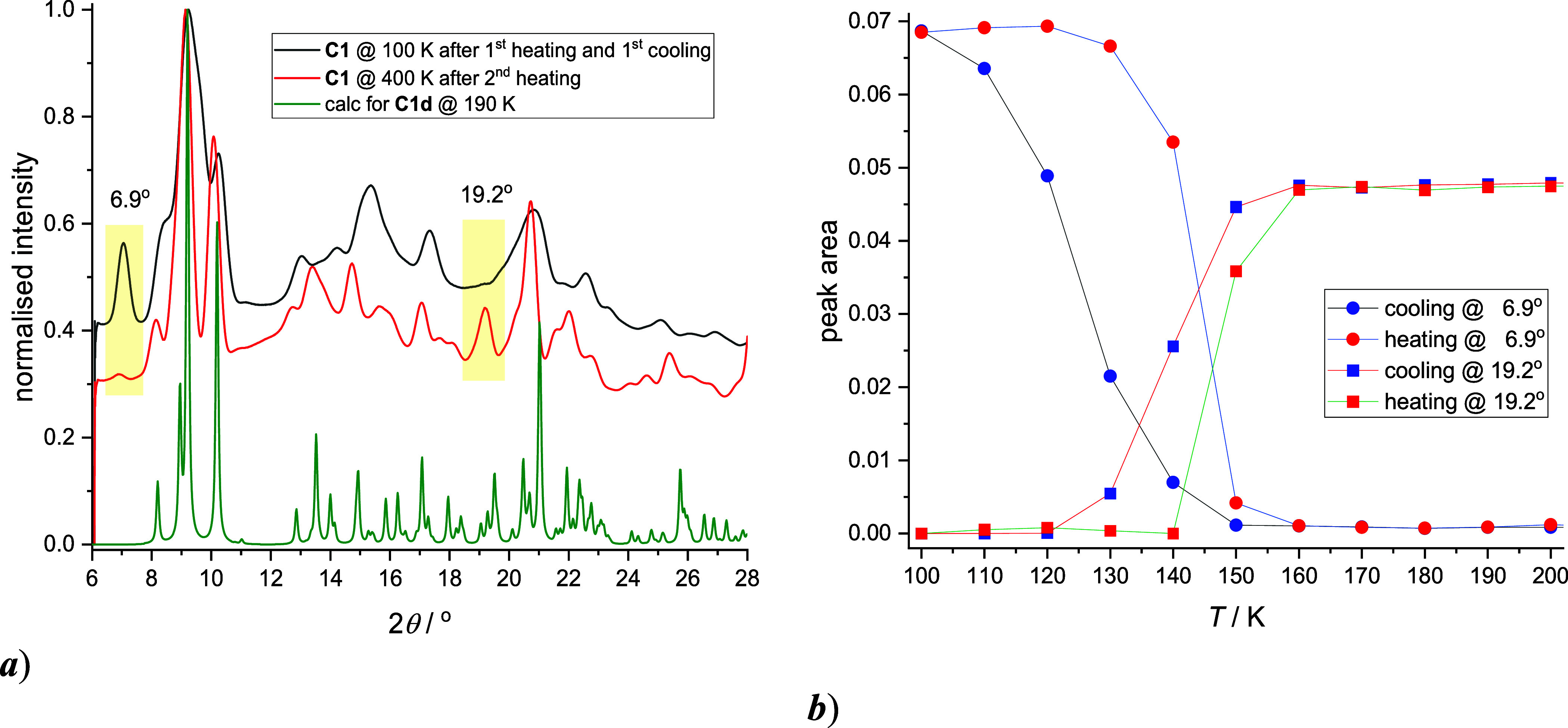
Temperature dependent PXRD experiments for **C1**: (a) comparison of diffractograms recorded at 100 and at 400 K (after 2nd heating cycle) and calculated pattern for structure **C1d**. (b) temperature dependent evolution of peak areas for diffractions at 6.9° and 19.2°.

Variable-temperature X-band EPR spectroscopy was employed to further investigate the spin states and thermal SCO behavior of the studied complexes. First derivative EPR spectra of powdered samples **C1d**, **C2d**, **C3**, and **C4** were recorded in the temperature range of 100–300 K, with spectra collected at the lowest and highest temperatures shown in Figure 7. At 100 K, all four complexes exhibit dominant and relatively sharp resonance signals located downfield near an effective *g*-value of ∼2.0. These rhombic EPR spectra are characteristic of LS iron­(III)-*saltrien* complexes.^15b,18c,18g^ Additionally, room temperature spectra of **C3** and **C4** retained this LS signal, indicating that these complexes remain in the LS state across the measured temperature range. In contrast, the room temperature spectra of **C1d** and **C2d** are dominated by broad pseudosinglet signals near an effective *g*-value of ∼4.3, along with a weaker shoulder at ∼6.0.

**Figure 7:**
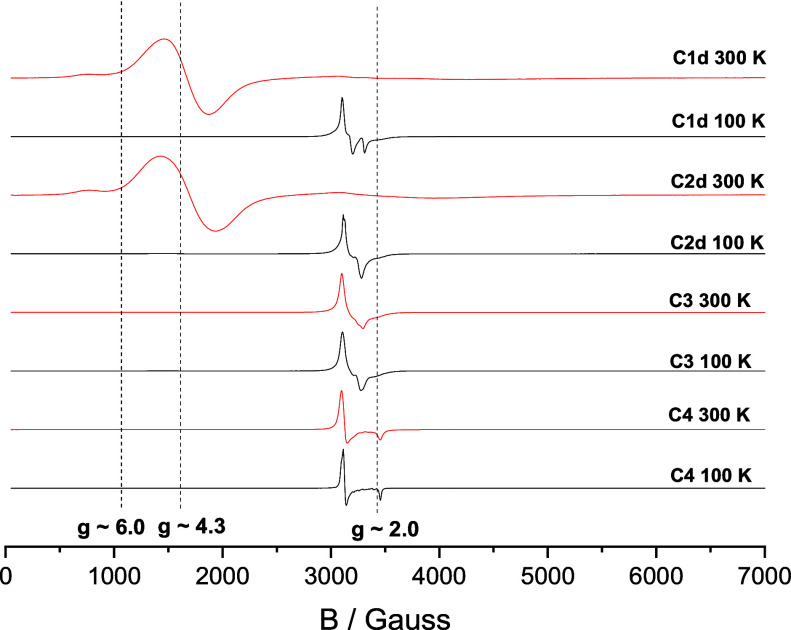
First derivative EPR spectra of **C1d**, **C2d**, **C3**, and **C4** recorded at 100 and 300 K. The typical effective *g*-factor values of 2.0, 4.3, and 6.0 are indicated by dot lines.

Such EPR spectra with ZFS features are characteristic of the HS iron­(III) centers. The spin Hamiltonian parameters derived from spectral simulations are summarized in Table 2. For **C1d** and **C2d**, the axial ZFS parameter (*D*) falls within a narrow range of 0.033–0.034 cm^–1^, while the rhombic parameter (*E*) remains negligible (≤0.001 cm^–1^). These values are typical for ferric complexes with distorted octahedral coordination and confirm the electronic nature of the HS state. The effective *g*-values for **C1d** and **C2d** (*g*_eff_ ≈ 4.038–4.039) also lie within the expected range for HS Fe­(III), further corroborating their spin-state assignment. In contrast, **C3** and **C4** display no significant differences between spectra recorded at 100 and 300 K, with *g*-values consistently near 2.0, confirming their persistent LS character and the absence of spin transition in this temperature interval. Importantly, the observed ZFS and effective *g*-values provide additional information beyond bulk magnetic susceptibility, since they directly reflect local anisotropy and ligand field distortions at the Fe­(III) centers, thereby complementing structural (SCXRD, PXRD) and magnetic data. The consistency of these parameters with literature values for LS and HS iron­(III) systems^3a^ validates our interpretation of the SCO process in **C1d** and **C2d**, while their reproducibility across thermal cycles supports the robustness of the observed transitions.

The temperature dependence of the integral intensity (*I*) of EPR resonance signals offers valuable insights into the relative populations of HS and LS iron­(III) centers, enabling reconstruction of the thermal SCO curves for compounds **C1d** and **C2d**. As expected, the integral intensity of the LS signal (*I*_LS_) increases, while that of the HS signal (*I*_HS_) decreases with lowering the temperature. A representative temperature profile of *I*_LS_ for **C1d** is shown in Figure 8a. Spectra recorded in the 100–130 and 190–300 K ranges exhibit LS and HS signals exclusively, respectively, indicating a complete and reversible SCO process in **C1d** without evidence of frozen spin states. The transition occurs between 130 and 185 K, with three consecutive cooling/heating cycles revealing a reproducible thermal hysteresis consistent with magnetic susceptibility data (vide supra). The transition temperatures were determined by fitting the data to a Boltzmann sigmoidal function, yielding *T*_1/2,↓_ = 151.1 ± 0.5 K (*r* ^2^ = 0.99) and *T*_1/2,↑_ = 169.3 ± 0.5 K (*r* ^2^ = 0.99), corresponding to a hysteresis width Δ*T*_1/2_ = 18.2 K, in agreement with magnetic data. Temperature-dependent X-band EPR measurements on **C2d** confirmed a two-step, hysteretic thermal SCO behavior (Figure 8b). Only HS signals were observed between 190–300 K, while spectra at 77 K displayed LS signals exclusively, confirming a complete and quantitative SCO. Upon cooling from room temperature, the first SCO step occurs abruptly, accompanied by thermal hysteresis that remains stable over three thermal cycles (Figure S30). The transition temperatures, determined from EPR data, are slightly higher than those obtained from magnetic susceptibility and PXRD measurements (Table 1), with *T*_1/2,↑_ = 177.8 ± 0.5 K (*r* ^2^ = 0.99) and *T*_1/2,↓_ = 162.4 ± 0.5 K (*r* ^2^ = 0.99). Further cooling reveals a second, more gradual SCO step centered around *T*_1/2_ = 120 K. These findings clearly demonstrate that EPR spectroscopy is a powerful technique for probing the hysteretic SCO behavior. Both **C1d** and **C2d** exhibit thermal LS ↔ HS switching consistent with the magnetic susceptibility results. The calculated transition temperatures *T*_1/2_ are summarized in Table 2

**Table 1:** Observed *T*_1/2_ Transition Temperatures from the Experimental Techniques Used

	1. step *T*_1/2_(↓)/K	1. step *T*_1/2_(↑)/K	2. step *T*_1/2_(↑)/K
**C1d**			
magnet^a^	166	182	
PXRD @ 6.9 °	125	140	
PXRD @ 19.2 °	139	149	
EPR^a^	151.1	169.3	
**C2d**			
magnet^a^	153	170	110
PXRD @ 6.9 °	134	144	not observed
PXRD @ 9.2 °	143	151	not observed
PXRD @ 19.2 °	146	153	not observed
PXRD @ 21.1 °	141	146	not observed
EPR^a^	162.4	177.8	120

a Average values from all recorded C/H cycles.

**Table 2:** Spin Hamiltonian Parameters Obtained from Simulations of the Experimental EPR Spectra^a^

		spin Hamiltonian parameters			
		*g*_1_ (±0.003)	*g*_2_ (±0.003)	*g*_3_ (±0.003)	|*D*| [cm^–1^] (±0.003)
**C1d**	300 K, HS	4.038*	4.038*	4.038*	0.033
	100 K, LS	2.168	2.121	2.033	
**C2d**	300 K, HS	4.039*	4.039*	4.039*	0.034
	100 K, LS	2.162	2.128	2.061	
**C3**	300 K, LS	2.167	2.061	2.058	
	100 K, LS	2.169	2.068	2.064	
**C4**	300 K, LS	2.165	2.164	1.951	
	100 K, LS	2.162	2.161	1.950	

a Note: the effective *g*-values are *-marked.

**Figure 8:**
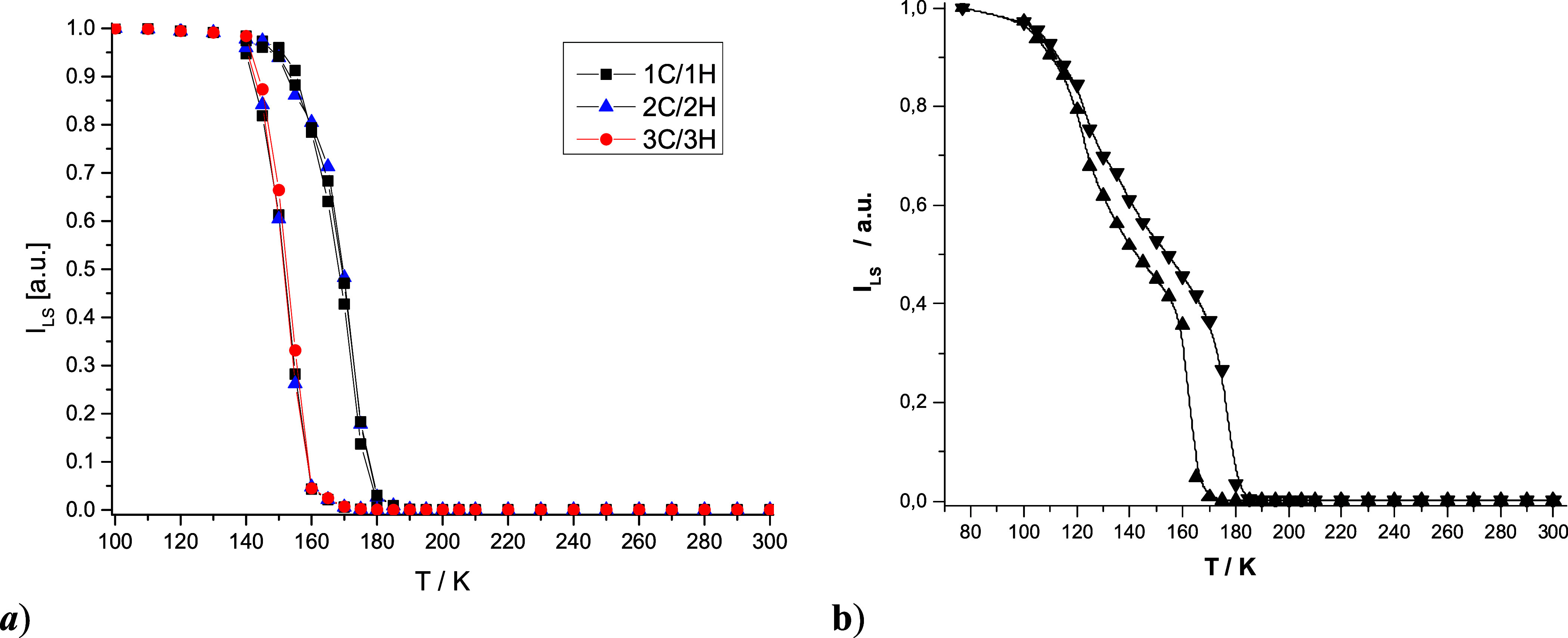
(a) Temperature dependence (three subsequent cooling/heating cycles) of relative integral intensity (*I*_LS_) of LS (*S* = 1/2) state resonance signal in the EPR spectra of **C1**. Note: The gaps between symbols were approximated by B-Spline. (b) Temperature dependence of the relative integral intensity (*I*_LS_) of the LS (*S* = 1/2) state resonance signal in the EPR spectra of **C2d**.

Differences in SCO parameters, such as *T*_1/2_ and hysteresis width, when determined by various experimental techniques (e.g., magnetic measurements, X-ray powder diffraction, and EPR spectroscopy), are commonly observed and arise from the distinct physical properties each technique probes.^71^ While all methods confirmed the presence of thermal SCO and hysteresis, magnetic measurements detect the bulk magnetic response of the ensemble of molecules, reflecting the average spin state. PXRD is sensitive to the macroscopic structural changes within the crystalline lattice that accompany the spin transition. In contrast, EPR spectroscopy probes the local electronic environment and spin state of individual paramagnetic metal centers. Consequently, each technique is affected differently by factors such as sample inhomogeneity, particle size distribution, and the kinetics of the spin transition, including potential thermal lag during dynamic measurements. The discrepancies thus reflect the unique sensitivity of each method to different aspects of the SCO phenomenon, providing complementary rather than contradictory insights into the complex spin state behavior.

## Conclusions

In this study, we successfully synthesized and thoroughly investigated four novel mononuclear iron­(III) coordination compounds featuring hexadentate *saltrien*-like Schiff base ligands. These ligands, derived from substituted 2-hydroxybenzophenones and triethylenetetramine, were designed to incorporate additional phenyl rings with the aim of enhancing intermolecular cooperativity in their iron­(III) compounds, an objective that was only partially achieved.

Magnetic measurements revealed that all four solvated compounds undergo thermally induced SCO at or above room temperature, in agreement with ab initio calculations of their electronic structures. However, the experimentally observed *T*_1/2_ values and SCO profiles do not directly correlate with calculated ^2^ *T*_2g_–^6^ *A*_1g_ separations and 10Dq, which vary only modestly across the series. Instead, the SCO characteristics are predominantly dictated by solid-state effects, specifically crystal packing, intermolecular connectivity, lattice rigidity, and internal chemical pressure. Upon heating, compounds **C1** and **C2** undergo reversible solvent loss, producing desolvated analogues **C1d** and **C2d**. This desolvation shifts their SCO to below-room-temperature and induces well-defined transitions with pronounced hysteresis. **C1d** displays an abrupt one-step SCO with a wide hysteresis loop, while **C2d** exhibits an additional gradual step in the low-temperature regime. Hirshfeld surface analysis, QT-AIM calculations, and variable-temperature PXRD confirm that desolvation increases the cooperativity and likely triggers a structural phase transition upon SCO, giving rise to the observed abrupt and hysteretic behavior. These conclusions are further supported by EPR spectroscopy. In contrast, **C3** and **C4** crystallize in more densely packed frameworks with reduced free volume, shorter Fe···Fe separations, and greater lattice rigidity, all of which stabilize higher *T*_1/2_ values. Although the bulky BPh_4_^–^ anion in **C3** introduces additional free volume, it also promotes tighter stacking of the [Fe­(L1)]^+^ cations, reinforcing cooperativity and shifting the SCO to higher temperatures. Across the entire series, hydrogen bonding plays a secondary role compared with packing effects and internal chemical pressure.

Beyond thermal control of SCO, both desolvated compounds **C1d** and **C2d** also exhibited photoinduced SCO under blue light irradiation, confirming the LIESST effect. The observation of photoinduced SCO in ferric complexes remains rare, and its presence in these systems is attributed to the pronounced flexibility of the hexadentate ligand, as evidenced by significant changes in the Fe–N^am^ and Fe–N^im^ bond lengths between the LS (**C1**) and HS (**C1d**) structures.

Overall, this work underscores the critical role of solvent molecules, counterions, and ligand substitution in modulating the SCO properties of iron­(III)–*saltrien* complexes. The discovery of bistable and photoswitchable behavior in these systems enhances their potential for future applications in molecular electronics, magnetic switching, and sensor technologies.

## Supplementary Material



## Notes

Review of reported iron­(III)-saltrien molecular compounds; spectral, structural, magnetic, and computational characterization, and EPR spectra (PDF)

## References

[1] a Gütlich, P. ; Goodwin, H. A. Spin Crossover in Transition Metal Compounds I–III: Topics in Current Chemistry; Springer Science+Business Media: 2004; Vols. 233–235.

[2] Masarova P., Zoufaly P., Moncol J., Nemec I., Pavlik J., Gembicky M., Travnicek Z., Boca R., Šalitroš I. (2015). Spin Crossover
and
High Spin Electroneutral Mononuclear Iron­(III) Schiff Base Complexes
Involving Terminal Pseudohalido Ligands. New
J. Chem..

[3] Pogány L., Brachňaková B., Moncol J., Pavlik J., Nemec I., Trávníček Z., Mazúr M., Bučinský L., Suchánek L., Šalitroš I. (2018). Impact of Substituent Variation on
the Presence of Thermal Spin Crossover in a Series of Mononuclear
Iron­(III) Schiff Base Complexes with Terminal Pseudohalido Co-Ligands. *Chemistry – A*. European Journal.

[4] Šalitroš I., Pavlik J., Boča R., Fuhr O., Rajadurai Ch, Ruben M. (2010). Supramolecular Lattice-Solvent
Control of Iron­(II) Spin Transition Parameters. CrystEngComm.

[5] Mukherjee S., Fedorov D. A., Varganov S. A. (2021). Modeling
Spin-Crossover
Dynamics. Annu. Rev. Phys. Chem..

[6] Kahn O., Martinez C. J. (1998). Spin-Transition Polymers: From Molecular Materials
toward Memory Devices. Science.

[7] Nemec I., Boča R., Herchel R., Trávníček Z., Gembický M., Linert W. (2009). Dinuclear Fe­(III) Complexes with
Spin Crossover. Monatshefte für Chemie
- Chemical Monthly.

[8] Lathion T., Fürstenberg A., Besnard C., Hauser A., Bousseksou A., Piguet C. (2020). Inorg. Chem..

[9] Wang M., Li Z.-Y., Ishikawa R., Yamashita M. (2021). Spin Crossover
and Valence Tautomerism Conductors. Coord. Chem.
Rev..

[10] Rat S., Piedrahita-Bello M., Salmon L., Molnár G., Demont P., Bousseksou A. (2018). Coupling Mechanical
and Electrical Properties in Spin Crossover Polymer Composites. Adv. Mater..

[11] Manrique-Juárez M. D., Rat S., Salmon L., Molnár G., Quintero C. M., Nicu L., Shepherd H. J., Bousseksou A. (2016). Switchable Molecule-Based Materials
for Micro- and Nanoscale Actuating Applications: Achievements and
Prospects. Coord. Chem. Rev..

[12] Šalitroš I., Madhu N. T., Boča R., Pavlik J., Ruben M. (2009). Room-Temperature
Spin-Transition Iron Compounds. Monatshefte
für Chemie - Chemical Monthly.

[13] Harding D. J., Harding P., Phonsri W. (2016). Spin Crossover
in Iron­(III) Complexes. Coord. Chem. Rev..

[14] Tweedle M.
F., Wilson L. J. (1976). Variable
Spin Iron­(III)
Chelates with Hexadentate Ligands Derived from Triethylenetetramine
and Various Salicylaldehydes. Synthesis, Characterization, and Solution
State Studies of a New ^2^T ↔ ^6^A Spin Equilibrium
System. J. Am. Chem. Soc..

[15] Dorbes S., Valade L., Real J. A., Faulmann C. (2004). [Fe­(Sal2-Trien)]­[Ni­(Dmit)­2]: Towards Switchable Spin
Crossover Molecular Conductors. Chem. Commun..

[16] Clemente-León M., Coronado E., López-Jordà M., Mínguez G., Soriano-Portillo A., Waerenborgh J. C. (2010). Multifunctional Magnetic Materials
Obtained by Insertion of a Spin-Crossover Fe^III^ Complex
into Bimetallic Oxalate-Based Ferromagnets. Chem. Eur. J..

[17] Floquet S., Muñoz M. C., Rivière E., Clément R., Audière J.-P., Boillot M.-L. (2004). Structural Effects on the Magnetic
Properties of Ferric
Complexes in Molecular Materials or a Lamellar CdPS_3_Host
Matrix. New J. Chem..

[18] Gandolfi C., Moitzi C., Schurtenberger P., Morgan G. G., Albrecht M. (2008). Improved Cooperativity of Spin-Labile
Iron­(III) Centers by Self-Assembly in Solution. J. Am. Chem. Soc..

[19] Martinho P. N., Lemma T., Gildea B., Picardi G., Müller-Bunz H., Forster R. J., Keyes T. E., Redmond G., Morgan G. G. (2012). Template
Assembly of Spin Crossover One-Dimensional Nanowires. Angew. Chem., Int. Ed..

[20] Vieira B. J.
C., da Gama V., Santos I. C., Pereira L. C. J., Bandeira N. A. G., Waerenborgh J. C. (2018). Magnetic
and Structural Correlations in [Fe­(Nsal_2_Trien)] Salts:
The Role of Cation–Anion Interactions in the Spin Crossover
Phenomenon. CrystEngComm.

[21] Halcrow M. A. (2011). Structure:function
Relationships in Molecular Spin-Crossover Complexes. Chem. Soc. Rev..

[22] Pogány L., Moncol J., Pavlik J., Šalitroš I. (2017). Series of
High Spin Mononuclear Iron­(Iii) Complexes with Schiff Base Ligands
Derived from 2-Hydroxybenzophenones. New J.
Chem..

[23] Percec V., Bae J.-Y., Zhao M., Hill D. H. (1995). Aryl Mesylates in
Metal-Catalyzed Homocoupling and Cross-Coupling Reactions. A Simple
and General Method for the Synthesis of 2,2’-Diaroyl-4,4’-Dihydroxybiphenyls. Journal of Organic Chemistry.

[24] Sheldrick G. M. (2015). SHELXT–
Integrated Space-Group and Crystal-Structure Determination. Acta Crystallographica Section A Foundations and Advances.

[25] Palatinus L., Chapuis G. (2007). *SUPERFLIP*– a Computer Program
for the Solution of Crystal Structures by Charge Flipping in Arbitrary
Dimensions. J. Appl. Crystallogr..

[26] Sheldrick G. M. (2015). Crystal
Structure Refinement WithSHELXL. Acta Crystallographica
Section C Structural Chemistry.

[27] Dolomanov O. V., Bourhis L. J., Gildea R. J., Howard J. A. K., Puschmann H. (2009). OLEX2: A Complete
Structure Solution, Refinement and Analysis Program. J. Appl. Crystallogr..

[28] Rigaku OxfordDiffraction CrysAlisPro (version 1.171.40.82a); Rigaku Oxford Diffraction Ltd.: 2020.

[29] Spackman P. R., Turner M. J., McKinnon J. J., Wolff S. K., Grimwood D. J., Jayatilaka D., Spackman M. A. (2021). *CrystalExplorer*:
A Program for Hirshfeld Surface Analysis, Visualization and Quantitative
Analysis of Molecular Crystals. J. Appl. Crystallogr..

[30] Hirshfeld F. L. (1977). Bonded-Atom Fragments for Describing
Molecular Charge Densities. Theoretica Chimica
Acta.

[31] Spackman M. A., McKinnon J. J. (2002). Fingerprinting Intermolecular
Interactions in Molecular Crystals. CrystEngComm.

[32] Boča, R. A Handbook of Magnetochemical Formulae; Elsevier: Amsterdam, 2012. ISBN: 978–0-12–416014–9.

[33] Neese F. (2022). Software Update: The ORCA Program
System—Version 5.0. *WIREs Computational Molecular*. Science.

[34] Becke A. D. (1988). Density-functional
exchange-energy approximation with correct asymptotic behavior. Phys. Rev. A.

[35] Weigend F. (2002). A Fully Direct RI-HF Algorithm: Implementation,
Optimised Auxiliary Basis Sets, Demonstration of Accuracy and Efficiency. Phys. Chem. Chem. Phys..

[36] Guo Y., Sivalingam K., Valeev E. F., Neese F. (2017). Explicitly Correlated
N-Electron Valence State Perturbation Theory (NEVPT2-F12). J. Chem. Phys..

[37] Handy N. C., Cohen A. J. (2001). Left-Right Correlation
Energy. Mol. Phys..

[38] Marenich A. V., Cramer C. J., Truhlar D. G. (2009). Universal Solvation
Model Based on
Solute Electron Density and on a Continuum Model of the Solvent Defined
by the Bulk Dielectric Constant and Atomic Surface Tensions. J. Phys. Chem. B.

[39] Neese F., Wennmohs F., Hansen A., Becker U. (2009). Efficient, Approximate and Parallel Hartree–Fock
and Hybrid DFT Calculations. A ‘Chain-of-Spheres’ Algorithm
for the Hartree–Fock Exchange. Chem.
Phys..

[40] Hanwell M. D., Curtis D. E., Lonie D. C., Vandermeersch T., Zurek E., Hutchison G. R. (2012). Avogadro:
An Advanced Semantic Chemical
Editor, Visualization, and Analysis Platform. J. Cheminform..

[41] Kragskow, J. Magnetism Tools. University of Manchester, Manchester, U.K. https://magnetism-tools.manchester.ac.uk/apps/tanabe_sugano_app (accessed Jul 10, 2025).

[42] Mazur M. (2006). A Dozen Useful
Tips on How to Minimise the Influence of Sources of Error in Quantitative
Electron Paramagnetic Resonance (EPR) Spectroscopy—a Review. Anal. Chim. Acta.

[43] Thiele, H. ; Etstling, J. ; Such, P. ; Hoefer, P. WINEPR, Bruker Analytic Gmb: Germany, 1992.

[44] Mazur M., Pogány L., Brachňaková B., Šalitroš I. (2020). A Variable-Temperature
Q- and X-Band EPR Study of Spin-Crossover Iron­(III) Schiff Base Complex. Chemical Papers.

[45] Weber, R. T. WINEPR SimFonia, EPR Division, Bruker Instruments Inc.: Billerica, USA,1995.

[46] Ozarowski, A. “Spin”, National High Magnetic Field Laboratory, Florida, USA. http://myweb.fsu.edu/aozarows/EPR/ (accessed Aug 11, 2014).

[47] bΘ = ∑_ *i*=1_ ^24^(|θ_ *i* _ – 60|); where θ_ *i* _are 24 angles measured on the projection of two triangular faces of the octahedron along with their common pseudo-threefold axis. Adapted from the ref 20.

[48] Alvarez S., Avnir D., Llunell M., Pinsky M. A. (2002). Continuous Symmetry Maps and Shape
Classification. New J. Chem..

[49] Maglic J. B., Lavendomme R. J. (2022). MoloVol: an easy-to-use program for
analyzing cavities,
volumes and surface areas of chemical structures. J. Appl. Crystallogr..

[50] Peng S., Gao Y., Zhang Z.-Y., Xu G.-Y., Zheng W.-J., Yang F.-L., Dai J.-W., Li Z.-Y. (2025). Spin Crossover
OFF/ON Triggered by
Ligand Chemical Doping in an Fe­(III) Solid Solution. Chin. J. Chem..

[51] Bader, R. F. W. Atoms in Molecules; Oxford University Press: 1994.

[53] Neese F. (2003). An Improvement
of the Resolution
of the Identity Approximation for the Formation of the Coulomb Matrix. J. Comput. Chem..

[54] Lee C., Yang W., Parr R. G. (1988). Development
of the Colle-Salvetti
correlation-energy formula into a functional of the electron density. Phys. Rev. B.

[55] Lu T., Chen F. (2012). Multiwfn: A multifunctional
wavefunction analyzer. J. Comput. Chem..

[56] Keith, T. A. AIMAll (Version 19.10.12); TK Gristmill Software: Overland Park KS, USA, 2019. https://aim.tkgristmill.com.

[57] Nemec I., Herchel R., Trávníček Z. (2015). The Relationship
between the Strength of Hydrogen Bonding and Spin Crossover Behaviour
in a Series of Iron­(Iii) Schiff Base Complexes. Dalton Transactions.

[58] Espinosa E., Alkorta I., Elguero J., Molins E. (2002). From Weak to Strong
Interactions: A Comprehensive Analysis of the Topological and Energetic
Properties of the Electron Density Distribution Involving X–H···F–Y
Systems. J. Chem. Phys..

[59] Espinosa E., Molins E., Lecomte C. (1998). Chem. Phys. Lett..

[60] Chilton N. F., Anderson R. P., Turner L. D., Soncini A., Murray K. S. (2013). PHI: A
Powerful New Program for the Analysis of Anisotropic Monomeric and
Exchange-Coupled Polynuclear *d*- and *f*-Block Complexes. J. Comput. Chem..

[61] Lang L., Atanasov M., Neese F. (2020). Improvement
of Ab Initio Ligand Field Theory by Means of Multistate Perturbation
Theory. J. Phys. Chem. A.

[62] Šagátová A., Kotrle K., Brachňaková B., Havlíček L., Nemec I., Herchel R., Hofbauerova M., Halahovets Y., Šiffalovič P., Čižmár E., Fellner O. F., Šalitroš I. (2024). Above room temperature
spin crossover in mononuclear iron­(II) complexes featuring pyridyl-benzimidazole
bidentate ligands adorned with aliphatic chains. Dalton Trans..

[63] Nicolazzi W., Bousseksou A. (2018). Thermodynamical
aspects of the spin crossover phenomenon. Compt.
Ren. Chim..

[64] Siig O. S., Kepp K. P. (2018). Iron­(II) and Iron­(III) Spin Crossover:
Toward an Optimal
Density Functional. J. Phys. Chem. A.

[65] Gaspar A. B., Real J. A. (2014). Spin Crossover in Soft Matter. Coord. Chem. Rev..

[66] Chernyshov D., Hostettler M., Törnroos K. W., Bürgi H.-B. (2007). Lattice-Solvent Coupling in the Spin-Crossover
Transition of a Fe­(II) Complex. Phys. Rev. B.

[67] Craze A. R., Bhadbhade M. M., Kepert C. J., Lindoy L. F., Marjo C. E., Li F. (2018). Solvent Effects
on the Spin Transition in a Series of Fe­(II) Dinuclear
Triple Helicate Compounds. Crystals.

[68] Nakaya M., Ohtani R., Lindoy L. F., Hayami S. (2021). Light-induced excited
spin state trapping in iron­(III) complexes. Inorg. Chem. Front..

[69] Hayami S., Gu Z-z, Shiro M., Einaga Y., Fujishima A., Sato O. (2000). First Observation of
Light-Induced Excited Spin State Trapping for an Iron­(III) Complex. J. Am. Chem. Soc..

[70] Shatruk M., Phan H., Chrisostomo B. A., Suleimenova A. (2015). Symmetry-Breaking
Structural Phase Transitions in Spin Crossover Complexes. Coord. Chem. Rev..

[71] Šalitroš I., Fuhr O., Eichhöfer A., Kruk R., Pavlik J., Dlháň Ĺ, Boča R., Ruben M. (2012). The interplay of iron­(II) spin transition
and polymorphism. Dalton Transactions.

