# Supplementary material for: Thermally and Light-Induced Spin-Crossover in Iron(III) Complexes with Benzophenone-Based Saltrien Ligands: Hysteresis, Two-Step Transitions, and the LIESST Effect
Source: Inorg Chem. 2025 Dec 29;65(1):593–608. doi: 10.1021/acs.inorgchem.5c04707 (PMC12801305; doi:10.1021/acs.inorgchem.5c04707)
Supplement: Supplementary file 1 [file ic5c04707_si_001.pdf]

Supplementary information for

Thermally and Light-Induced Spin Crossover in Iron(III) Complexes with Benzophenone-Based Saltrien Ligands: Hysteresis, Two-Step Transitions, and LIESST Effect

Lukáš Pogány,[a] Kamil Kotrle,[b] Ivan Nemec,[b] Ján Moncol,[a] Milan Mazúr,[c] and Ivan Šalitroš[a*]

a) Department of Inorganic Chemistry, Faculty of Chemical and Food Technology, Slovak University of Technology in Bratislava, Bratislava SK-81237, Slovakia, **e-mail:* ivan.salitros@stuba.sk
b) Department of Inorganic Chemistry, Faculty of Science, Palacký University, 17. listopadu 12, 771 46 Olomouc, Czech Republic
c) Department of Physical Chemistry, Faculty of Chemical and Food Technology, Slovak University of Technology in Bratislava, Bratislava SK-81237, Slovakia.

Contents

S1 Review of iron(III)-saltrien molecular compounds 2
S2 Experimental part 8
S3 Absorption spectroscopy and thermogravimetry investigation 9
S4 Supplementary structural investigation 12
S5 Crystal packing 16
S6 Hirshfeld surface analysis 19
S7 QT-AIM calculations 26
S8 Magnetic measurements and computational studies 29
S9 X-ray powder diffraction studies 35
S10 EPR spectroscopy 40
S11 References 41

S1 Review of iron(III)-saltrien molecular compounds

Table S 1 Iron(III)-saltrien compounds with their structural and magnetic parameters (color code: red - permanently HS, blue - SCO starts below RT, green - SCO starts above RT, black – a very incomplete and gradual SCO transition begins below room temperature and continues above it)

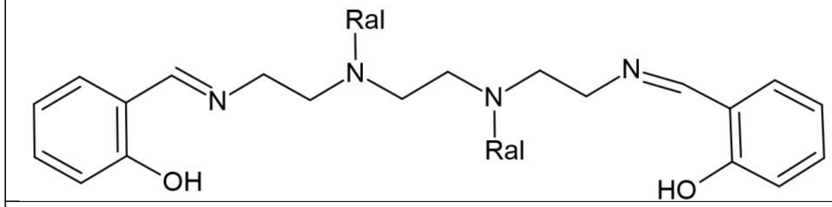

- **saltrien** (Ral=H)
- **C_6saltrien** (Ral=n-C_6H_{13})
- **C_{12}saltrien** (Ral=n-$C_{12}H_{23}$)

Compound, magnetic properties	avg. *d*(Fe–N^{am}) / Å	avg. *d*(Fe–N^{im}) / Å	*α* / °	*Σ* / °
**[Fe(saltrien)][Ni(dmit)$_2$]**[1]<br>abrupt SCO with hysteresis $T_{1/2}$=228/258 K	@293 K: 2.189	2.121	106.9	96.9
	@180 K: 2.003	1.930	72.9	40.3
**[Fe(saltrien)]BF$_4$**[2]<br>HS, no SCO	@293 K: 2.172	2.114	104.0	95.7
**[Fe(saltrien)]PF$_6$**[3]<br>(crystallised from CH_2Cl_4/Et_2O)<br>HS, no SCO	@150 K: 2.210(Fe1); 2.214(Fe2)	2.131(Fe1); 2.132(Fe2)	114.6(Fe1); 121.1(Fe2)	95.0(Fe1); 105.0(Fe2)
**[Fe(saltrien)]ClO$_4$**[3]<br>half-complete gradual SCO, $T_{1/2}$≈300K (estimated)	@105 K: 2.205(Fe1); 2.2023(Fe2)	2.109(Fe1); 1.949(Fe2)	76.6(Fe1); 73.6(Fe2)	96.0(Fe1); 46.3(Fe2)
**[Fe(saltrien)](TCNQ)$_2$·CH_3OH**[4]<br>LS, onset of SCO above 300 K	@100 K: 2.007	1.936	53.9	42.1
**[Fe(saltrien)](TCNQ)$_2$·CH_3CN**[4]<br>LS, onset of SCO above 300 K	@100 K: 2.006	1.931	77.2	48.5
**[Fe(C_6saltrien)]BF$_4$**[5]<br>very incomplete and gradual SCO, majority of iron(III) centers in permanent HS state	@100 K: 2.221(Fe1), 2.052(Fe2)	2.098(Fe1); 1.946(Fe2)	82.4(Fe1); 86.3(Fe2)	79.2(Fe1); 65.0(Fe2)
	@250 K: 2.231(Fe1), 2.102 (Fe2)	2.102(Fe1); 2.062(Fe2)	85.1(Fe1); 86.3(Fe2)	81.8 (Fe1); 67.2(Fe2)
**[Fe(C_{12}saltrien)](BF$_4$)$_{0.8}$Br$_{0.2}$**[6]<br>gradual SCO, $T_{1/2}$≈170 K (estimated)	@100 K: 2.076	1.956	89	64
	@293 K: 2.221	2.087	84	79

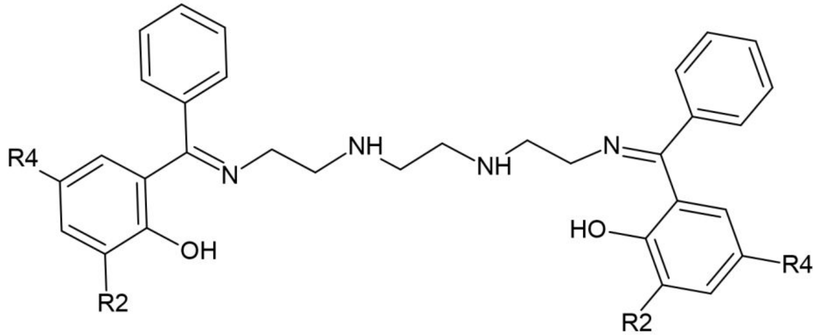

- **H_2L_1**: ($R_2 = R_4 = CH_3$)
- **H_2L_2** (R_2 = H, $R_4 = OCH_3$)

**Compound, magnetic properties**	**avg. *d*(Fe–N^{am}) / Å**	**avg. *d*(Fe–N^{im}) / Å**	***α* / °**	***Σ* /°**
**[Fe(L_1)]SeCN·CH_3CN (C1**, this work)<br>SCO above 225 K, accompanied by solvent loss above room temperature	@100 K: 2.000	1.961	69.3	46.3
**[Fe(L_1)]SeCN** (**C1d**, this work)<br>incomplete and gradual SCO, $T_{1/2}$ = 168/181 K (hysteresis)	@190 K: 2.173	2.144	106.4	112.1
**[Fe(L_1)]SeCN·2CH_3CN** (**C2**, this work)<br>SCO above 225 K, accompanied by solvent loss above room temperature	@100 K: 1.977	1.944	69.7	44.9
**[Fe(L_1)]BPh_4** (**C3**, this work)<br>LS, onset of SCO above 300 K	@100 K: 2.006	1.949	75.9	48.5
**[Fe(L_2)]BPh_4 (C4,** this work)<br>LS, onset of SCO above 300 K	@100 K: 1.972	1.945	80.0	47.1

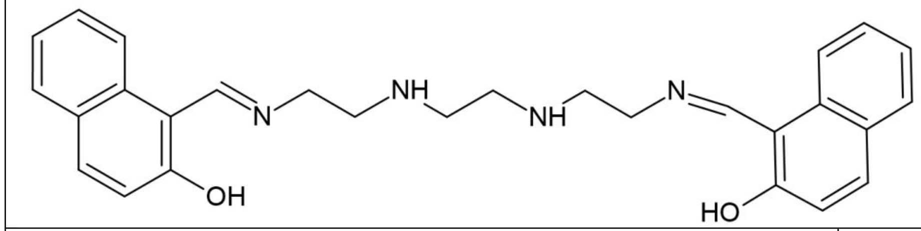	• **nsaltrien**			
**Compound, magnetic properties**	**avg. *d*(Fe–N^{am}) / Å**	**avg. *d*(Fe–N^{im}) / Å**	***α* / °**	***Σ* /°**
**[Fe(nsaltrien)]Cl·CH_3CH_2OH·H_2O**[7]<br>LS up to 300 K	@150 K: 1.996	1.918	76.5	45.3
**[Fe(nsaltrien)]ClO_4**[7]<br>HS, no SCO	@150 K: 2.178	2.103	98.0	97.4
**[Fe(nsaltrien)]BF_4**[3a,7]<br>HS, no SCO	@150 K: 2.179	2.097	96.3	96.5
**[Fe(nsaltrien)]BPh_4**[7]<br>gradual SCO above 225 K	@150 K: 1.997	1.928	83.4	35.4
**[Fe(nsaltrien)]PF_6**[7]<br>gradual SCO above 225 K	@150 K: 2.022	1.939	61.8	43.5
**[Fe(nsaltrien)]SCN**[7,8]<br>two-step SCO $T_{1/2}(1)$= 142 K (abrupt), $T_{1/2}(2)$= 250 K (gradual)	@294 K: 2.142	2.052	113	84.4
	@50 K: 2.011	1.911	114.6	61.1

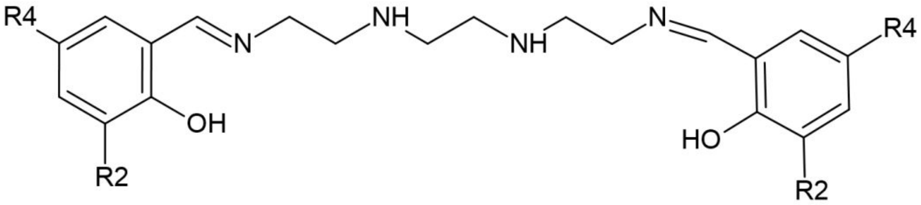	• ***4Fsaltrien*** (R2=H, R4=F)<br>• ***4Clsaltrien*** (R2=H, R4=Cl)<br>• ***4Brsaltrien*** (R2=H, R4=Br)<br>• ***4Isaltrien*** (R2=H, R4=I)<br>• ***2,4Cl$_2$saltrien***(R2=R4=Cl)			
**Compound, magnetic properties**	**avg. *d*(Fe–N^{am}) / Å**	**avg. *d*(Fe–N^{im}) / Å**	***α* / °**	***Σ* /°**
**[Fe(4Fsaltrien)]PF_6**[9]<br>incomplete, gradual and one-step SCO, $T_{1/2}$=177 K. more than 50% of material in permanent HS state	@100 K: 2.012	1.942	121.6	52.57
	@300 K: 2.198	2.099	124.3	87.3
**[Fe(4Fsaltrien)]ClO_4**[9]	@100 K: 2.045(Fe1), 2.105(Fe2)	1.965(Fe1), 2.199(Fe2)	112.6, 124.9	61.5, 111

half-complete, gradual and two-step SCO, $T_{1/2}(1)$= 114 K, $T_{1/2}(2)$= 170 K	@300 K: 2.177(Fe1), 2.216(Fe2)	2.086(Fe1), 2.127(Fe2)	111.7, 124.9	83.5, 116
**[Fe(4Fsaltrien)]I** [9]<br>half-complete, gradual and one-step SCO, $T_{1/2}$=227 K	@100 K: 2.034(Fe1), 2.214(Fe2)	1.987(Fe1), 2.158(Fe2)	108.8, 123.3	63.7, 128
	@300 K: 2.133(Fe1), 2.204(Fe2)	2.082(Fe1), 2.103(Fe2)	107.1, 122.5	76.2, 115
**[Fe(4Fsaltrien)]NO_3**[9]<br>HS, no SCO	@100 K: 2.164 (Fe1), 2.193(Fe2)	2.112 (Fe1), 2.148 (Fe2)	108.4, 117.9	87.5, 120
**[Fe(4Fsaltrien)]ReO_4** [10]<br>HS, no SCO	@100 K: 2.163	2.087	104.8	114.8
	@300 K: 2.201	2.106	104.5	115.4
**[Fe(4Clsaltrien)]ReO_4** [10]<br>two-step SCO $T_{1/2}(1)$= 209 K (abrupt), $T_{1/2}(2)$= 250 K (gradual). Compound undergoes thermally induced phase transition between three phases	@100 K: 2.024[a]	1.947	84.7	55.3
	@250 K: 2.066[a]	1.981	89.8	99.2
	@300 K: 2.126[a]	2.047	89.1	69.3
**[Fe(4Brsaltrien)]ReO_4** [10]<br>LS, onset of SCO above 350 K	@300 K: 2.106	1.933	69.6	49.6
**[Fe(4Isaltrien)]ReO_4** [10]<br>LS, onset of SCO above 350 K	@100 K: 2.009	1.944	?	45.1
**[Fe(2,4Cl_2saltrien)]PF_6**[11,3a]<br>gradual SCO, $T_{1/2}\approx$150 K (estimated)	@293 K: 2.183	2.09	89.2	89.0
**[Fe(2,4Cl_2saltrien)]BPh_4**[11]<br>gradual SCO, $T_{1/2}\approx$300 K (estimated)	@293 K: 2.105	2.029	88.0	56.6

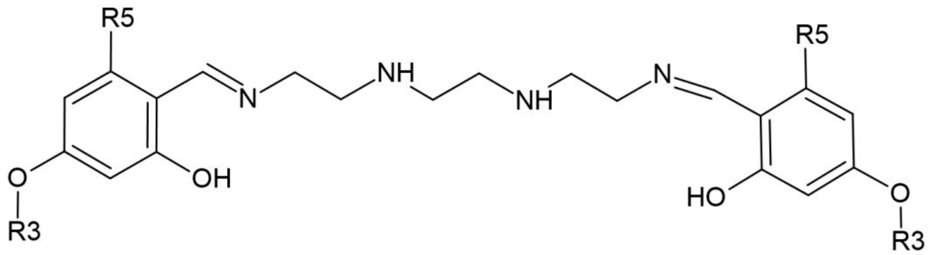

- ***3HOsaltrien*** (R3=R5=H)
- ***3MeOsaltrien*** (R3= Me, R5=H)
- ***3DIAOsaltrien*** (R3=4-(2-(5-methyl-2,4-dioxo-3,4-dihydropyrimidin-1(2H)-yl)ethoxy), R5=H)
- ***3,5(MeO)$_2$saltrien*** (R3=Me,R5=OMe)
- ***3C$_8$Osaltrien*** (R3=n-C_8H_{17}, R5=H)

**Compound, magnetic properties**	**avg. *d*(Fe–N^{am}) / Å**	**avg. *d*(Fe–N^{im}) / Å**	***α* / º**	***Σ* /º**
**[Fe(3HOsaltrien)]Cl**[12]<br>LS, onset of SCO above 350 K	@298 K: 2.011	1.922	62.3	45.4
**[Fe(3HOsaltrien)]Br**[12]	@298 K: 2.016	1.924	62.8	51.2

LS, onset of SCO above 350 K				
**[Fe(3HOsaltrien)]I**[12]<br>LS, onset of SCO above 300 K	@160 K: 2.011	1.932	62.7	50.2
**[Fe(3HOsaltrien)$_{-1H}$]** ($P2_1/c$)[13]<br>HS, no SCO	@293 K: 2.231	2.070	105.4	90.2
**[Fe(3HOsaltrien)$_{-1H}$]·2H_2O** ($P2_12_12_1$)[13]<br>HS at 300 K, magnetic properties not investigated	@293 K: 2.192	2.083	114.0	85.2
**[Fe(3HOsaltrien)][Co(himph)$_2$]·H_2O**[13]<br>HS, no SCO	@100 K: 2.188	2.101	98.7	97.2
**[Fe(3HOsaltrien)][Cr(himph)$_2$]·H_2O**[13]<br>HS, no SCO	@293 K: 2.185	2.099	100.2	97.9
**[Fe(3HOsaltrien)][Ag(CN)$_2$]**[13]<br>HS, no SCO	@100 K: 2.147	2.026	113.7	95.2
**[Fe(3MeOsaltrien)]PF_6·0.5H_2O**[11]<br>HS, no SCO	@293 K: 2.209	2.105	124.0	85.3
**[Fe(3MeOsaltrien)]ClO_4**[11]<br>LS, onset of SCO above 270 K	@293 K: 2.022	1.953	73.9	49.2
**[Fe(3DIAOsaltrien)]ClO_4·H_2O**[14]<br>abrupt SCO, $T_{1/2}$=343 K	@100 K: 1.988	1.921	106.0	45.6
**[Fe(3DIAOsaltrien)]ClO_4**[14]<br>HS, no SCO	@100 K: 2.179	2.097	121.7	92.8
**[Fe((3,5MeO)$_2$saltrien)]ClO_4**[15]<br>two-step SCO, estimated $T_{1/2}(1) \approx 90$ K (hysteresis), $T_{1/2}(2) \approx 200$ K (gradual). Compound undergoes thermally induced phase transition between two phases	@160 K: 2.002(Fe1); 2.189(Fe2)	1.924(Fe1), 2.144(Fe2)	107.6(Fe1), 115.7(Fe2)	53.8 (Fe1), 87.5 (Fe2)
	@200 K: 2.144	2.045	111.8	90.2
**[Fe(3C$_8$Osaltrien)]PF_6**[16]<br>SCO detected in solution, structure is HS at 173 K	@173 K: 2.186(Fe1), 2.208(Fe2)	2.109(Fe1), 2.102(Fe2)	122.81(Fe1), 116.25(Fe2)	95.7(Fe1), 97.2(Fe2)
**[Fe(3C$_8$Osaltrien)]PF_6**[17]<br>SCO detected in solution, structure is HS at 173 K	@173 K: 2.186(Fe1), 2.208(Fe2)	2.109(Fe1), 2.102(Fe2)	122.81(Fe1), 116.25(Fe2)	95.7(Fe1), 97.2(Fe2)

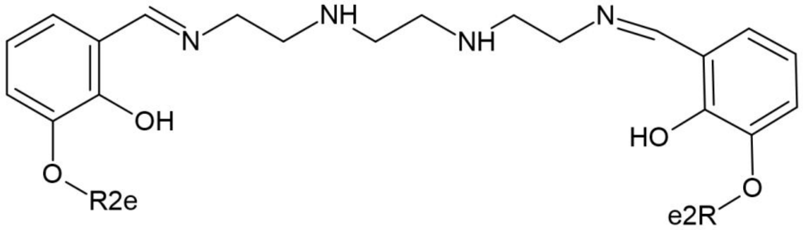	• **2HOsaltrien** (Reth=H)<br>• **2MeOsaltrien** (Reth=Me)			
**[Fe(2HOsaltrien)][Ni(dmit)$_2$]**[18]<br>HS, no SCO	@120 K: 2.172	2. 111	94.4	98.3
**[Fe(2MeOsaltrien)]NO$_3$·H$_2$O**[18]<br>LS, onset of SCO above 300 K	@120 K: 2.001	1.962	75.8	46.4
**[Fe(2MeOsaltrien)]I**[18]<br>LS, onset of SCO above 300 K	@100 K: 2.171	2.145	106.1	102.7
**[Fe(2MeOsaltrien)][Au(dmit)$_2$]**[19]<br>HS, no SCO	@120 K: 2.178	2.114	94.2	98.9
	@293 K: 2.180	2.113	95.3	97.3
**[Fe(2MeOsaltrien)][Au(ddtt)$_2$]**[19]<br>abrupt SCO, $T_{1/2}$=118 K	@120 K: 1.999	1.936	88.9	37.5
	@293K: 2.152	2.082	93.8	78.2
[20]**[Fe(2MeOsaltrien)][Fe(tdas)$_2$]·CH$_3$CN**[21]<br>gradual and incomplete SCO, $T_{1/2}$≈270 K (estimated)	@150 K: 2.013	1.938	79.7	97.1
	@297 K: 2.135	2.071	80.6	76.9
	@350 K: 2.168	2.104	82.0	86.6

himph=2-{(E)-[(2-hydroxyphenyl)imino]methyl}phenol, **dmit** = 4,5-dithiolato-1,3- dithiole-2-thione, **ddtt=** 5,6-dihydro-1,4-dithiin-2,3-dithiolate; **tdas**= 1,2,5-thiadiazole-3,4-dithiolate

S2 Experimental part

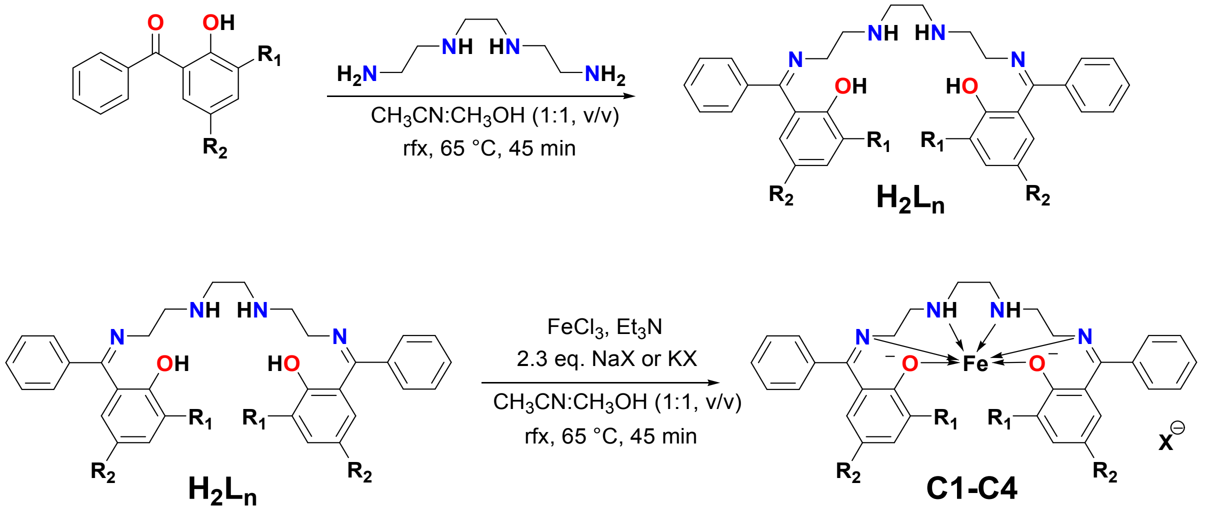

Scheme S1 Syntheses of ligands and complexes. **H_2L_1**: $R_1 = R_2 = CH_3$; **H_2L_2**: $R_1 = H$, $R_2 = OCH_3$. **C1**: $[Fe(L_1)]SeCN \cdot CH_3CN$; **C3**: $[Fe(L_1)]SCN \cdot 2CH_3CN$; **C2**: $[Fe(L_1)]BPh_4$; **C4**: $[Fe(L_2)]SeCN \cdot CH_3CN$.

S3 Absorption spectroscopy and thermogravimetry investigation

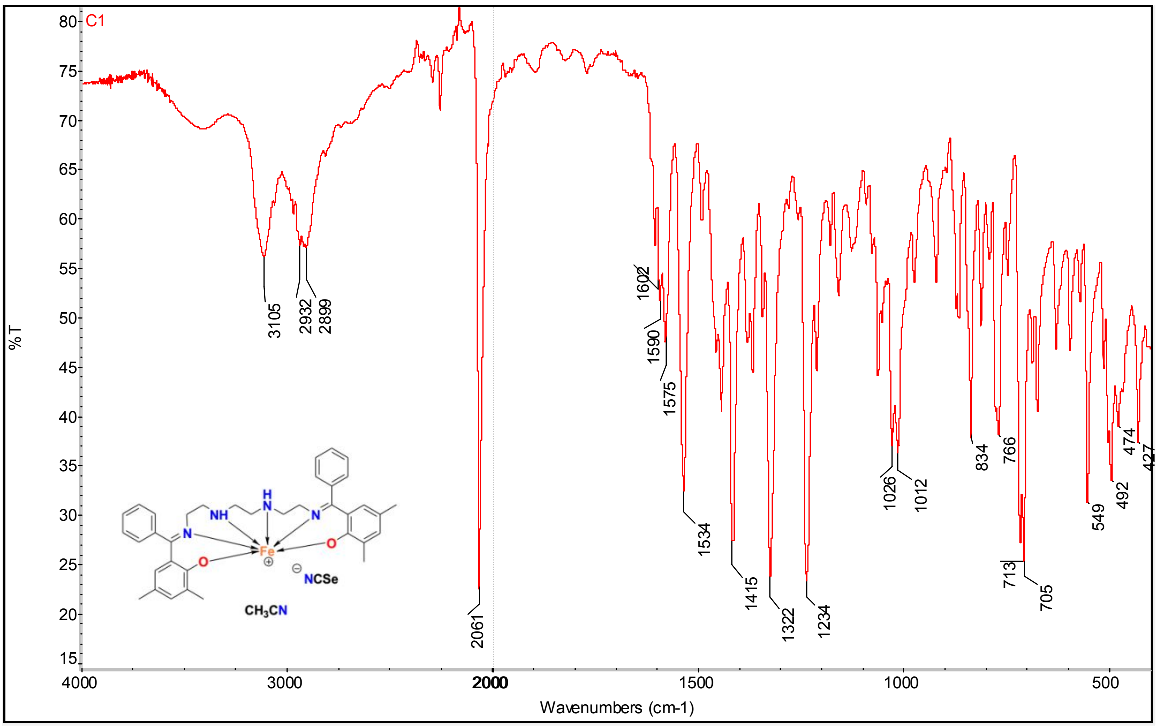

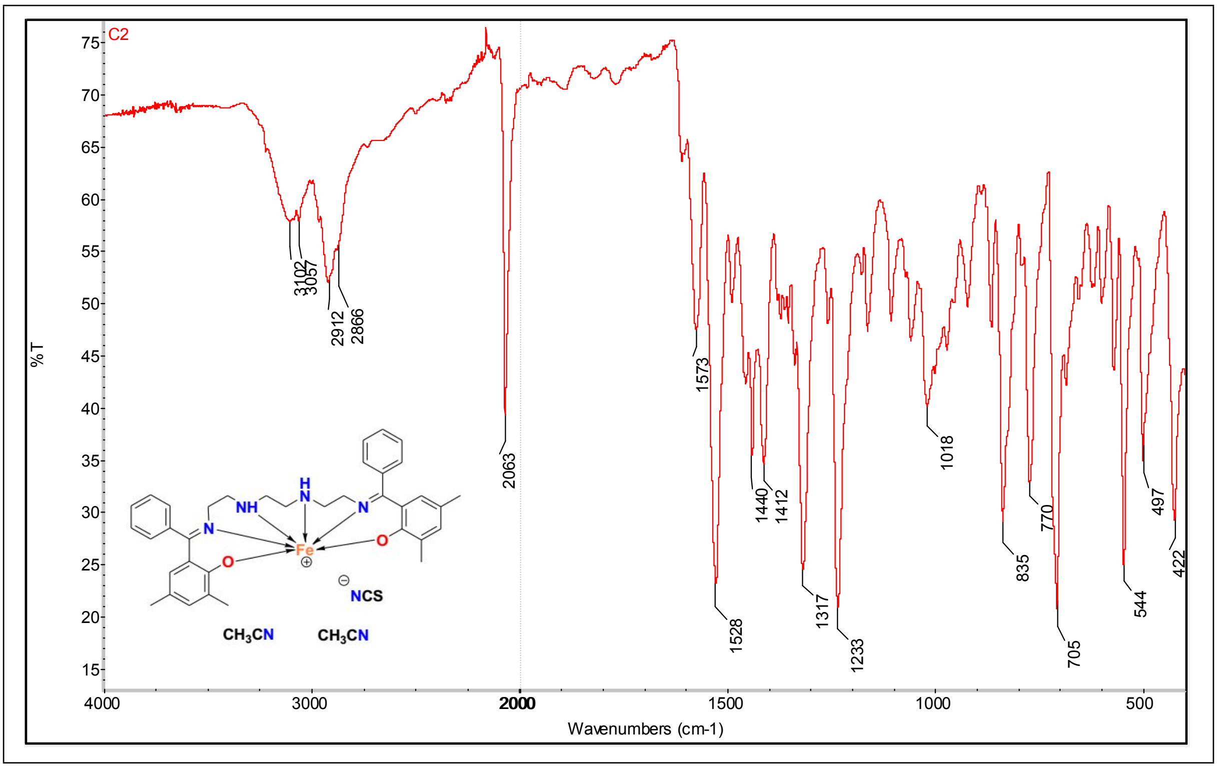

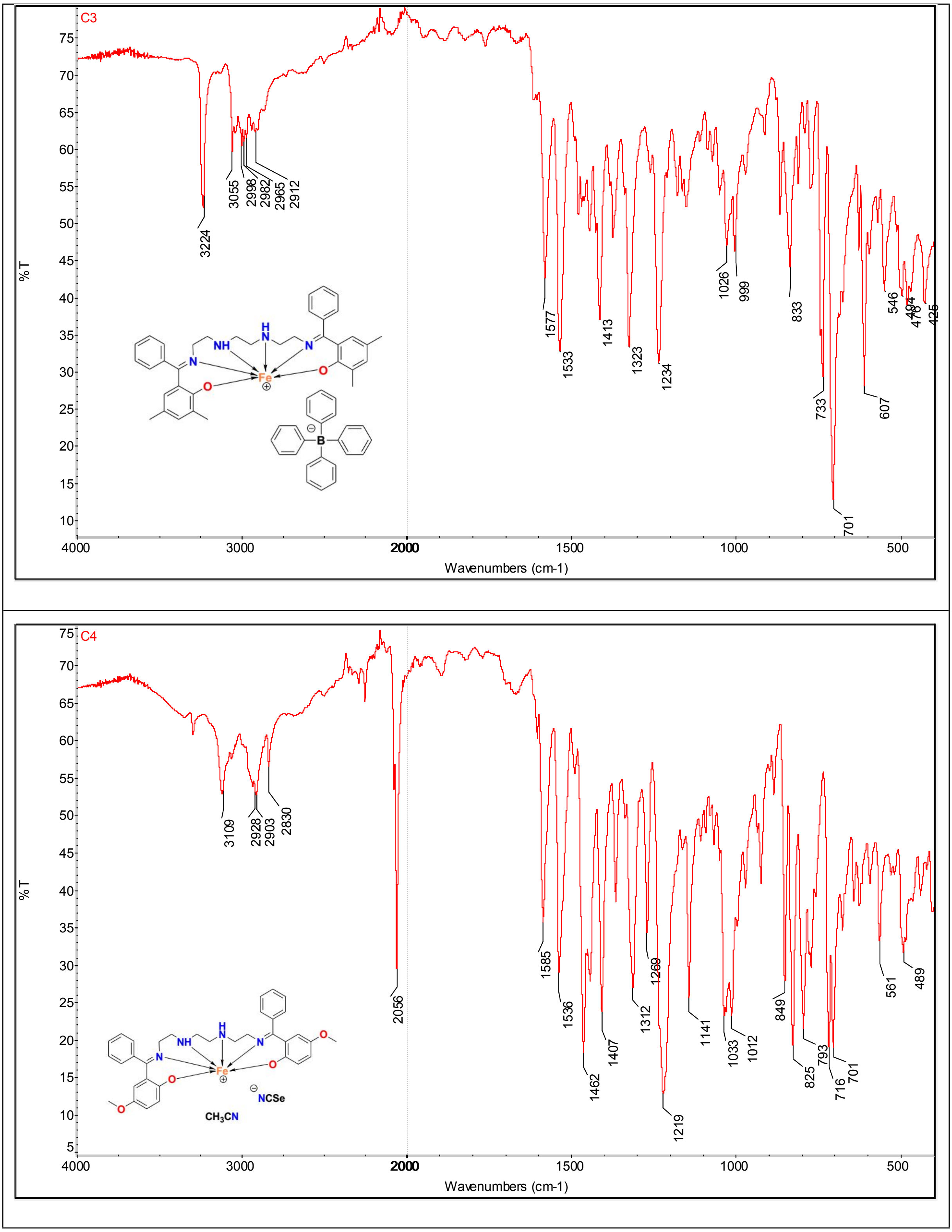

Figure S 1 FT-IR spectra of reported compounds

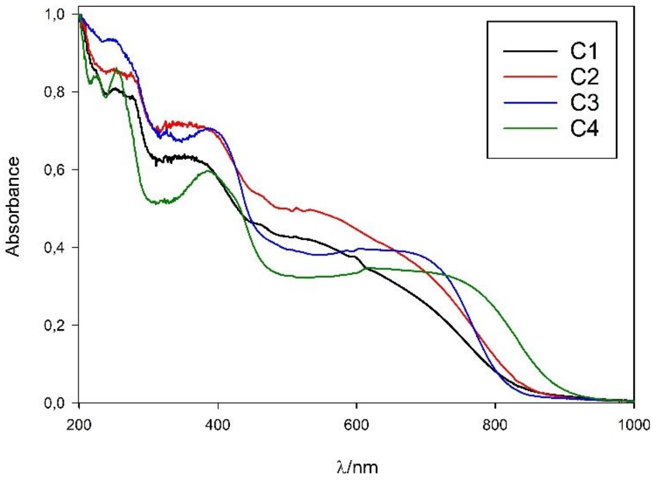

Figure S 2 UV-VIS spectra of reported compounds

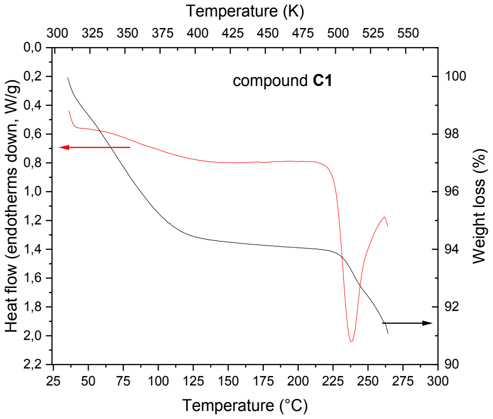

a)

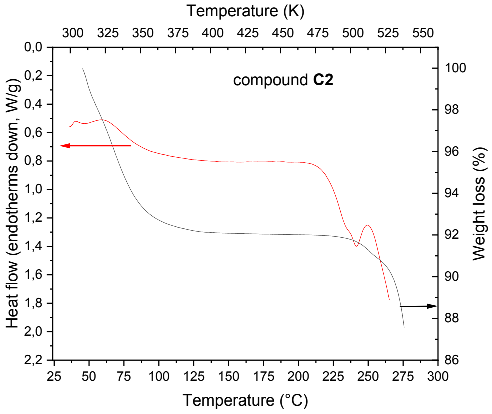

b)

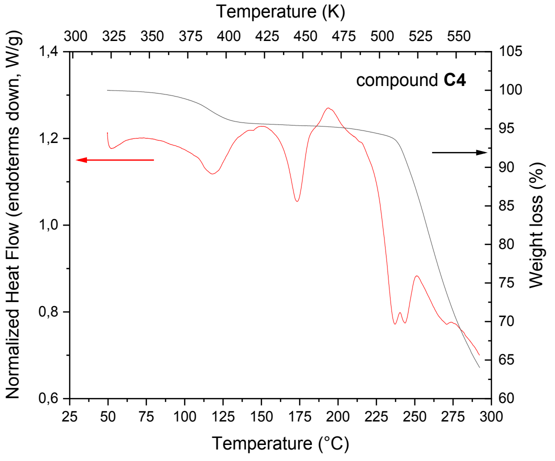

c)

Figure S 3 Thermogravimetric analysis for compounds **C1** (***a***), **C2** (***b***) and **C4** (***c***)

S4 Supplementary structural investigation

Table S 2 Crystallographic data for the reported compounds

**Compound**	**C1**	**C1d**	**C2**	**C3**	**C4**
**Empirical formula**	$C_{39}H_{43}FeN_6O_2Se$	$C_{37}H_{40}FeN_5O_2Se$	$C_{41}H_{46}FeN_7O_2S$	$C_{60}H_{60}BFeN_4O_2$	$C_{37}H_{39}FeN_6O_4Se$
**Formula weight**	755.01	721.55	756.76	935.78	766.55
**Temperature (K)**	100.0	190	100	100	100
**Wavelength (Å)**	1.54186	1.54186	1.54186	1.54186	1.54186
**Crystal system**	monoclinic	monoclinic	monoclinic	orthorhombic	monoclinic
**Space group**	$P2_1/n$	$P2_1/n$	$P2_1/n$	Pcca	$P2_1/n$
a **(Å)**	11.7669(2)	11.7718(7)	11.7551(7)	20.4010(4)	10.3475(3)
b **(Å)**	25.6378(5)	25.2864(8)	25.6196(16)	15.6235(3)	31.2860(5)
c **(Å)**	12.6846(2)	13.0727(6)	12.6818(7)	16.3477(4)	10.7223(3)
α **(°)**	90	90	90	90	90
β **(°)**	95.840(1)	114.367(6)	95.833(5)	90	95.115(2)
γ **(°)**	90	90	90	90	90
**V ($Å^3$)**	3806.8(1)	3544.7(3)	3799.5(4)	5210.58(19)	3457.33(15)
**Z, ρ_{calc} g.cm^{-3}**	4, 1.316	4, 1.352	4, 1.323	4, 1.193	4, 1.473
**μ (mm^{-1})**	4.582	4.889	4.054	2.670	5.103
**F (000)**	1562.0	1492	1596.0	1980.0	1580.0
**Crystal size (mm)**	0.18 × 0.05 × 0.04	0.24 × 0.2 × 0.1	0.48 × 0.05 × 0.04	0.16 × 0.15 × 0.14	0.32 × 0.26 × 0.05
**2θ Ranges (°)**	6.896 to 143.964	3.4750 to 68.1450	6.90 to 143.234	5.656 to 143.138	5.65 to 142.578
**Final R indices [I > 2σ(I)]**	$R_1 = 0.0831$	$R_1 = 0.1055$	$R_1 = 0.0395$	$R_1 = 0.0736$	$R_1 = 0.0429$
	$wR_2 = 0.2061$	$wR_2 = 0.2584$	$wR_2 = 0.0937$	$wR_2 = 0.2157$	$wR_2 = 0.0961$
**R indices (all data)**	$R_1 = 0.1497$	$R_1 = 0.1474$	$R_1 = 0.0537$	$R_1 = 0.0884$	$R_1 = 0.0597$
	$wR_2 = 0.2193$	$wR_2 = 0.2861$	$wR_2 = 0.0969$	$wR_2 = 0.2257$	$wR_2 = 0.0995$
**GoF**	0.911	1.080	1.009	1.085	1.005
**CCDC number**	1943432	2474399	1943433	1943434	1943435

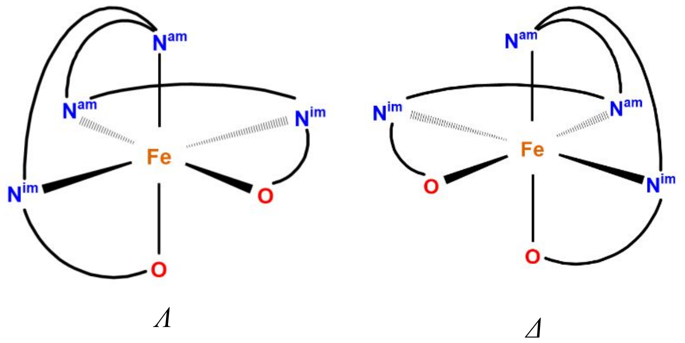

Figure S 4 *Λ* and *Δ* isomerism in iron(III)-*saltrien* octahedral complexes with *cis*-O_2/*trans*-N_2^{im} *mer*-{N^{im},N^{am},O}

Table S 3 Bond distances and distortion parameters for coordination polyhedra of **C1-C4**

Bond lengths	**C1**	**C1d**	**C2**	**C3**	**C4**
Fe1-O1	1.868(6)	1.875(6)	1.8522(15)	1.857(2)	1.866(2)
Fe1-O2	1.832(5)	1.887(5)	1.8588(16)	&	1.866(2)
Fe1-N1^{im}	1.962(6)	2.16(1)	1.9471(18)	1.949(3)	1.943(2)
Fe1-N2^{am}	2.010(7)	2.16(1)	2.0072(19)	2.006(3)	2.002(2)
Fe1-N3^{am}	1.989(6)	2.186(7)	2.0040(19)	&	2.009(2)
Fe1-N4^{im}	1.959(6)	2.127(9)	1.9416(17)	&	1.946(2)

& In **C3**, the atom Fe1 lies in special position on 2-fold axis, O2=O1, N4=N1 and N3=N2.

Table S 4 Distortion parameters and symmetry measure analysis* for **C1-C4** and **C1d**.

	α / °	Σ / °	Θ / °	D_{6h}	C_{5v}	O_h	D_{3h}	C_{5v}-J
**C1**	69.3	46.3	84.3	30.000	27.116	0.360	13.681	30.390
**C1d**	106.4	112.1	294.2	31.666	18.439	3.235	8.853	21.951
**C2**	69.7	44.9	85.0	29.953	26.915	0.370	13.678	30.081
**C3**	75.9	48.5	91.6	30.418	27.172	0.379	13.263	30.479
**C4**	80.0	47.1	79.8	30.367	27.054	0.331	13.839	30.446

* α, Σ,Θ – see main text for explanation, D_{6h}-hexagon, C_{5v}-pentagonal pyramid, O_h-octahedron, D_{3h}-trigonal prism, C_{5v}-J - Johnson pentagonal pyramid.

Table S 5 Angles and structural parameters of reported compounds

Bond angles	**C1**	**C1d**	**C2**	**C3**	**C4**
N2-Fe1-N3	84.1(3)	78.2(3)	84.75(7)	84.48(17)&	84.09(9)
N2-Fe1-N1	85.5(3)	76.6(3)	84.87(7)	85.12(12)&	85.98(9)
N2-Fe1-O2	90.9(3)	86.5(3)	89.63(7)	89.16(11)&	88.60(9)
N2-Fe1-N4	95.9(3)	109.3(3)	96.36(7)	95.61(12)&	95.67(9)
N4-Fe1-N3	85.3(3)	78.0(3)	85.45(7)	85.12(12)&	84.75(9)
O2-Fe1-N4	92.1(3)	85.6(3)	92.32(7)	93.01(10)&	94.24(9)
O2-Fe1-N1	87.9(3)	101.5(3)	87.51(7)	86.35(11)&	87.79(9)
N1-Fe1-N3	94.9(3)	97.3(3)	94.85(7)	95.60(12)&	93.45(9)
N3-Fe1-O1	90.6(2)	96.3(3)	90.34(7)	89.16(11)&	90.70(9)
N1-Fe1-O1	93.5(3)	83.8(3)	94.03(7)	93.01(10)&	92.90(9)
O1-Fe1-O2	94.5(2)	105.7(3)	95.34(7)	97.30(15)&	96.65(9)
O1-Fe1-N4	85.1(3)	89.3(3)	84.76(7)	86.34(11)&	85.28(9)

& In **C3**, the atom Fe1 lies in special position on 2-fold axis, O2=O1, N4=N1 and N3=N2.

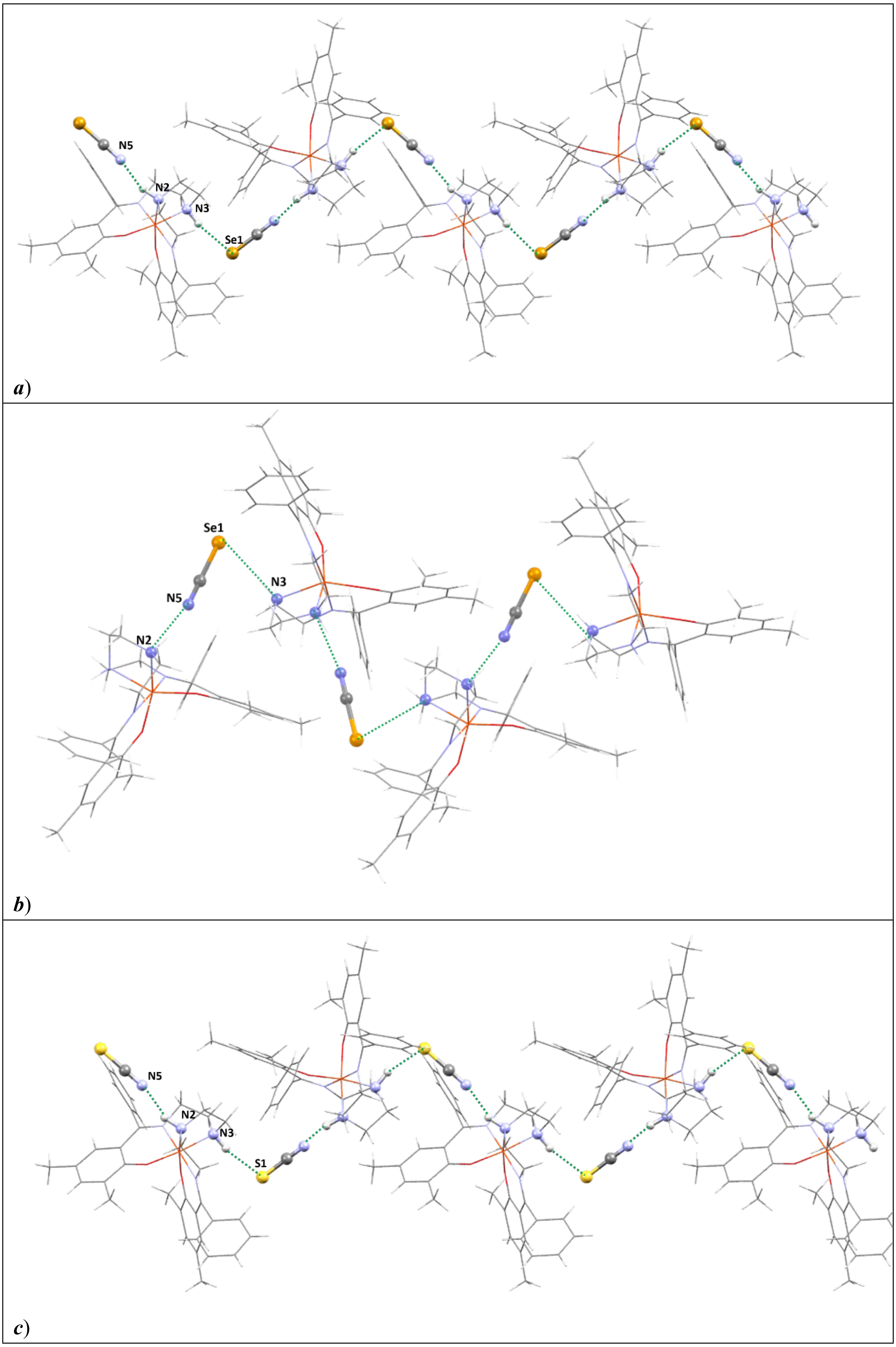
N5
N2
N3
Se1
a)
Se1
N3
N5
N2
b)
N5
N2
N3
S1
c)

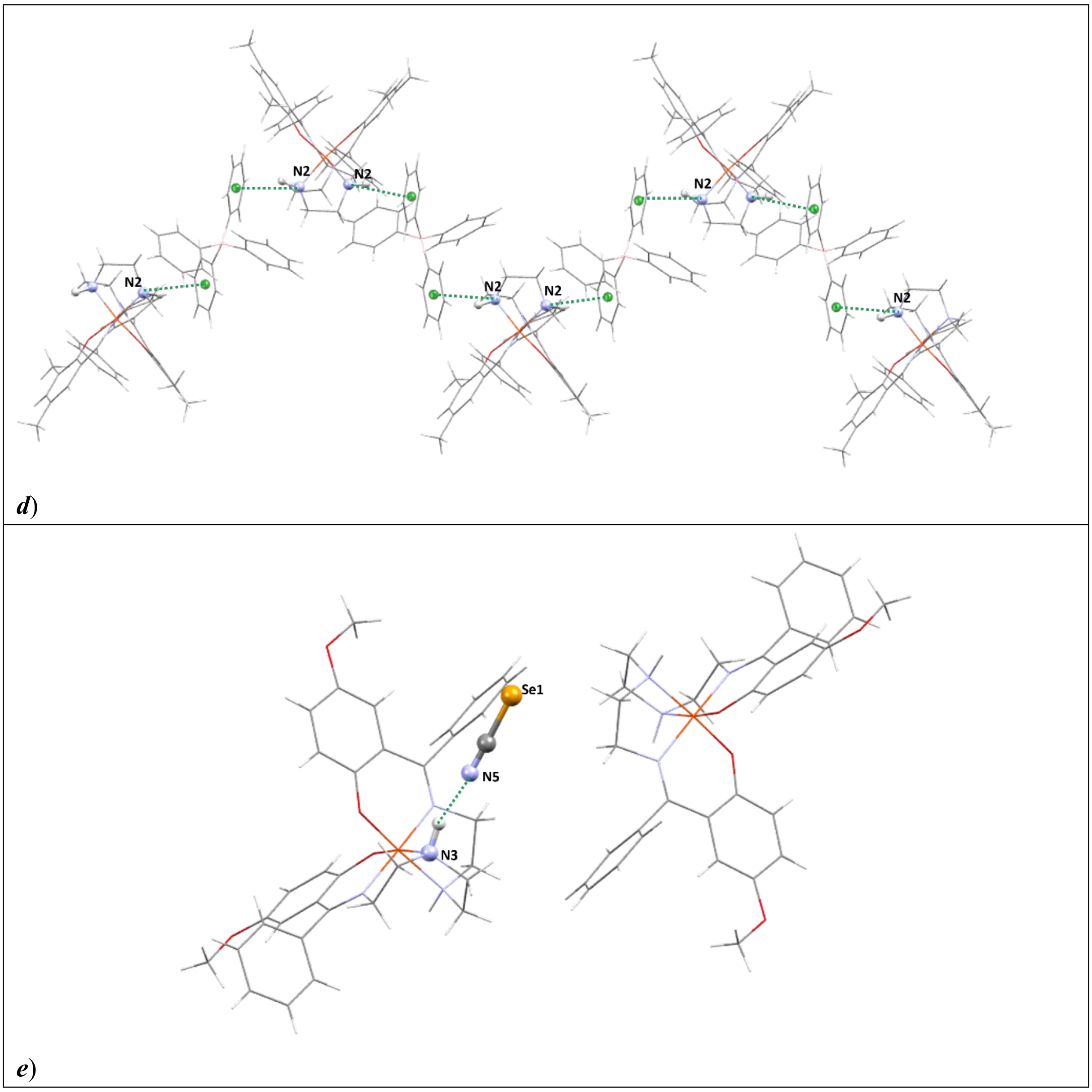

Figure S 5 ***a***) Visualization of H-bonds and short non-covalent contacts in crystal structure of **C1** (along *a-b* plane): N5□□□H2-N2=2.082(12) Å; N5□□□N2=3.008(14) Å; Se1□□□H3-N3=2.4246(12) Å; Se1□□□N3=3.397(7) Å; <(N5□□□H2-N2)=153.1(5)°; <(Se1□□□H3-N3)=163.7(4)°. ***b***) H-bonds and short contacts in crystal structure of **C1d** (along *a-c* plane): N5□□□H2-N2=1.951(11) Å; N5□□□N2=2.906(14) Å; Se1□□□H3-N3=2.4715(10) Å; Se1□□□N3=3.445(7) Å; <(N5□□□H2-N2)=158.6(6)°; <(Se1□□□H3-N3)=164.6(5)°. ***c***) H-bonds and short contacts in crystal structure of **C2** (along *a-b* plane): N5□□□H2-N2=2.058(2) Å; N5□□□N2=2.985(3) Å; S1□□□H3-N3=2.3700(7) Å; S1□□□ N3=3.3262(19) Å; <(N5□□□H2-N2)=153.27(12)°; <(S1□□□H3-N3)=159.75(10)°. ***d***) Short non-covalent contacts in crystal structure of **C3** (along *a-c* plane): centroid C19-C20-C21-C22-C23-C24□□□N2=3.271(4) Å. ***e***) H-bonds and short contacts in crystal structure of **C4**: N5□□□H3-N3=1.934(3) Å; N5□□□N3=2.914(4) Å; <(N5□□□H3-N3)=166.16(16)°. Color code: C- grey; H-white; N-blue; O-red; Fe-orange; Cl-green; Se-mustard yellow, S-pale yellow; B-pink, centroids of benzene rings – green, H-bonds and short contacts are visualized by green dashed line. Lattice solvent molecules are omitted for clarity.

S5 Crystal packing

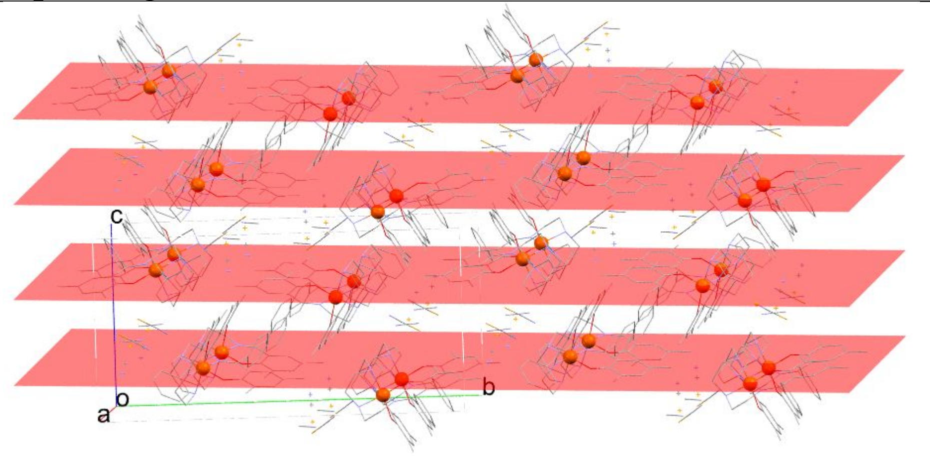

a) C1

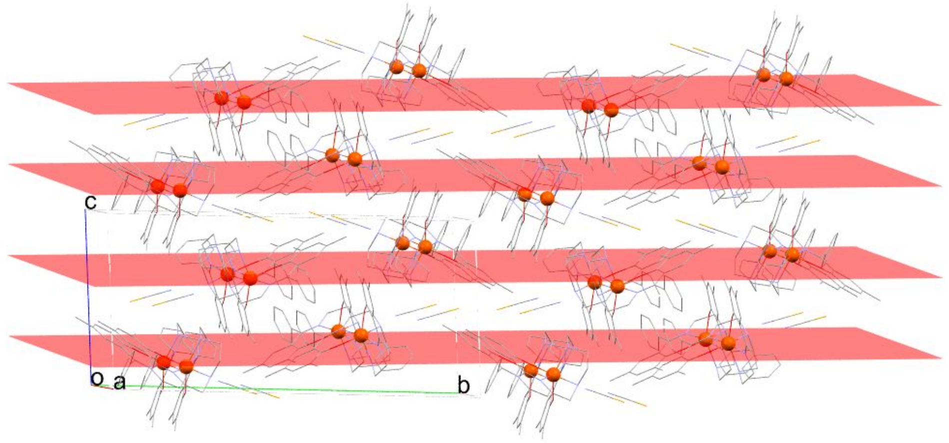

b) C1d

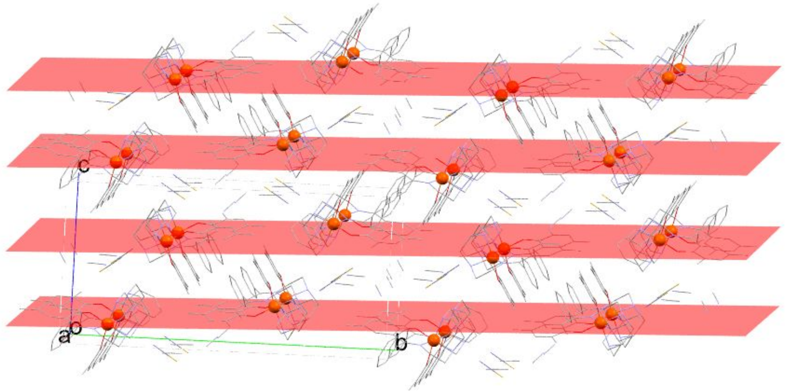

c) C2

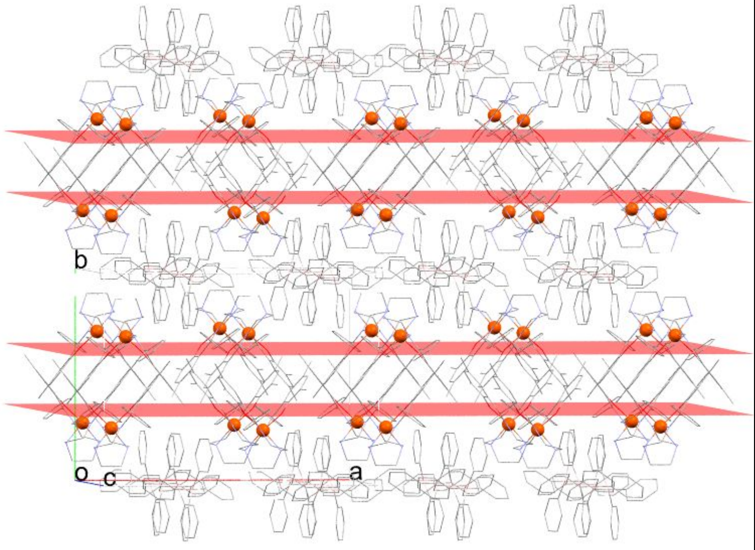

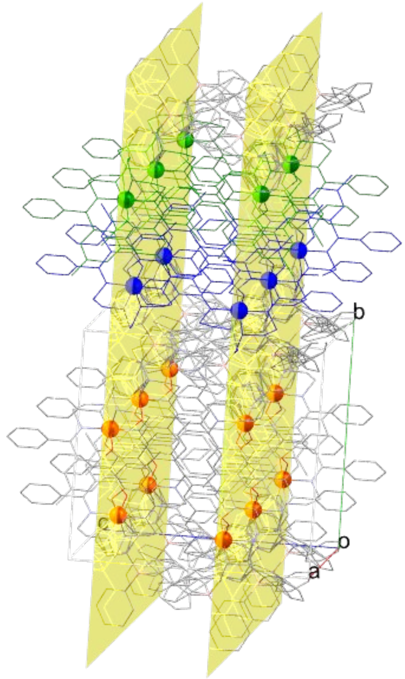

d) C3

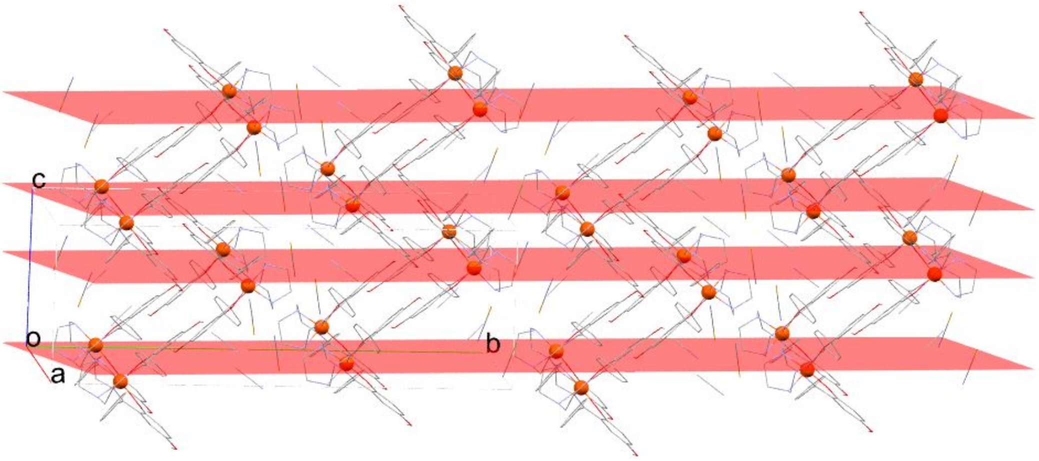

e) C4

Figure S 6 Packing arrangement of molecules in the reported crystal structures.

Table S 6 Calculated volumes for the reported crystal structures. Volumes (Å^3 per unit cell) and corresponding unit cell fractions (in parentheses) were determined by MoloVol[22] using a two-probe method (probe radii = 0.8, 1.2 Å; grid resolution = 0.3 Å).

	**C1**	**C1**$^{\&}$	**C1d**	**C2**	**C2**$^{\$}$	**C3**	**C3**$^{\&}$	**C4**
**Van der Waals volume**	2620.31 (0.69)	2567.89 (0.67)	2396.37 (0.68)	2758.81 (0.73)	2571.06 (0.68)	3735.20 (0.72)	3456.29 (0.66)	2500.51 (0.72)
**Probe excluded void volume**	771.53 (0.20)	829.19 (0.21)	783.03 (0.22)	928.64 (0.24)	806.99 (0.21)	867.20 (0.17)	893.67 (0.17)	753.00 (0.22)
**Molecular volume***	3391.84 (0.89)	3397.09 (0.89)	3179.40 (0.90)	3687.45 (0.97)	3378.04 (0.88)	4602.40 (0.88)	4349.96 (0.83)	3253.51 (0.94)
**Small probe occupied volume**	142.02 (0.04)	112.55 (0.03)	208.71 (0.06)	112.04 (0.03)	138.33 (0.04)	348.55 (0.07)	330.08 (0.06)	203.82 (0.06)
**Large probe occupied volume**	272.94 (0.07)	297.16 (0.08)	156.56 (0.04)	0	283.12 (0.07)	259.64 (0.05)	530.55 (0.10)	0
**Volume of voids and pores****	1186.49 (0.31)	1238.90 (0.33)	1148.31 (0.32)	1040.68 (0.27)	1228.43 (0.32)	1475.39 (0.28)	3456.29 (0.34)	956.82 (0.28)

$^{\&}$calculation performed on structure without disorders; $^{\$}$calculation performed on structure **C2** without disorders and with one removed molecule of CH_3CN; *summation of van der Waals volume and probe excluded void volume; **summation of probe excluded void volume, small probe occupied volume and large probe occupied volume.

S6 Hirshfeld surface analysis

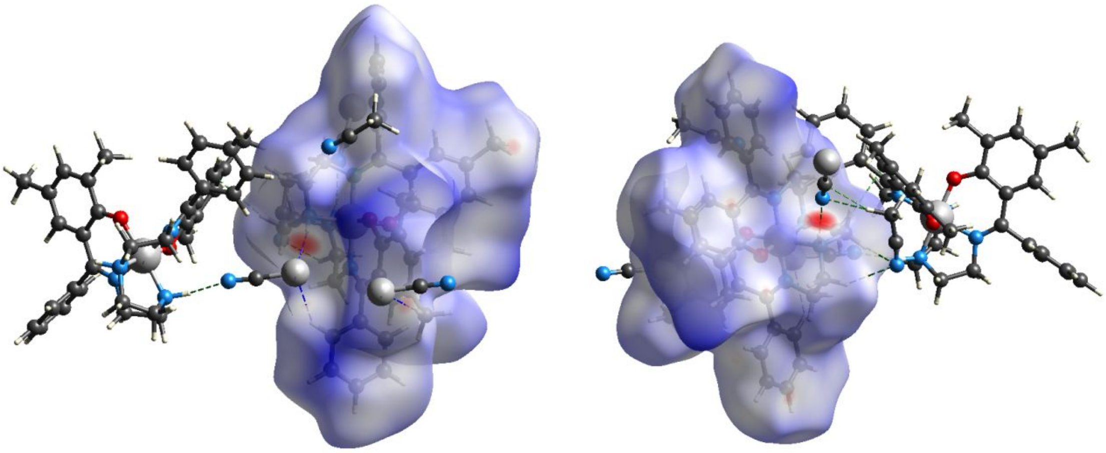

Figure S 7 Hirshfeld surface of **C1** mapped over d_{norm}. The N–H···N, C–H···N, N–H···Se and C–H···Se hydrogen bonds are indicated by red dashed lines.

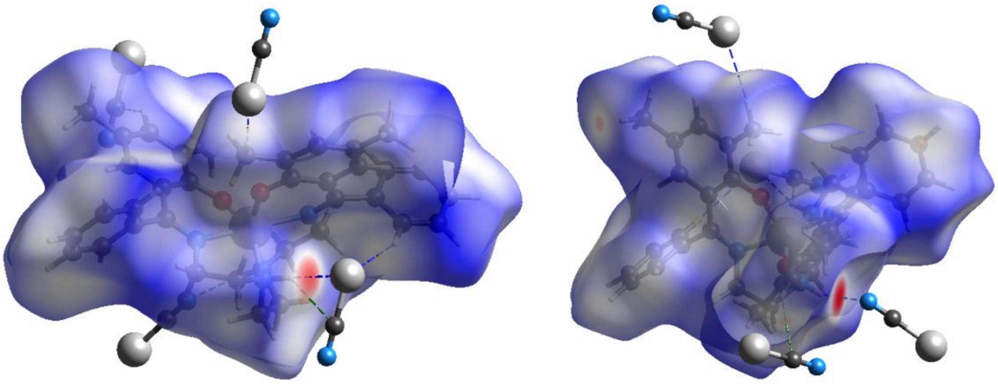

Figure S 8 Hirshfeld surface of **C1d** mapped over d_{norm}. The N–H···N, N–H···Se and C–H···Se hydrogen bonds are indicated by red dashed lines.

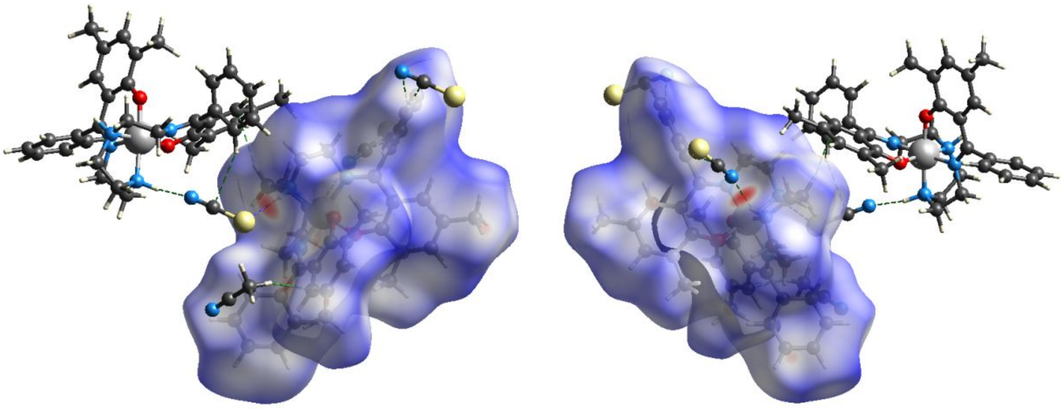

Figure S 9 Hirshfeld surface of **C2** mapped over d_{norm}. The N–H···N, C–H···N, C–H···π and C–H···S hydrogen bonds are indicated by red dashed lines.

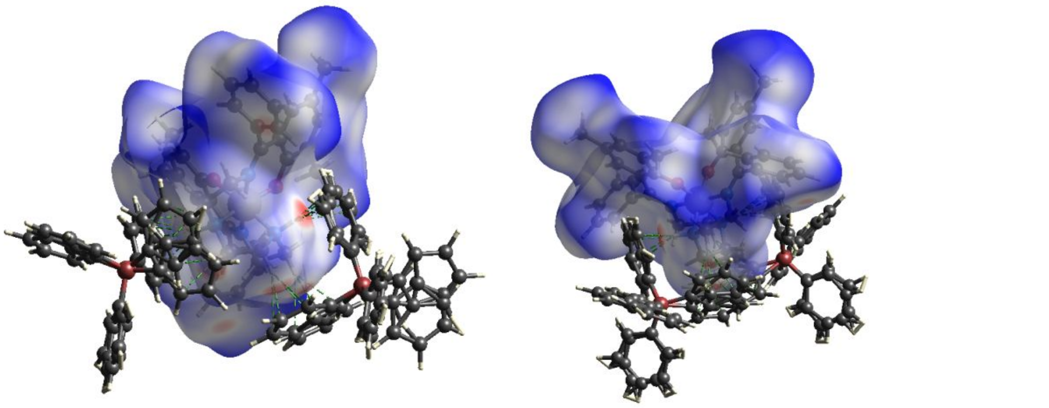

Figure S 10 Hirshfeld surface of **C3** mapped over d_{norm}. The N–H···π and C–H···π hydrogen bonds are indicated by red dashed lines.

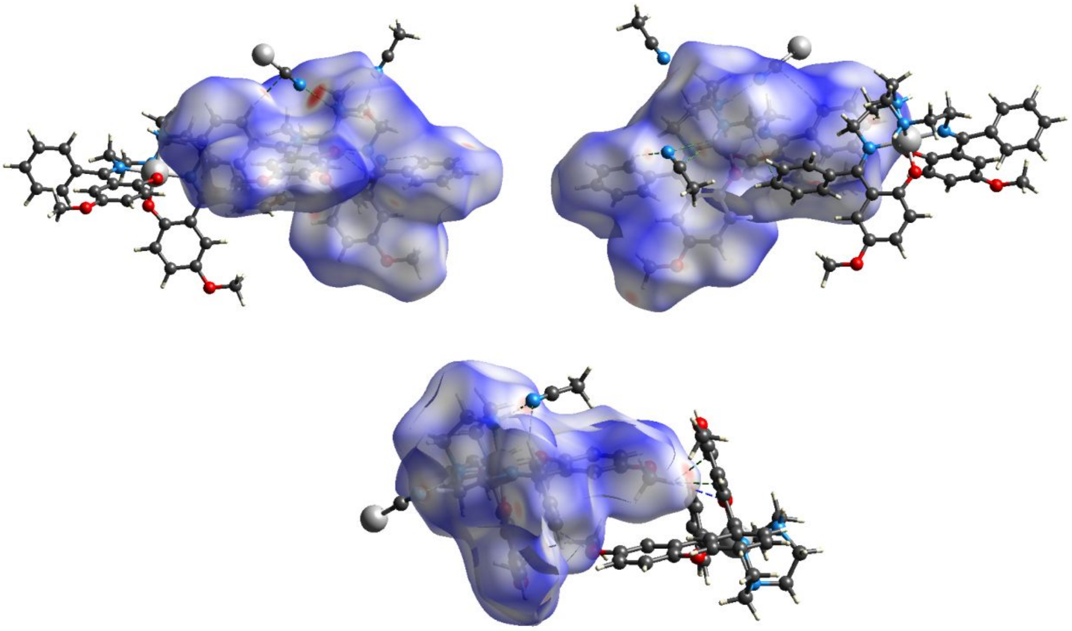

Figure S 11 Hirshfeld surface of **C4** mapped over d_{norm}. The N–H···N, C–H···N, C–H···O and C–H···π hydrogen bonds are indicated by red dashed lines.

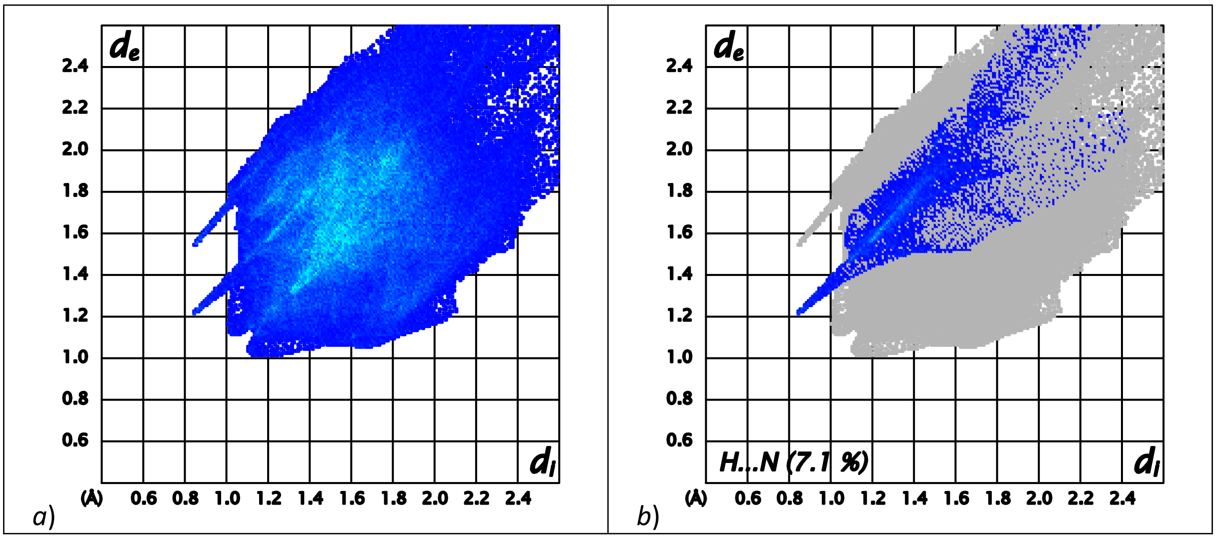

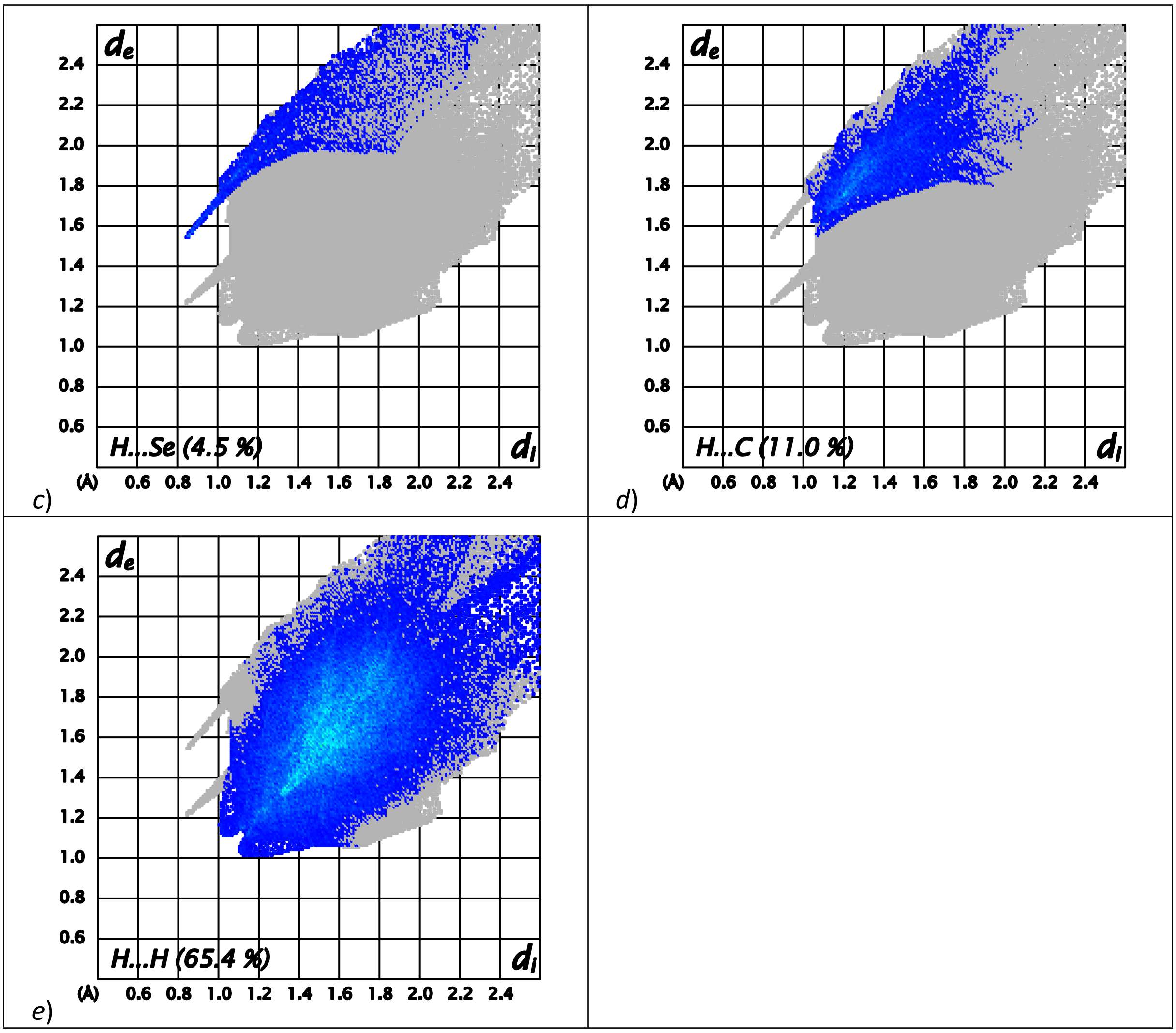

Figure S 12 The full two-dimensional fingerprint plots of **C1**, showing (*a*) all, (*b*) H···N, (*c*) H···Se, (*d*) H···C and (*e*) H···H interactions.

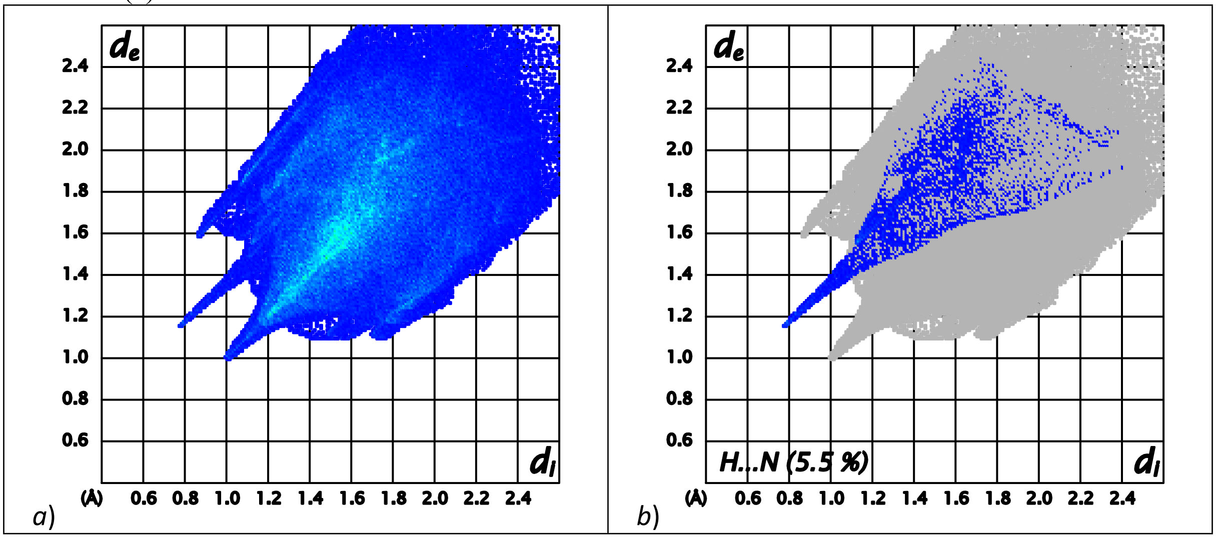

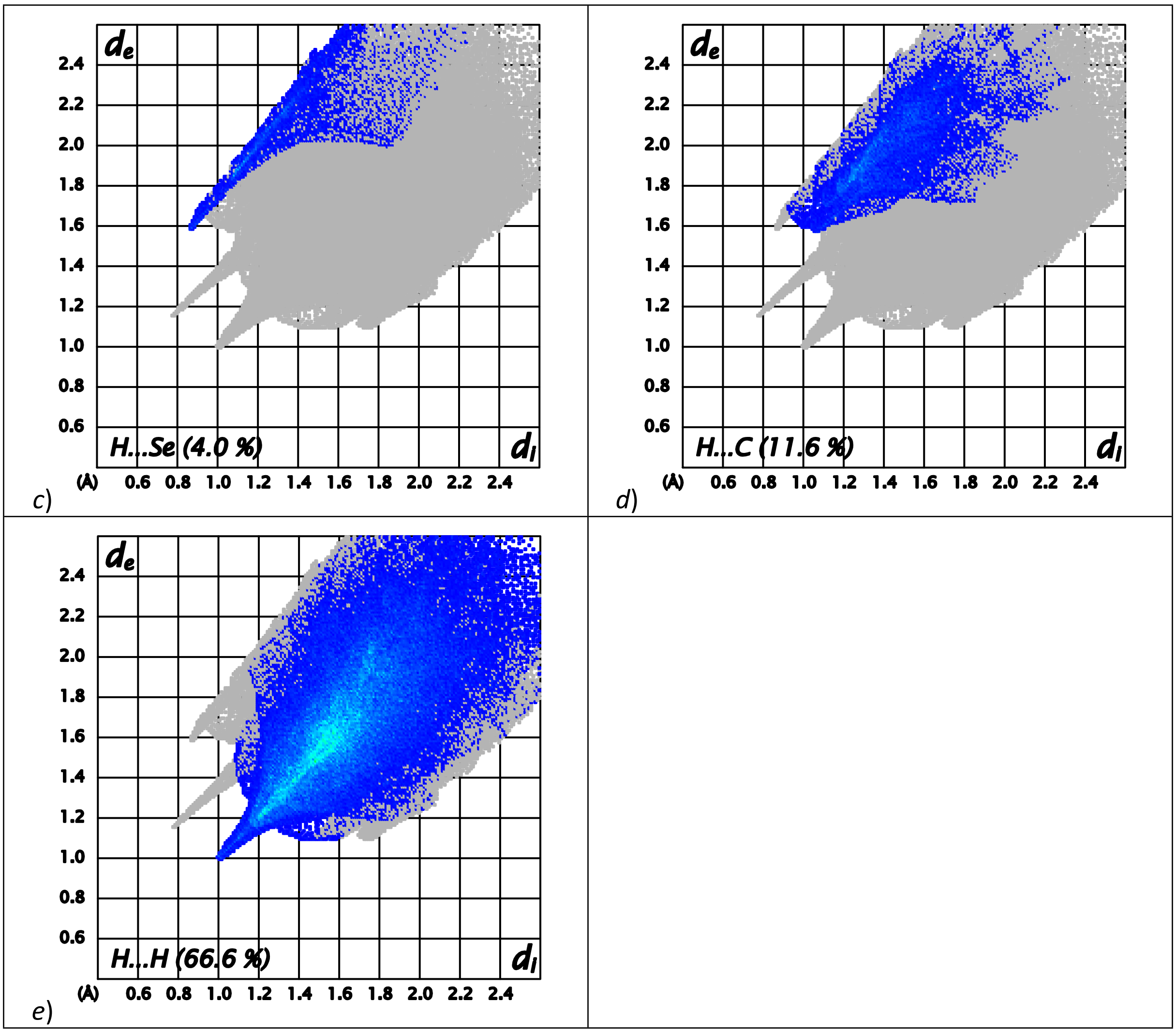

Figure S 13 The full two-dimensional fingerprint plots of **C1d**, showing (*a*) all, (*b*) H···N, (*c*) H···Se, (*d*) H···C and (*e*) H···H interactions.

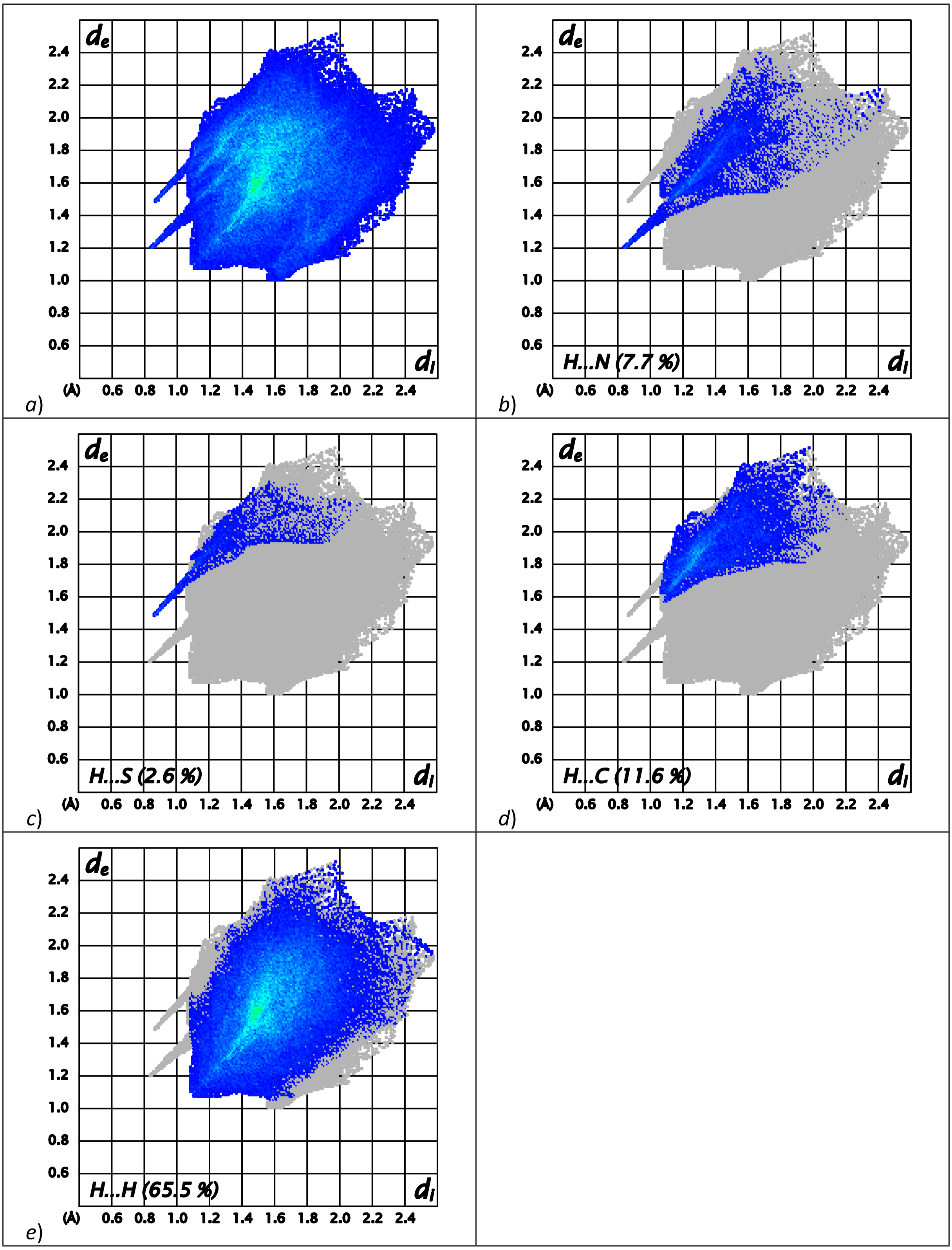

Figure S 14 The full two-dimensional fingerprint plots of **C2**, showing (*a*) all, (*b*) $H\cdots N$, (*c*) $H\cdots S$, (*d*) $H\cdots C$ and (*e*) $H\cdots H$ interactions.

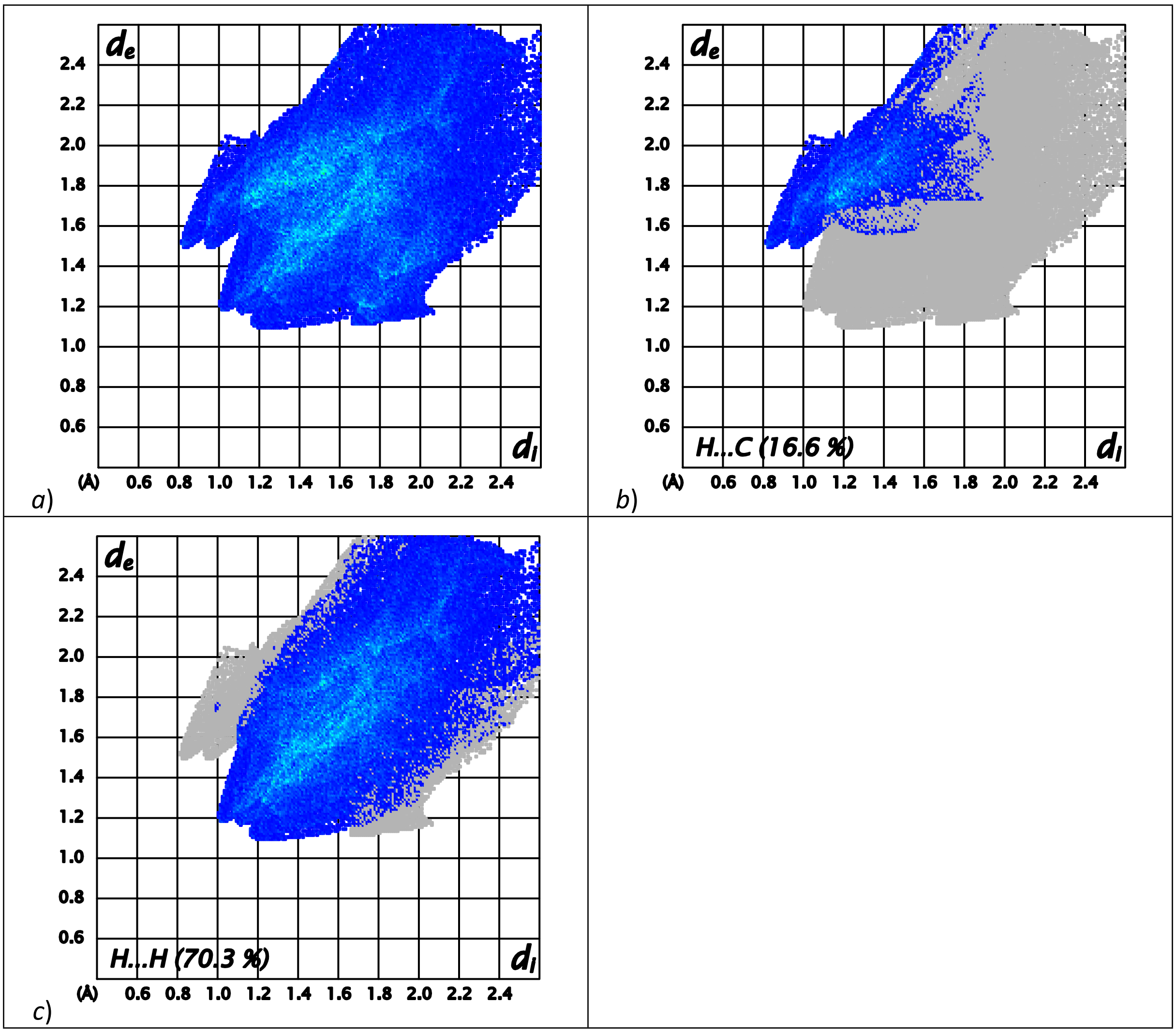

Figure S 15 The full two-dimensional fingerprint plots of **C3**, showing (*a*) all, (*b*) H···C and (*c*) H···H interactions.

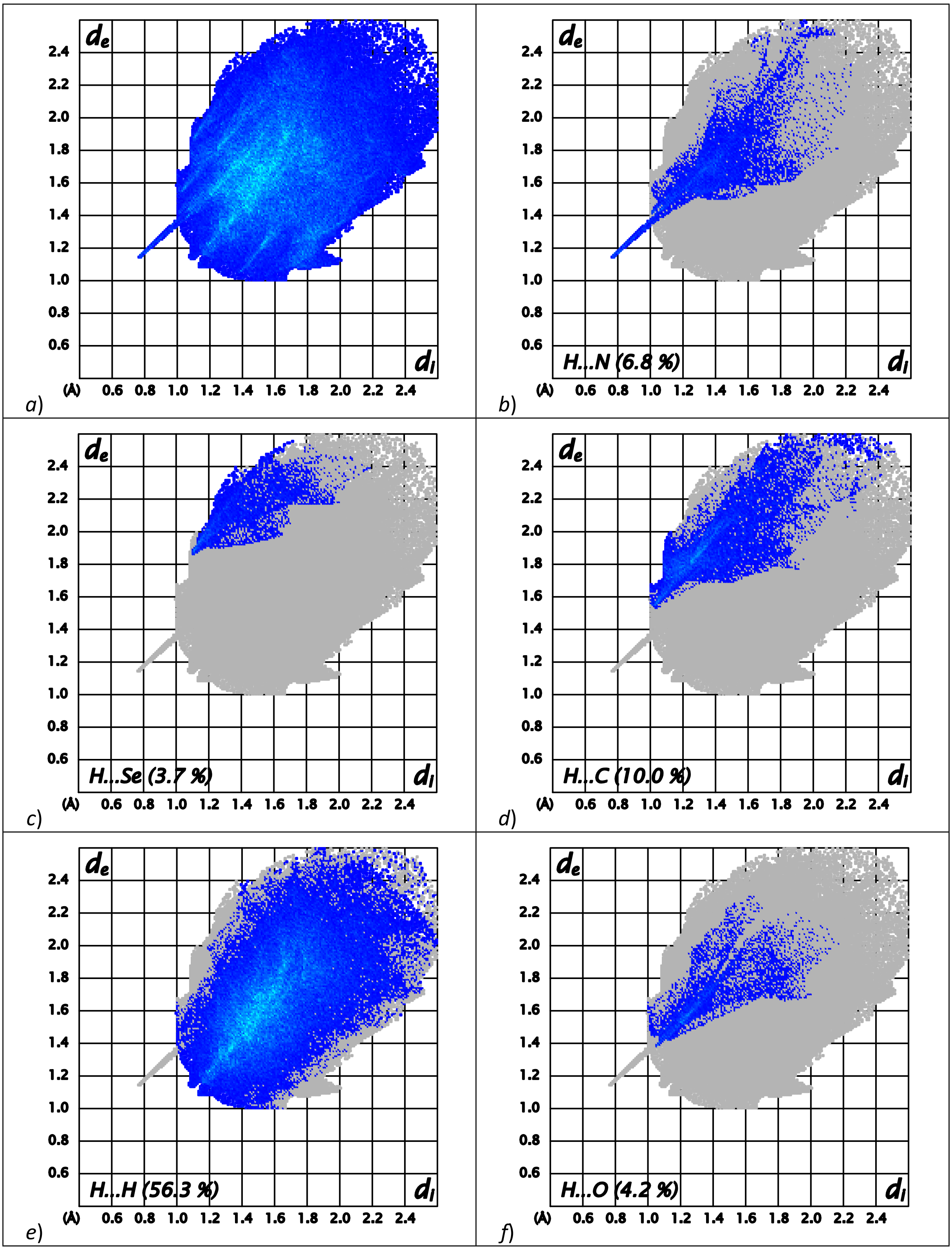

Figure S 16 The full two-dimensional fingerprint plots of **C4**, showing (*a*) all, (*b*) H···N, (*c*) H···Se, (*d*) H···C, (*e*) and (*f*) H···H interactions.

S7 QT-AIM calculations

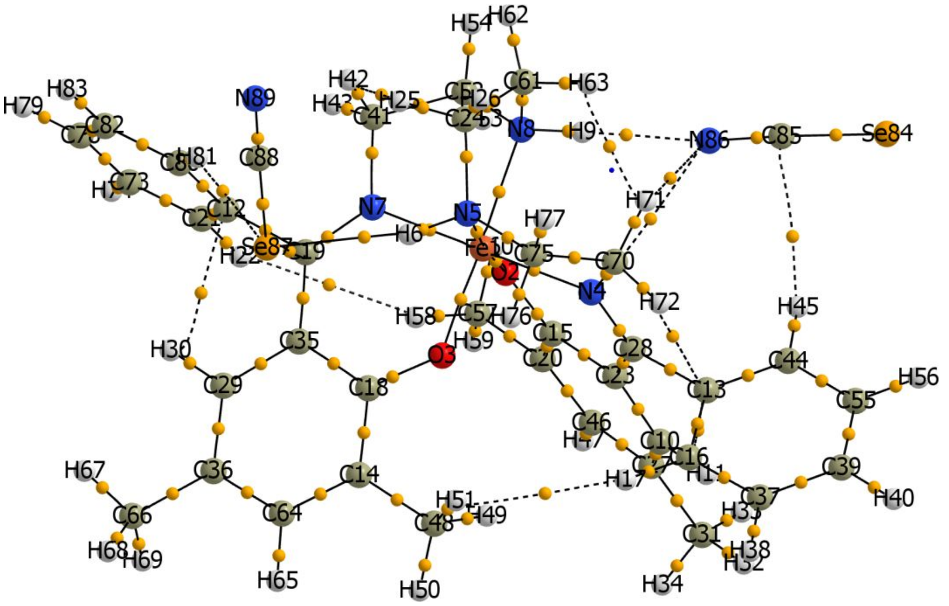

Figure S 17 The molecular graph for **C1** with labelled atoms and depicted (3,-1) bond critical points (yellow spheres)

Table S7 Topological and energetic properties of ρ(r) calculated for **C1** at the selected (3,-1) critical points.

**Atoms**	ρ**(r)**	$\nabla^2\rho$**(r)**	ε	***G*(r)**	H_e**(r)**	***V*(r)**	**|*V*(r)|/*G*(r)**	E_{int}**/kcal.mol**
Fe1 - N4	0.096299	+0.471221	0.199614	0.1367	-0.0189	-0.1555	1.14	-48.80
Fe1 - N5	0.093885	+0.400159	0.517895	0.1203	-0.0203	-0.1405	1.17	-44.10
Fe1 - N7	0.094753	+0.478035	0.208081	0.1372	-0.0177	-0.1549	1.13	-48.59
Fe1 - N8	0.088920	+0.389080	0.444301	0.1155	-0.0182	-0.1337	1.16	-41.96
Fe1 - O2	0.119295	+0.699222	0.431272	0.1962	-0.0214	-0.2176	1.11	-68.27
Fe1 - O3	0.108142	+0.622934	0.389542	0.1736	-0.0178	-0.1914	1.10	-60.05
H6 - Se87	0.025242	+0.038028	0.065882	0.0106	-0.0011	-0.0117	1.10	-3.67
H9 - N86	0.024285	+0.069171	0.006410	0.0157	0.0016	-0.0140	0.90	-4.40

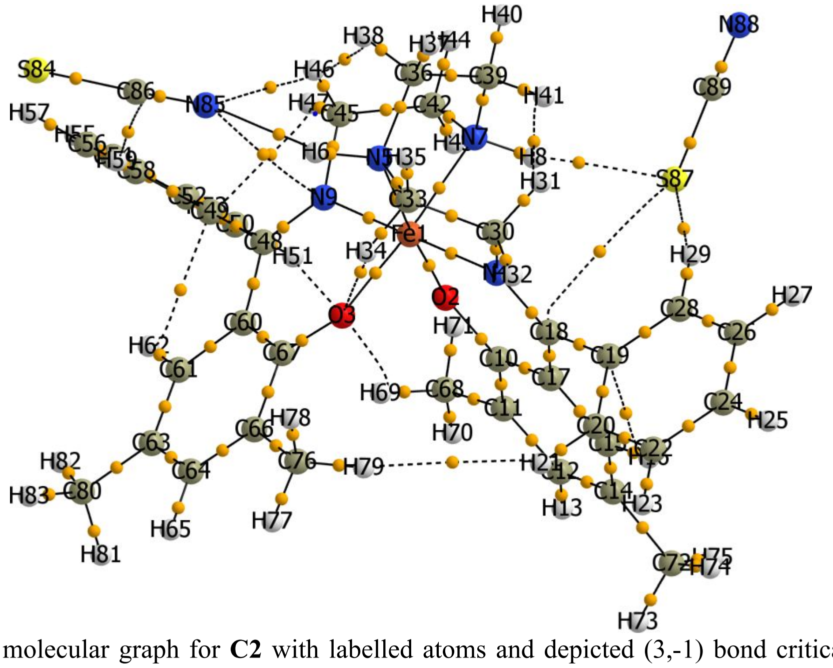

Figure S 18 The molecular graph for **C2** with labelled atoms and depicted (3,-1) bond critical points (yellow spheres)

Table S8 Topological and energetic properties of ρ(r) calculated for **C2** at the selected (3,-1) critical points**.**

**Atoms**	ρ**(r)**	$\nabla^2\rho$**(r)**	ε	***G*(r)**	***H*$_e$(r)**	***V*(r)**	**|*V*(r)|/*G*(r)**	***E*$_{int}$/kcal.mol**
Fe1 - N4	0.098864	+0.505185	0.195188	0.1453	-0.0190	-0.1644	1.13	-51.58
Fe1 - N5	0.089619	+0.384728	0.428012	0.1150	-0.0189	-0.1339	1.16	-42.01
Fe1 - N7	0.090826	+0.380395	0.520203	0.1145	-0.0194	-0.1339	1.17	-42.00
Fe1 - N9	0.101190	+0.502263	0.175096	0.1461	-0.0205	-0.1666	1.14	-52.26
Fe1 - O2	0.112235	+0.652497	0.379503	0.1822	-0.0191	-0.2014	1.10	-63.18
Fe1 - O3	0.111146	+0.628141	0.451895	0.1764	-0.0194	-0.1959	1.11	-61.45
H6 - N85	0.025993	+0.074341	0.005126	0.0169	0.0017	-0.0152	0.90	-4.77
H8 - S87	0.024774	+0.043406	0.059110	0.0115	-0.0006	-0.0121	1.05	-3.79

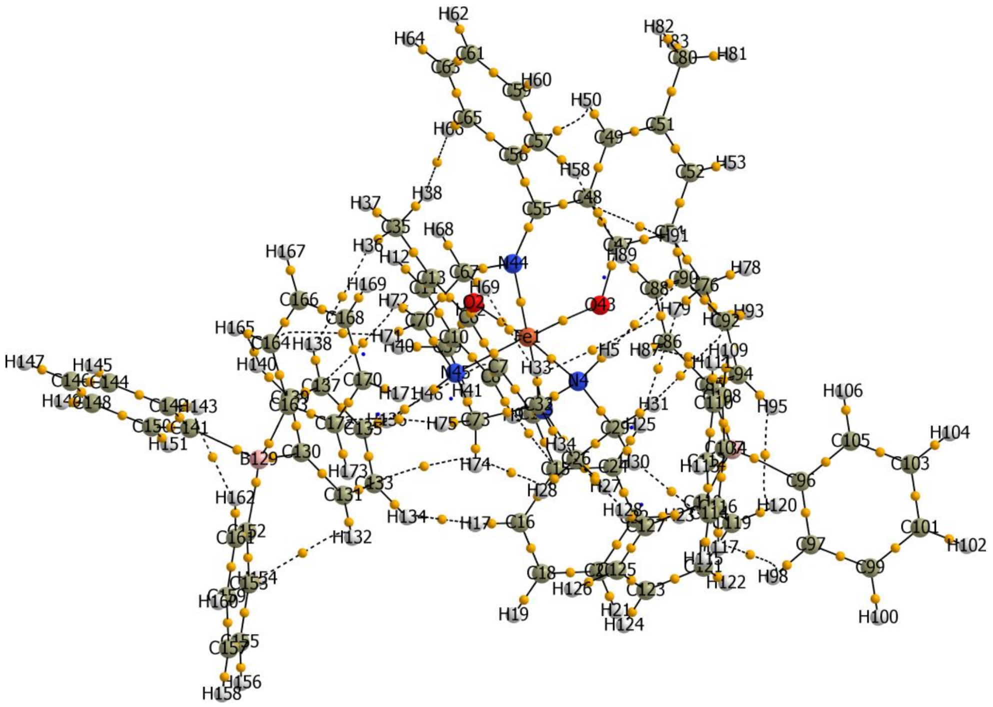

Figure S 19 The molecular graph for **C3** with labelled atoms and depicted (3,-1) bond critical points (yellow spheres)

Table S9 Topological and energetic properties of ρ(r) calculated for **C3** at the selected (3,-1) critical points**.**

**Atoms**	ρ**(r)**	$\nabla^2\rho$**(r)**	ε	***G*(r)**	***H*$_e$(r)**	***V*(r)**	**|*V*(r)|/*G*(r)**	***E*$_{int}$/kcal.mol**
Fe1 - N3	0.097823	+0.507534	0.143221	0.1452	-0.0183	-0.1635	1.13	-51.30
Fe1 - N4	0.090923	+0.375676	0.493291	0.1137	-0.0197	-0.1334	1.17	-41.86
Fe1 - N44	0.097823	+0.507532	0.143221	0.1452	-0.0183	-0.1635	1.13	-51.30
Fe1 - N45	0.090923	+0.375675	0.493295	0.1137	-0.0197	-0.1334	1.17	-41.86
Fe1 - O2	0.111788	+0.629150	0.430287	0.1771	-0.0198	-0.1969	1.11	-61.76
Fe1 - O43	0.111788	+0.629151	0.430292	0.1771	-0.0198	-0.1969	1.11	-61.76
H46-C135	0.014782	+0.037435	0.812643	0.0083	0.0011	-0.0072	0.87	-2.25
H5 - C90	0.014782	+0.037435	0.812799	0.0083	0.0011	-0.0072	0.87	-2.25

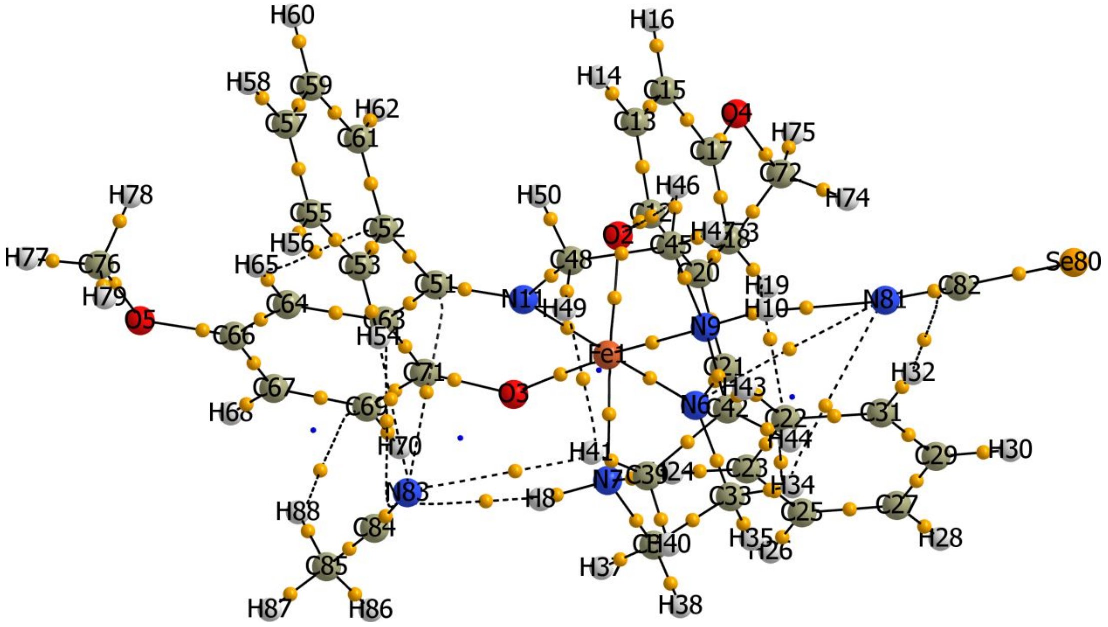

Figure S 20 The molecular graph for **C4** with labelled atoms and depicted (3,-1) bond critical points (yellow spheres)

Table S10 Topological and energetic properties of ρ(r) calculated for **C4** at the selected (3,-1) critical points**.**

Atoms	ρ(r)	$\nabla^2\rho$(r)	ε	G(r)	H_e(r)	V(r)	|V(r)|/G(r)	E_{int}/kcal.mol
Fe1 - N11	0.098042	+0.515727	0.127948	0.1473	-0.0183	-0.1656	1.12	-51.96
Fe1 - N6	0.100788	+0.502189	0.186079	0.1459	-0.0204	-0.1663	1.14	-52.18
Fe1 - N7	0.088855	+0.405369	0.561511	0.1186	-0.0173	-0.1358	1.15	-42.61
Fe1 - N9	0.090114	+0.371328	0.496361	0.1123	-0.0195	-0.1317	1.17	-41.32
Fe1 - O2	0.109757	+0.606383	0.371327	0.1710	-0.0194	-0.1905	1.11	-59.77
Fe1 - O3	0.108706	+0.615758	0.459449	0.1725	-0.0186	-0.1911	1.11	-59.96
H10 - N81	0.033722	+0.096619	0.009095	0.0227	0.0014	-0.0213	0.94	-6.68
H8 - N83	0.009148	+0.028544	0.489758	0.0061	0.0011	-0.0050	0.82	-1.57

S8 Magnetic measurements and computational studies

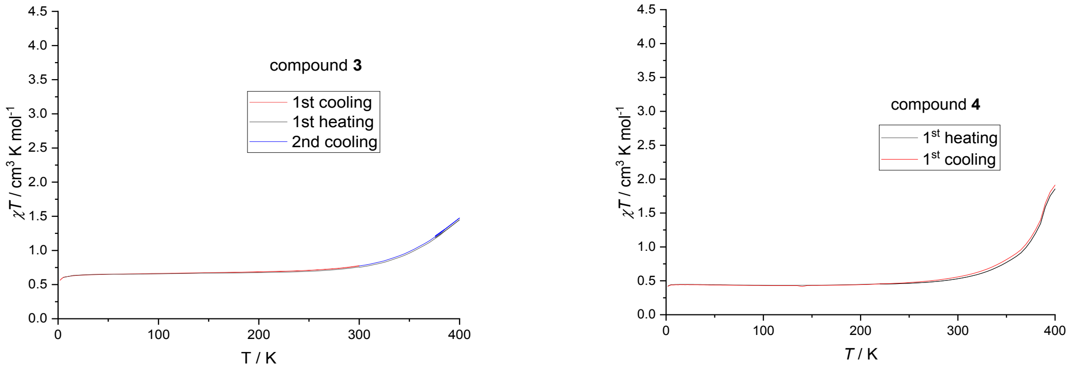

a)

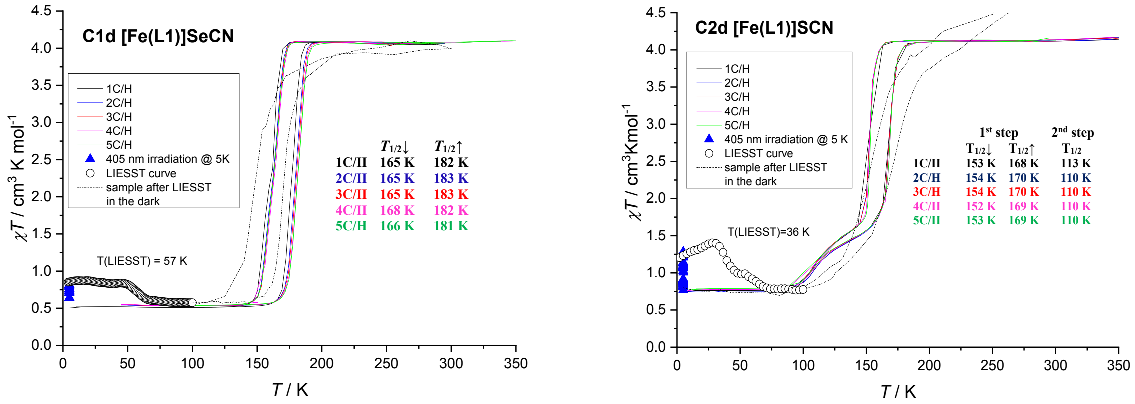

b)

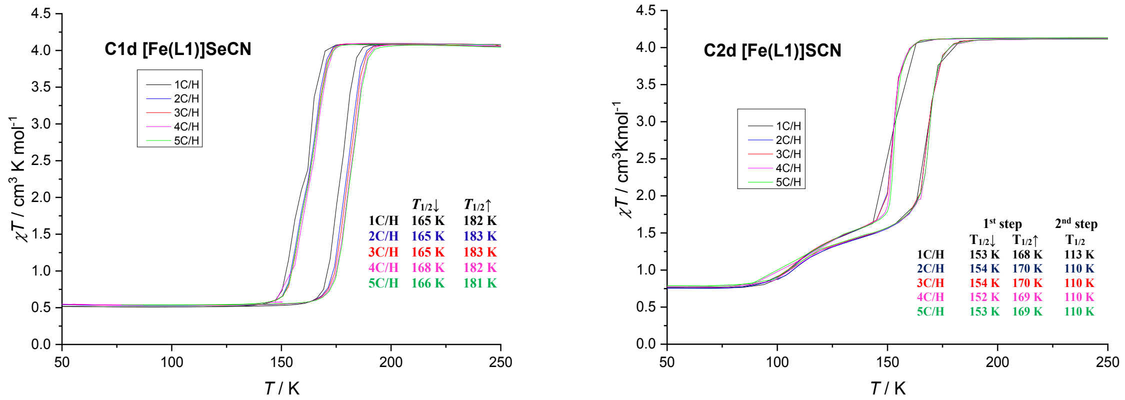

c)

Figure S 21 SCO properties of reported compounds: ***a***) Full temperature-dependent magnetic profiles for **C3** and **C4** recorded in heating and cooling mode. ***b***) Full temperature-dependent magnetic profiles for **C1d** and **C2d** (5-350 K), including the LIESST curve and a reference measurement of sample used for photomagnetic experiments performed in the dark. ***c***) Close-up view of the thermal hysteresis loops for **C1d** and **C2d** over five consecutive cooling/heating cycles.

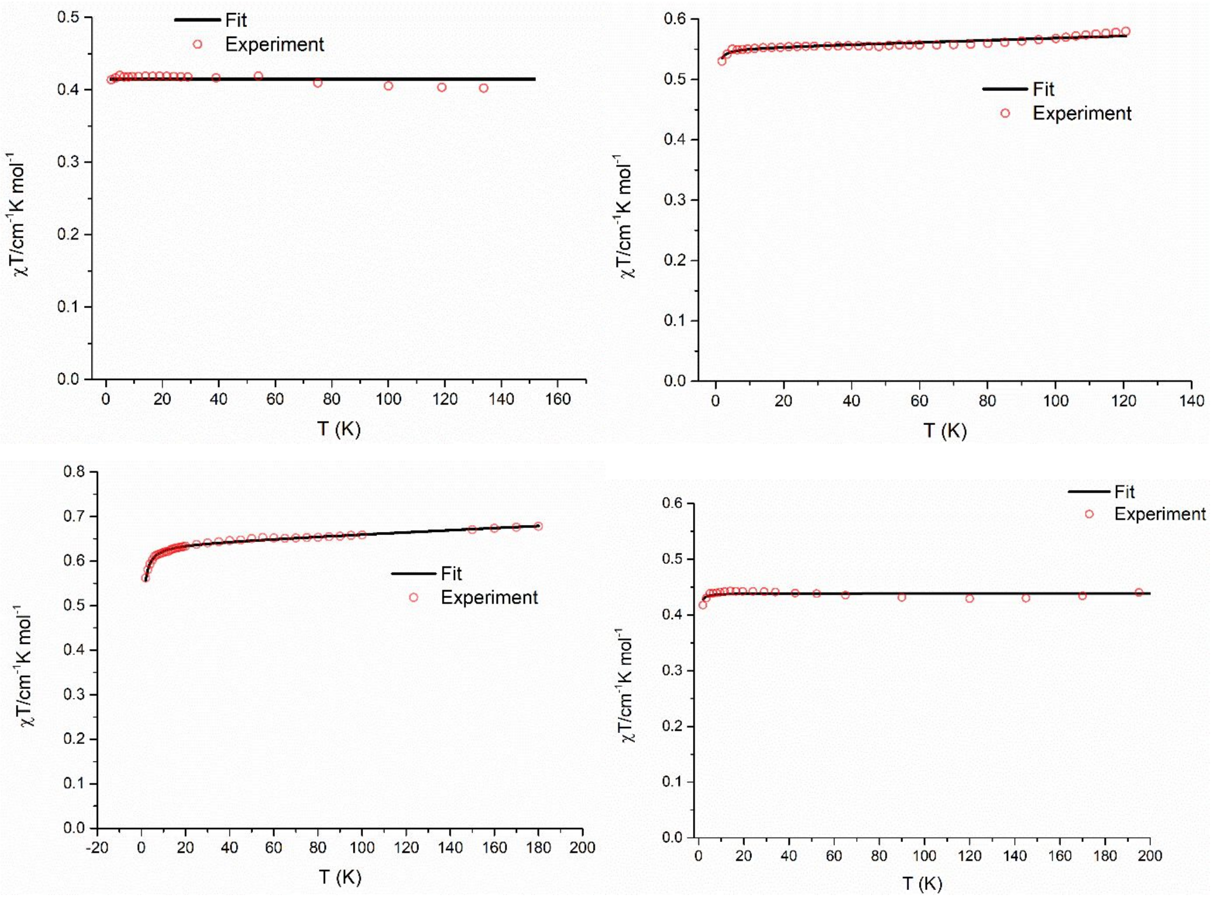

Figure S 22 Comparison of experimental and fitted LS plateaus of χT vs T curves for **C1**-**C4**

Table S 11 Fitted parameters for magnetic susceptibility temperature dependence

Parameter	**C1**	**C2**	**C3**	**C4**
g_{iso}	2.103	2.197	2.184	2.164
χ_{TIP} (cm^3mol^{-1})	0	$0.181\cdot10^{-3}$	$0.246\cdot10^{-3}$	0
x_{IMP}	0	0.025	0.049	0
zJ	0	-0.040	-0.260	-0.029

Table S 12 Calculated ligand field parameters

	B (cm^{-1})	***C*/*B***	10 Dq (cm^{-1})	10 Dq/***B***
**C1**	1040.8	3.363	20659.8	19.8
**C1d**	963.9	3.813	11605.2	12.0
**C2**	1040.6	3.371	20772.3	20.0
**C3**	1040.3	3.347	20398.1	19.6
**C4**	1042.8	3.354	20407.6	19.6

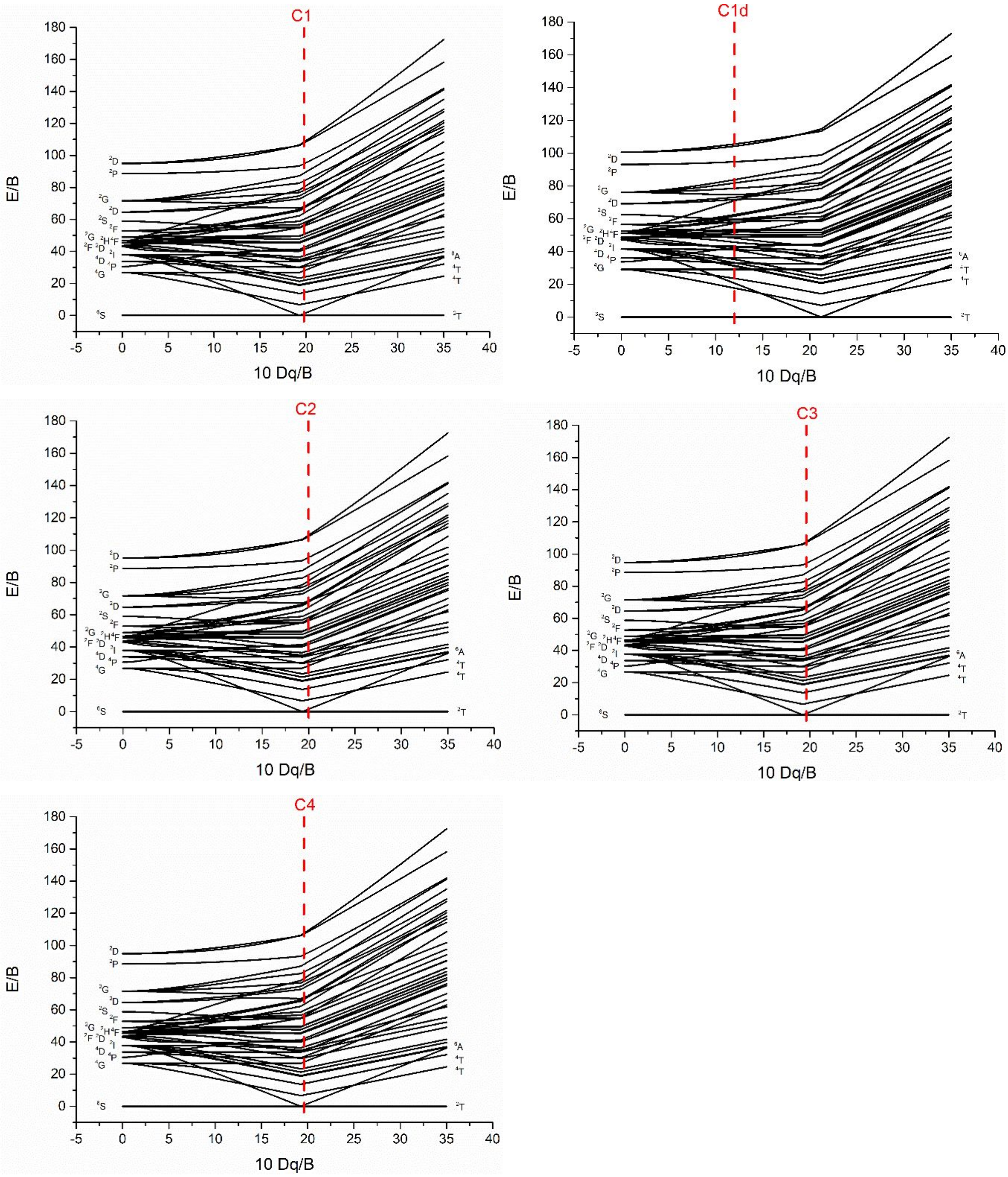

Figure S 23 Tanabe-Sugano diagrams based on calculations on X-ray structures

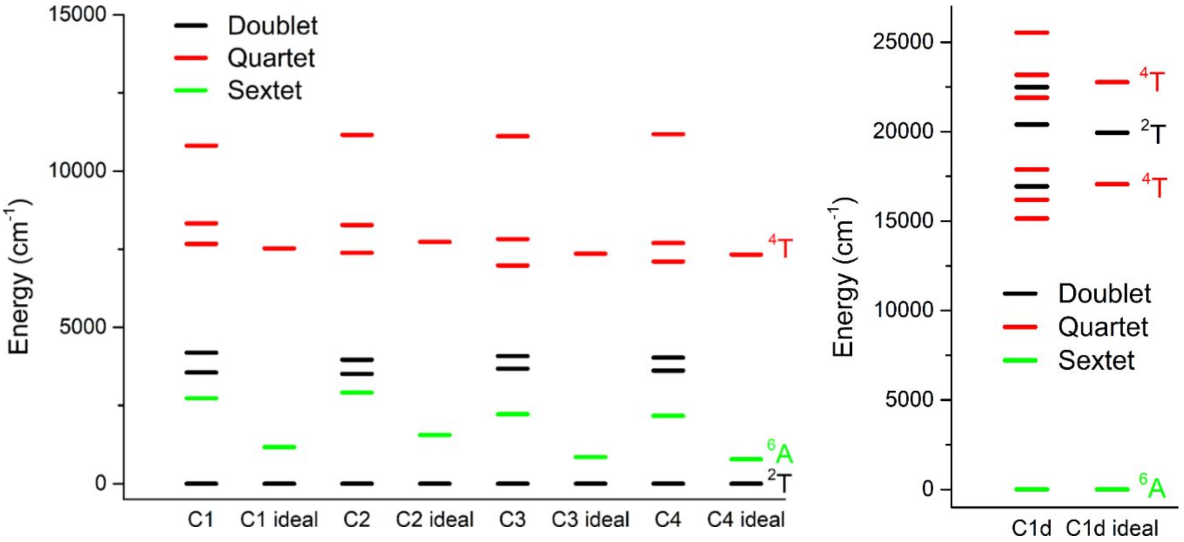

Figure S 24 Comparison of calculated LF terms with ideal octahedron LF terms predicted by Tanabe-Sugano diagram

Table S 13 Selected bond lengths for HS and LS optimized structures (for **C3** LS: O2= O1', N3 = N2', N4 = N1')

Bond lengths	**C1 HS**	**C1 LS**	**C2 HS**	**C2 LS**	**C3 HS**	**C3 LS**	**C4 HS**	**C4 LS**
Fe1-O1	1.9182	1.8652	1.9178	1.8695	1.914	1.8753	1.9213	1.862
Fe1-O2	1.9151	1.8661	1.9213	1.8647	1.9194		1.9204	1.8634
Fe1-N1^{im}	2.171	1.9279	2.1827	1.9296	2.1793	1.9836	2.173	1.9289
Fe1-N2^{am}	2.2285	2.0173	2.211	2.0211	2.2134	2.0464	2.2315	2.0158
Fe1-N3^{am}	2.2123	2.0146	2.2327	2.0155	2.2277		2.2083	2.0168
Fe1-N4^{im}	2.1808	1.928	2.1681	1.9278	2.1717		2.1863	1.9292

Table S 14 Selected angles for HS and LS optimized structures (for **C3** LS: O2 = O1', N3 = N2', N4 = N1')

Bond angles	**C1 HS**	**C1 LS**	**C2HS**	**C2 LS**	**C3HS**	**C3LS**	**C4HS**	**C4LS**
N2-Fe1-N3	98.01	84.1	79.26	84.16	79.12	83.65	79.37	84.12
N2-Fe1-N1	102.85	85.21	78.98	85.25	78.74	84.75	79.24	85.35
N2-Fe1-O2	93.04	90.81	93.2	91.03	93.08	90.03	94.47	90.49
N2-Fe1-N4	161.24	94.11	96.74	94.75	94.93	95.43	91.66	94.5
N4-Fe1-N3	84.86	85.46	79.17	85.47	79.26	84.75	79.02	85.35
O2-Fe1-N4	161.94	92.82	84.31	92.81	84.47	91.75	84.85	93.06
O2-Fe1-N1	94.11	87.5	103.34	87.7	102.45	88.09	97.78	87.03
N1-Fe1-N3	92.41	94.16	92.58	94.03	92.84	95.43	97.12	94.49
N3-Fe1-O1	78.76	90.31	94.04	90.16	94.01	90.03	92.87	90.46
N1-Fe1-O1	79.16	93.08	84.6	92.88	84.93	91.75	84.54	93.19
O1-Fe1-O2	84.55	94.85	97.98	94.73	98.11	96.56	97.62	94.99
O1-Fe1-N4	100.14	87.56	98.49	87.08	100.28	88.09	104.03	86.96

Table S 15 Crystal field parameters for LS and HS optimized structures

	B **(cm^{-1})**	***C/B***	**10 *Dq*** **(cm^{-1})**	**10 *Dq/B***
**C1 LS**	1040.5	3.372	20440.4	19.64
**C1 HS**	964.3	3.867	11202.4	11.61
**C2 LS**	1040.5	3.375	20385.9	19.59
**C2 HS**	966.8	3.849	11273.4	11.66
**C3 LS**	1044.8	3.317	20335.4	19.46
**C3 HS**	962.5	3.859	1170.3	11.60
**C4 LS**	1045.1	3.332	20503	19.62
**C4 HS**	965.8	3.859	11230.3	11.63

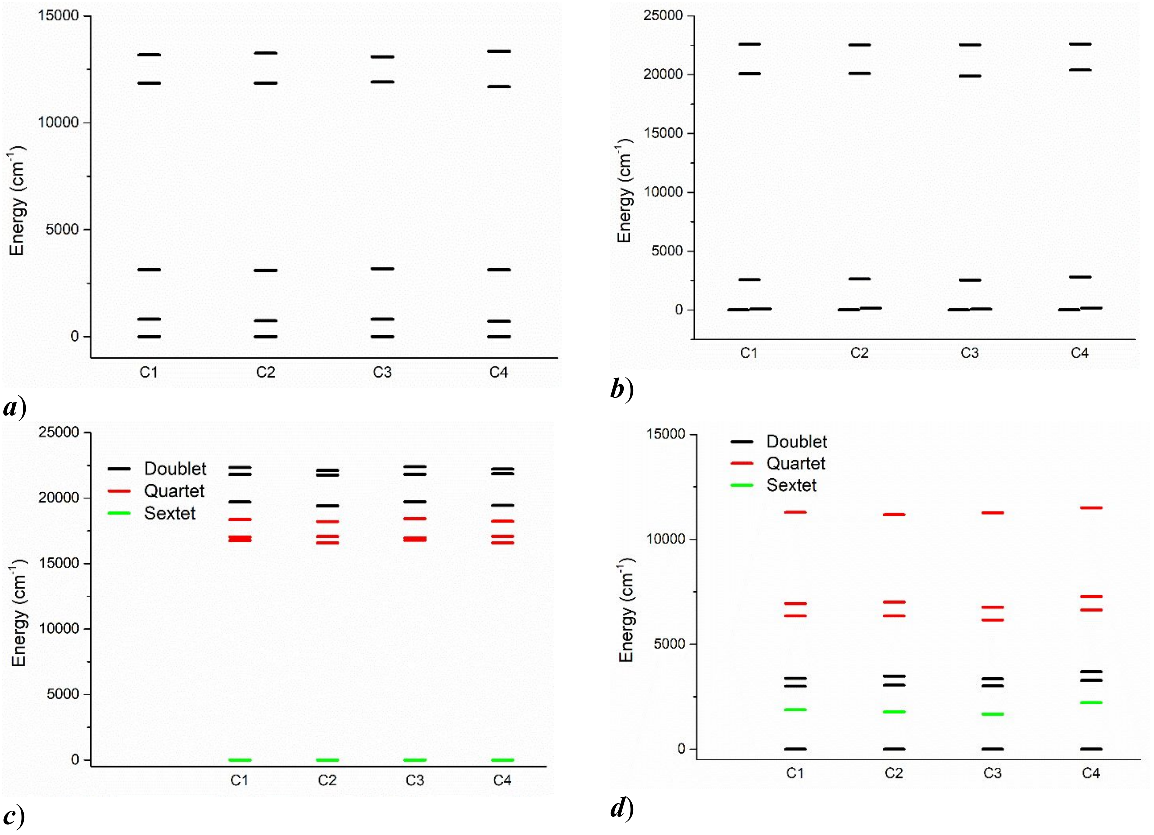

Figure S 25 AILFT calculated *d*-orbitals for HS (***a***) and LS (***b***) structures. NEVPT2-CASSCF LF terms for HS (***c***) and LS (***d***) optimized structures

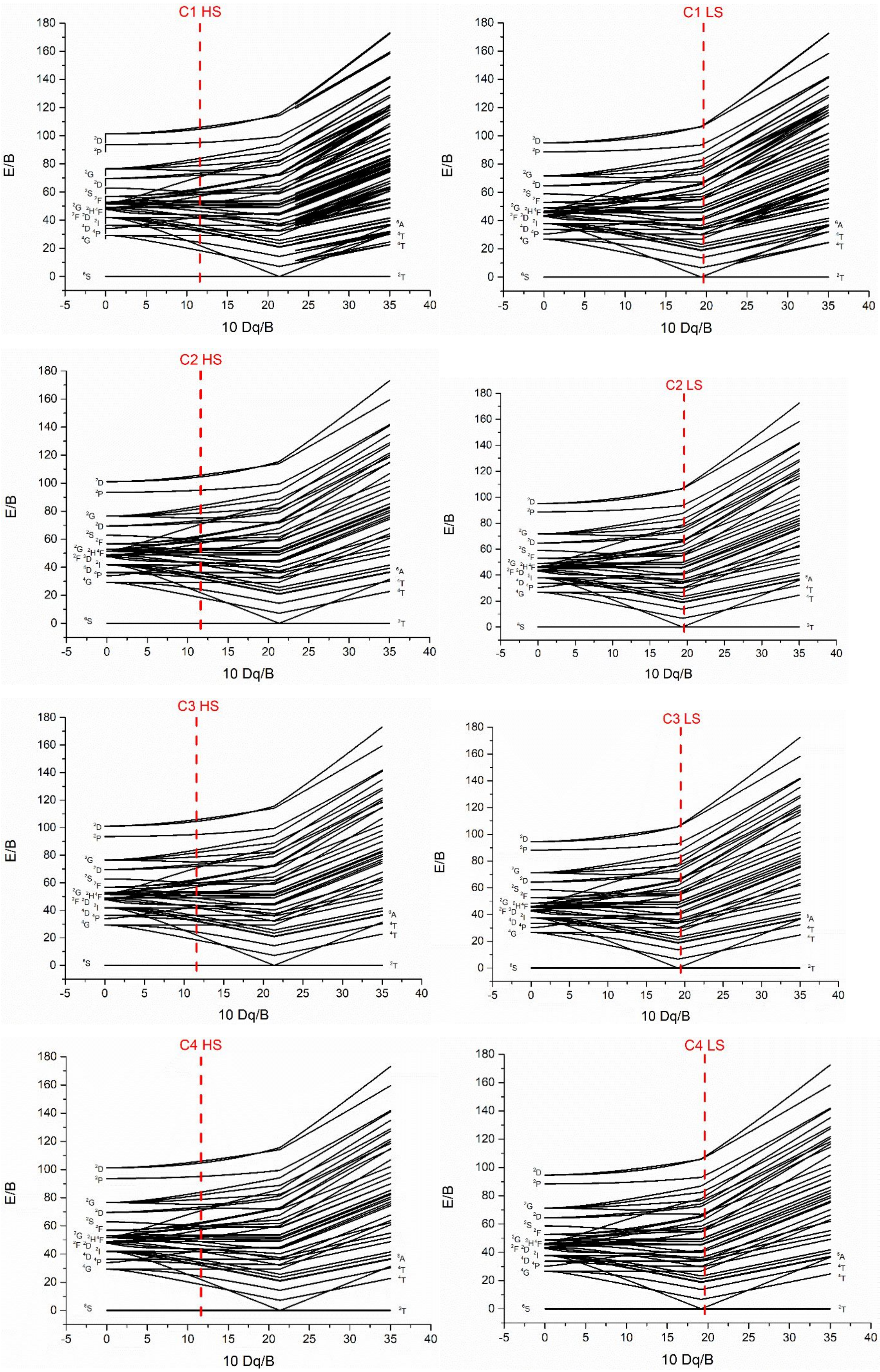

Figure S 26 Calculated Tanabe-Sugano diagrams for HS and LS optimized structures

S9 X-ray powder diffraction studies

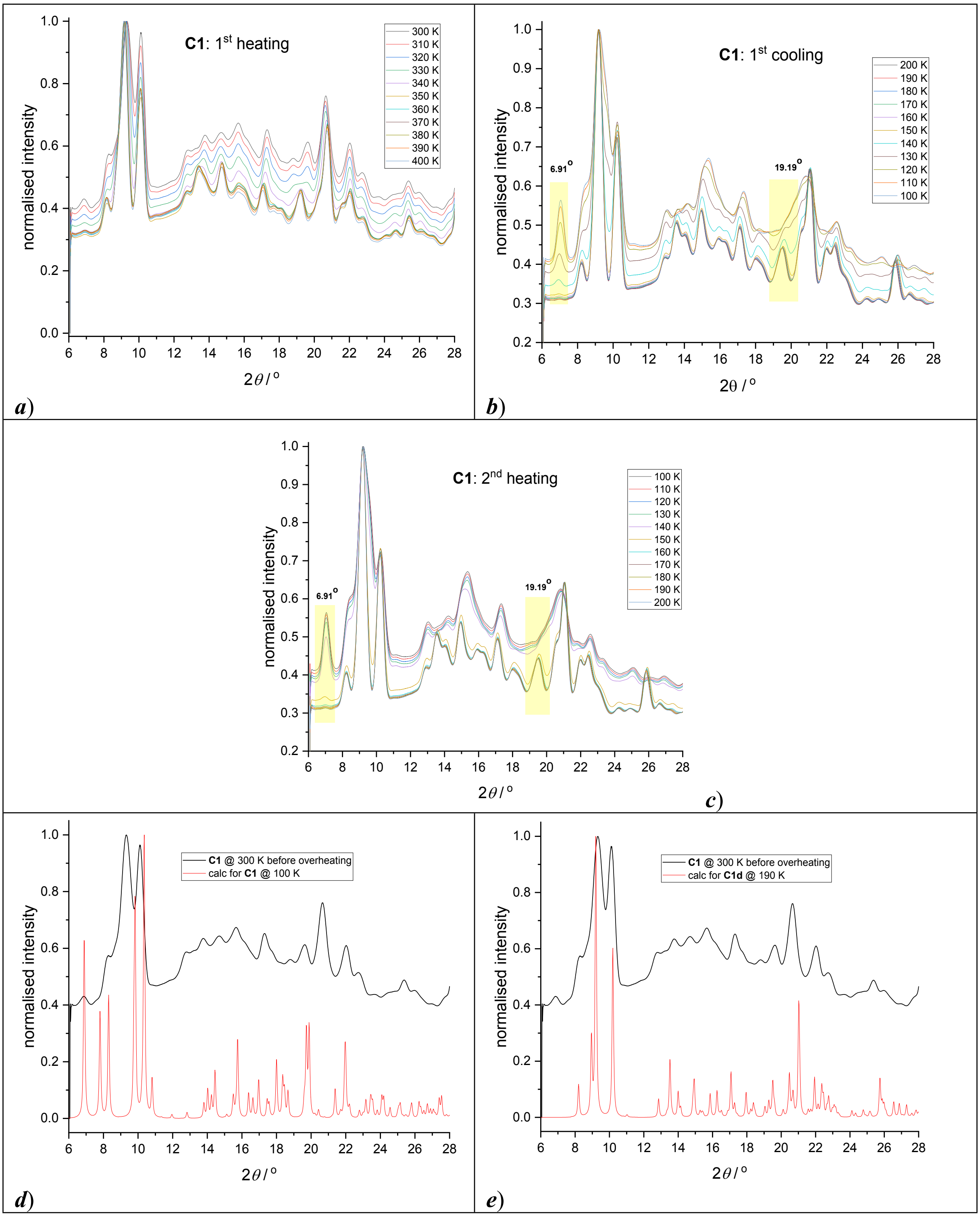

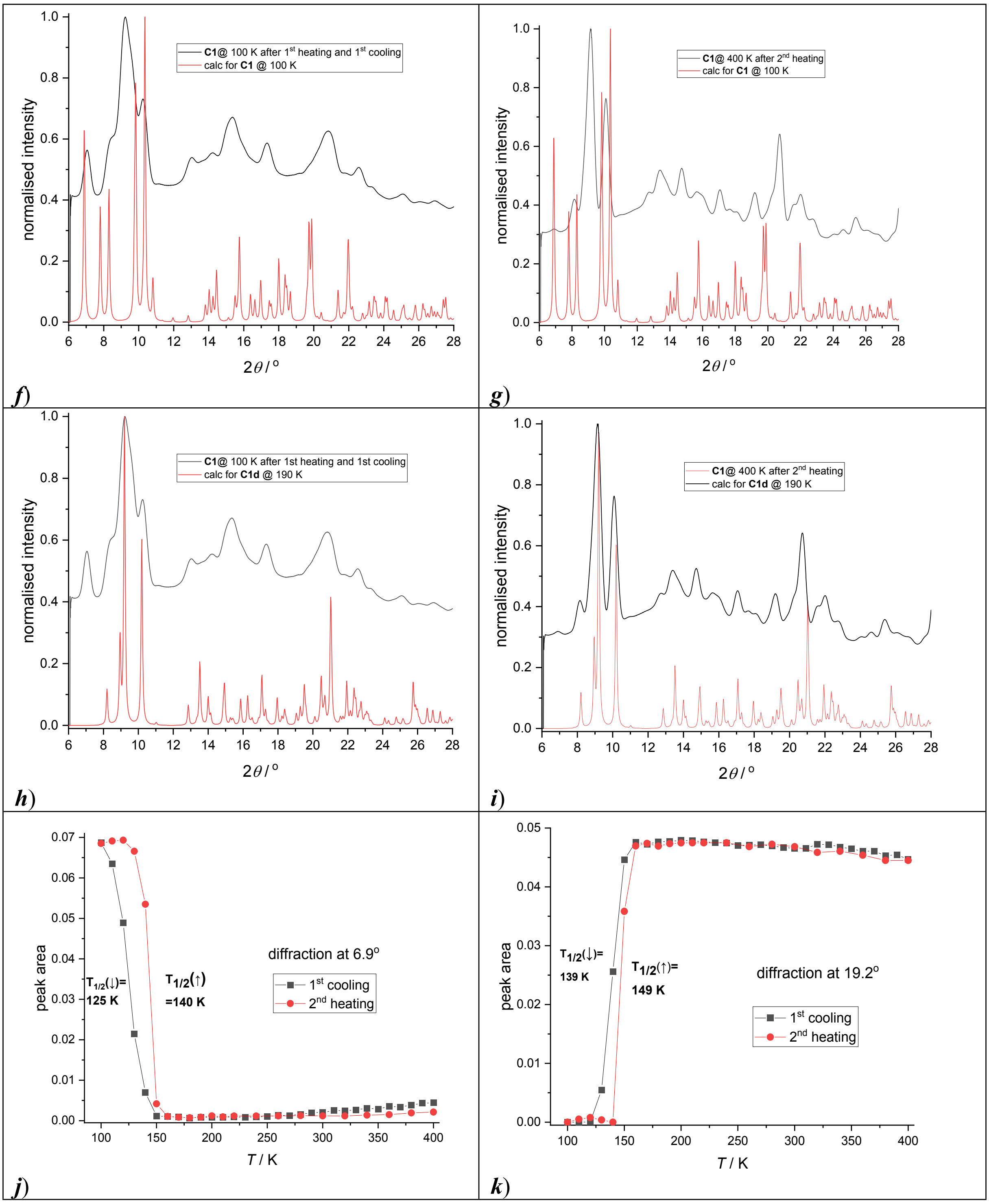

Figure S 27 Temperature-dependent PXPD studies for compound **C1**, recorded within the first heating (***a***), first cooling (***b***) and second heating (***c***). For clarity, diffractograms in the 200–400 K range during the first cooling and second heating are omitted. Comparison of the initial diffractogram from temperature-dependent experiment with the calculated PXPD patterns for solvated structures **C1** (***d***) and **C1d** (***e***). Comparison of diffractograms for the desolvated sample **C1** at 100 K (after first heating and cooling) and at 400 K (after second heating) with the calculated pattern for solvated structure **C1**. Comparison of the **C1** diffractograms at 100 K (after first heating and cooling) and at 400 K (after second heating) with the calculated pattern for structure **C1d**.Temperature-dependent evolution of peak areas at 6.91° (***j***) and 19.19° (***k***).

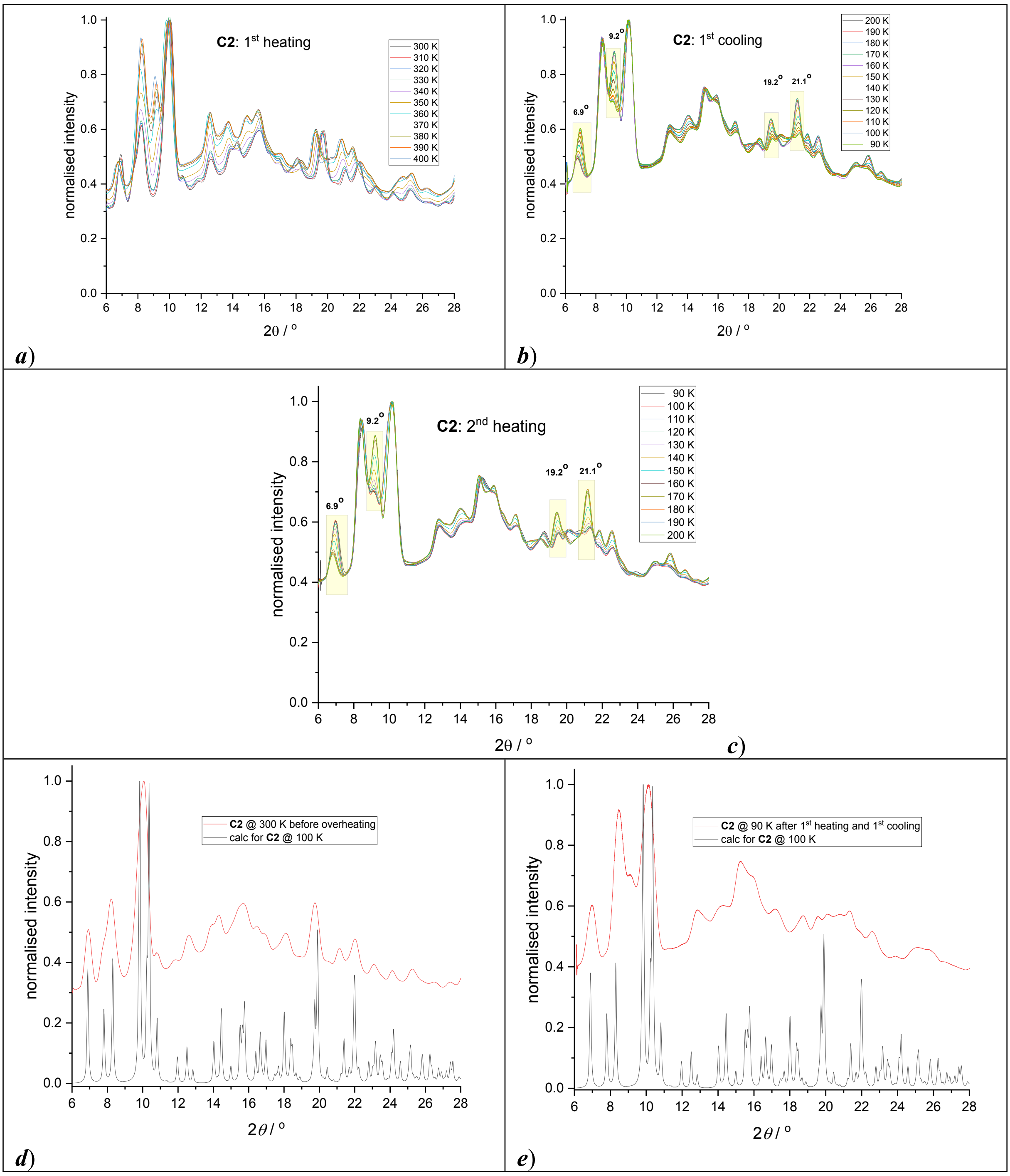
C2: 1st heating
300 K
310 K
320 K
330 K
340 K
350 K
360 K
370 K
380 K
390 K
400 K
normalised intensity
2θ / °
a)
C2: 1st cooling
200 K
190 K
180 K
170 K
160 K
150 K
140 K
130 K
120 K
110 K
100 K
90 K
6.9°
9.2°
19.2°
21.1°
b)
C2: 2nd heating
c)
C2 @ 300 K before overheating
calc for C2 @ 100 K
d)
C2 @ 90 K after 1st heating and 1st cooling
e)

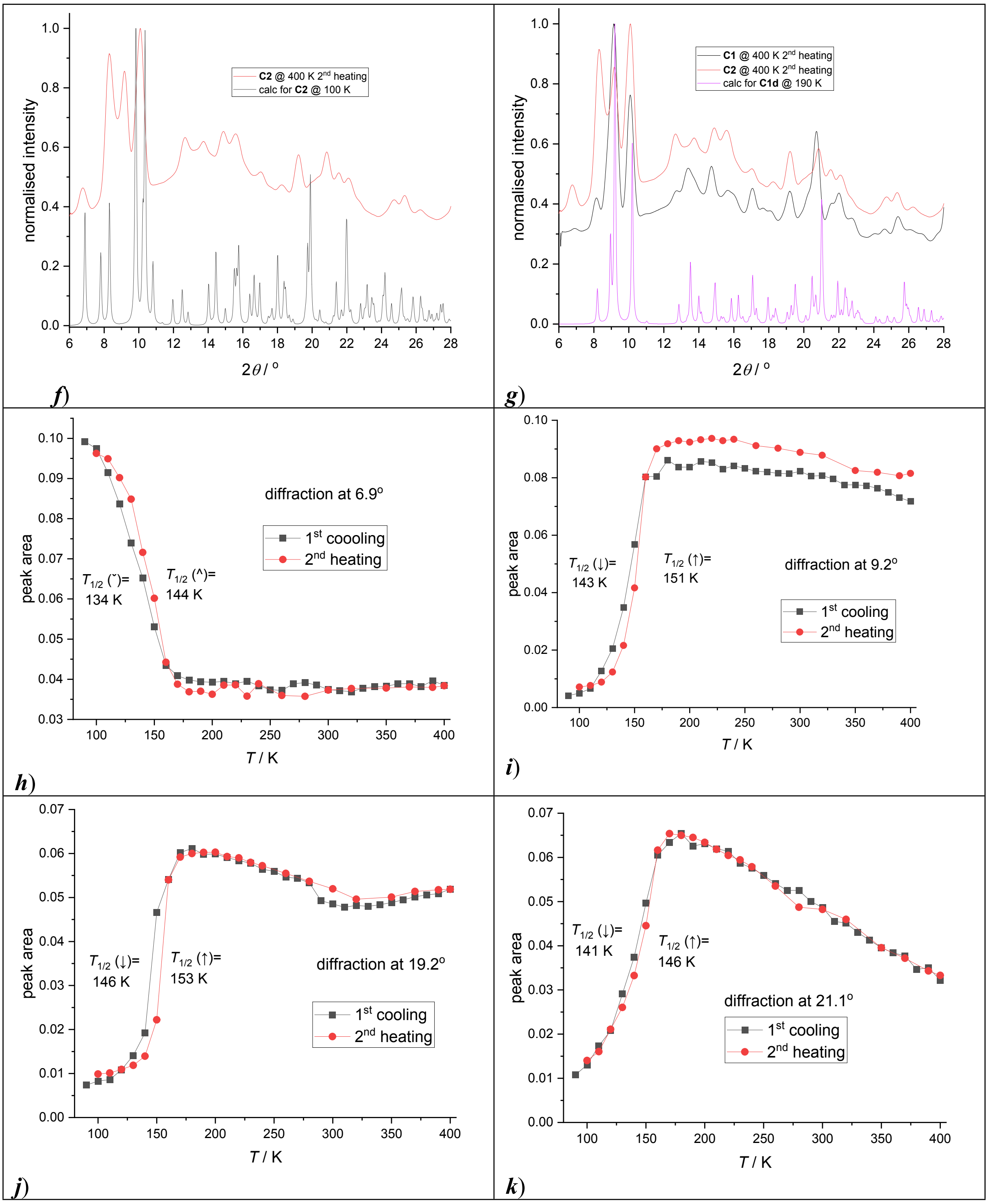

Figure S 28 Temperature-dependent PXPD studies for compound **C2**, recorded within the first heating (***a***), first cooling (***b***) and second heating (***c***). For clarity, diffractograms in the 200–400 K range during the first cooling and second heating are omitted. Comparison of the initial diffractogram from temperature-dependent experiment with the calculated PXPD patterns for solvated structures **C2** (***d***). Comparison of diffractograms for the desolvated sample **C2** at 90 K after first heating and cooling (***e***) and at 400 K after second heating (***f***) with the calculated pattern for solvated structure **C2**. Comparison of diffractograms for the desolvated samples **C1**, **C2** both at 400 K after second heating, with the calculated pattern for structure **C1d**. Temperature dependent evolution of area of peaks at 6.9° (***h***), 9.2° (***i***), 19.2° (***j***) and 21.1°(***k***).

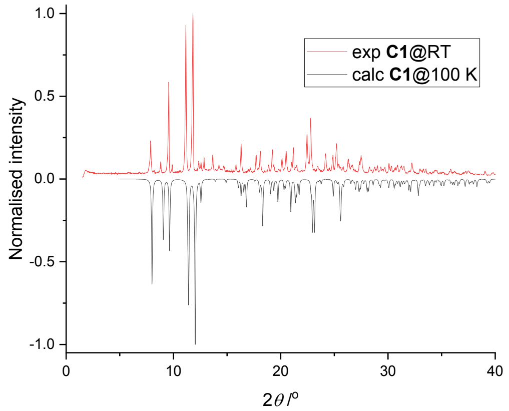
exp C1@RT
calc C1@100 K
Normalised intensity
2θ/°

a)

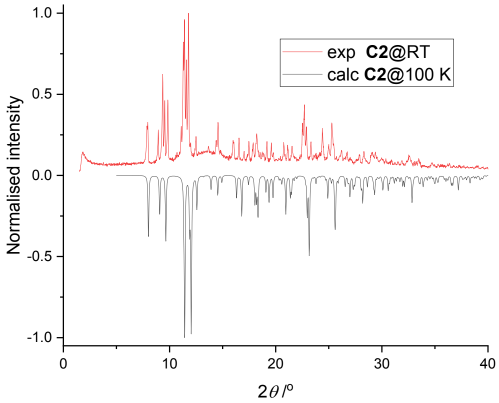
exp C2@RT
calc C2@100 K
Normalised intensity
2θ/°

b)

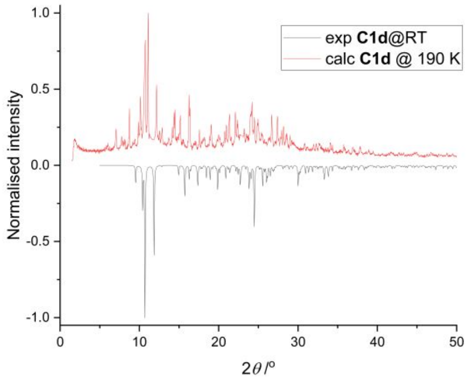
exp C1d@RT
calc C1d @ 190 K
Normalised intensity
2θ/°

c)

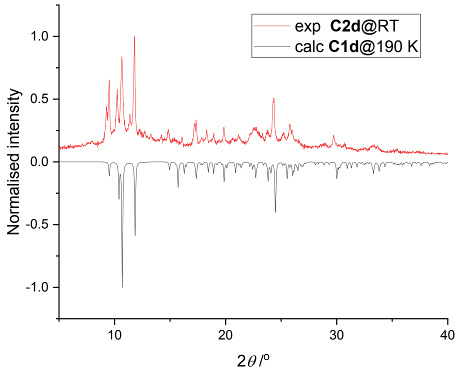
exp C2d@RT
calc C1d@190 K
Normalised intensity
2θ/°

d)

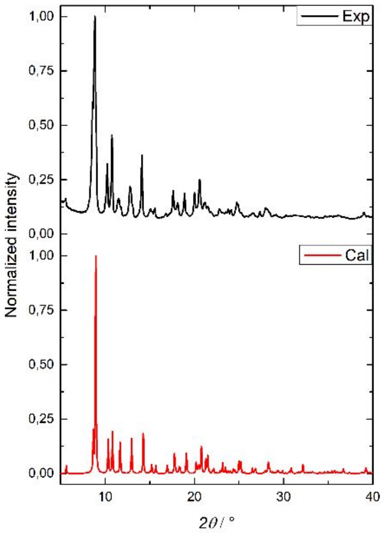
Exp
Cal
Normalized intensity
2θ / °

e)

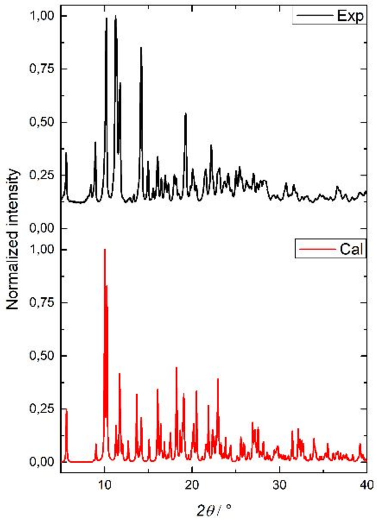
Exp
Cal
Normalized intensity
2θ / °

f)

g)

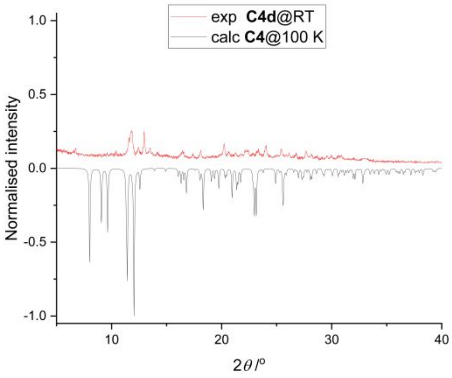
exp C4d@RT
calc C4@100 K
Normalised intensity
2θ/°

Figure S 29 PXRD analysis for compounds reported compounds

S10 EPR spectroscopy

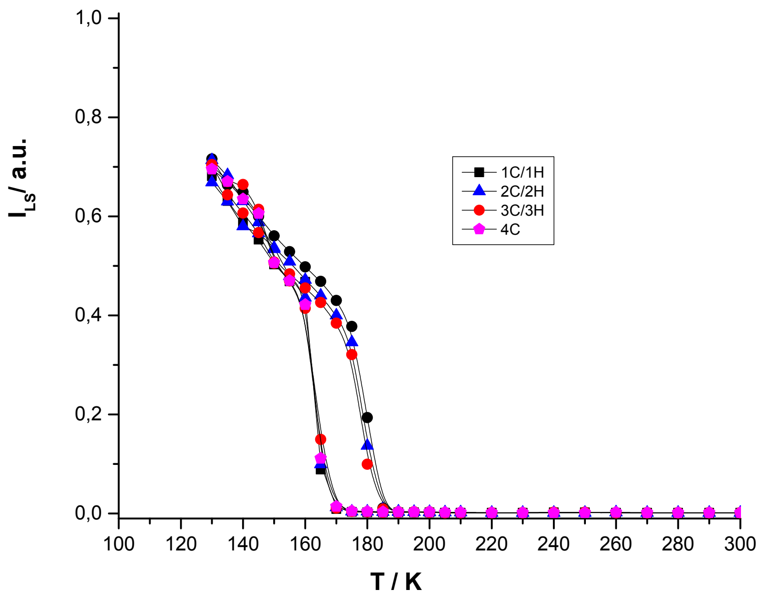

Figure S 30 SCO in **C2d**: Temperature dependence (three subsequent cooling/heating cycles) of relative integral intensity (I_{LS}) of LS (S = 1/2) state resonance signal in the EPR spectra of iron(III) complex **C2d**.

S11 References

[1] Dorbes, S.; Valade, L.; Real, J. A.; Christophe Faulmann. [Fe(Sal2-Trien)][Ni(Dmit)2]: Towards Switchable Spin Crossover Molecular Conductors. *Chemical Communications* **2004**, 1, 69–71. https://doi.org/10.1039/b412182a.

[2] Martinho, P. N.; Harding, C. J.; Helge Müller-Bunz; Albrecht, M.; Morgan, G. G. Inducing Spin Crossover in Amphiphilic Iron(III) Complexes. *European Journal of Inorganic Chemistry* **2010**, 5, 675–679. https://doi.org/10.1002/ejic.200901183.

[3] a) Pritchard, R.; Barrett, S. A.; Kilner, C. A.; Halcrow, M. A. The Influence of Ligand Conformation on the Thermal Spin Transitions in Iron(III) Saltrien Complexes. *Dalton Transactions* **2008**, 24, 3159–3168. https://doi.org/10.1039/b801892h; b) Halcrow, M. A. Structure:function Relationships in Molecular Spin-Crossover Complexes. *Chemical Society Reviews* **2011**, *40* (7), 4119. https://doi.org/10.1039/c1cs15046d.

[4] Shvachko, Y. N.; Starichenko, D. V.; Korolyov, A. V.; Kotov, A. I.; Buravov, L. I.; Zverev, V. N.; Simonov, S. V.; Zorina, L. V.; Yagubskii, E. B. The Highly Conducting Spin-Crossover Compound Combining Fe(III) Cation Complex with TCNQ in a Fractional Reduction State. Synthesis, Structure, Electric and Magnetic Properties. *Magnetochemistry* **2017**, *3* (1), 9. https://doi.org/10.3390/magnetochemistry3010009..

[5] Martinho, P. N.; Harding, C. J.; Helge Müller-Bunz; Albrecht, M.; Morgan, G. G. Inducing Spin Crossover in Amphiphilic Iron(III) Complexes. *European Journal of Inorganic Chemistry* **2010**, 5, 675–679. https://doi.org/10.1002/ejic.200901183.

[6] Martinho, P. N.; Lemma, T.; Gildea, B.; Picardi, G.; Müller-Bunz, H.; Forster, R. J.; Keyes, T. E.; Redmond, G.; Morgan, G. G. Template Assembly of Spin Crossover One-Dimensional Nanowires. *Angewandte Chemie International Edition* **2012**, *51* (48), 11995–11999. https://doi.org/10.1002/anie.201205122.

[7] Vieira, B. J. C.; da Gama, V.; Santos, I. C.; Pereira, L. C. J.; Bandeira, N. A. G.; Waerenborgh, J. C. Magnetic and Structural Correlations in [Fe(Nsal$_2$Trien)] Salts: The Role of Cation–Anion Interactions in the Spin Crossover Phenomenon. *CrystEngComm* **2018**, *20* (17), 2465–2475. https://doi.org/10.1039/c8ce00275d.

[8] Vieira, B. J. C.; Coutinho, J. T.; Santos, I. C.; Pereira, L. C. J.; Waerenborgh, J. C.; da Gama, V. [Fe(Nsal$_2$Trien)]SCN, a New Two-Step Iron(III) Spin Crossover Compound, with Symmetry Breaking Spin-State Transition and an Intermediate Ordered State. *Inorganic Chemistry* **2013**, *52* (7), 3845–3850. https://doi.org/10.1021/ic302533b.

[9] Yu, Z.-M.; Zhao, S.-Z.; Wang, Y.-T.; Xu, P.-Y.; Qin, C.-Y.; Li, Y.-H.; Zhou, X.-H.; Wang, S. Anion-Driven Supramolecular Modulation of Spin-Crossover Properties in Mononuclear Iron(III) Schiff-Base Complexes. *Dalton Transactions* **2021**, *50* (42), 15210–15223. https://doi.org/10.1039/d1dt02394b.

[10] Chen, L.-W.; Zhu, H.; Zhou, H.-W.; Zhang, H.-Z.; Zhao, S.-Z.; Li, Y.-H.; Wang, S. Halogen-Driven Spin Dynamics: Exploring Correlation of Cooperativity and Spin Crossover in Solid-State Mononuclear Fe(III) Schiff-Base Complexes. *Inorganic Chemistry* **2025**, 7203−7213. https://doi.org/10.1021/acs.inorgchem.5c00885.

[11] Floquet, S.; Muñoz, M. C.; Rivière, E.; Clément, R.; Audière; J.-P.; Boillot, M.-L. Structural Effects on the Magnetic Properties of Ferric Complexes in Molecular Materials or a Lamellar CdPS$_3$Host Matrix. *New Journal of Chemistry* **2004**, *28* (4), 535–541. https://doi.org/10.1039/b311371j.

[12] Nemec, I.; Herchel, R.; Šalitroš, I.; Trávníček, Z.; Moncoľ, J.; Fuess, H.; Ruben, M.; Linert, W. Anion Driven Modulation of Magnetic Intermolecular Interactions and Spin Crossover Properties in an Isomorphous Series of Mononuclear Iron(III) Complexes with a Hexadentate Schiff Base Ligand. *CrystEngComm* **2012**, *14* (20), 7015–7024. https://doi.org/10.1039/c2ce25862e.

[13] Nemec, I.; Zoufalý, P.; Jewula, P.; Antal, P.; Linert, W.; Herchel, R. Ion-Pair Complexes of Schiff Base Fe(III) Cations and Complex Anions. *New Journal of Chemistry* **2019**, *43* (12), 4937–4946. https://doi.org/10.1039/c9nj00192a.

[14] Kagesawa, K.; Ichikawa, Y.; Iguchi, H.; Breedlove, B. K.; Li, Z.; Yamashita, M.; Okazawa, A.; Kosaka, W.; Miyasaka, H. Water-Vapor Sensitive Spin-State Switching in an Iron(III) Complex with Nucleobase Pendants Making Flexible Hydrogen-Bonded Networks. *Chemistry Letters* **2019**, *48* (10), 1221–1224. https://doi.org/10.1246/cl.190532.

[15] Griffin, M.; Shakespeare, S.; Shepherd, H. J.; Harding, C. J.; Létard, J.-F.; Desplanches, C.; Goeta, A. E.; Howard, J. A. K.; Powell, A. K.; Mereacre, V.; Garcia, Y.; Naik, A. D.; Müller-Bunz, H.; Morgan, G. G. A Symmetry-Breaking Spin-State Transition in Iron(III). *Angewandte Chemie International Edition* **2010**, *50* (4), 896–900. https://doi.org/10.1002/anie.201005545.

[16] Gandolfi, C.; Moitzi, C.; Schurtenberger, P.; Morgan, G. G.; Albrecht, M. Improved Cooperativity of Spin-Labile Iron(III) Centers by Self-Assembly in Solution. *Journal of the American Chemical Society* **2008**, *130* (44), 14434–14435. https://doi.org/10.1021/ja806611y.

[17] Gandolfi, C.; Moitzi, C.; Schurtenberger, P.; Morgan, G. G.; Albrecht, M. Improved Cooperativity of Spin-Labile Iron(III) Centers by Self-Assembly in Solution. *Journal of the American Chemical Society* **2008**, *130* (44), 14434–14435. https://doi.org/10.1021/ja806611y.

[18] Shvachko, Y. N.; Spitsyna, N. G.; Starichenko, D. V.; Zverev, V. N.; Zorina, L. V.; Simonov, S. V.; Blagov, M. A.; Yagubskii, E. B. Magnetism, Conductivity and Spin-Spin Interactions in Layered Hybrid Structure of Anionic Radicals [Ni(Dmit)2] Alternated by Iron(III) Spin-Crossover Complex [Fe(III)(3-OMe-Sal_2trien)] and Ferric Moiety Precursors. *Molecules* **2020**, *25* (21), 4922. https://doi.org/10.3390/molecules25214922.

[19] Spitsyna, N. G.; Shvachko, Y. N.; Starichenko, D. V.; Lahderanta, E.; Komlev, A. A.; Zorina, L. V.; Simonov, S. V.; Blagov, M. A.; Yagubskii, E. B. Evolution of Spin-Crossover Transition in Hybrid Crystals Involving Cationic Iron Complexes [Fe(III)(3-OMesal_2-Trien)]$^+$ and Anionic Gold Bis(Dithiolene) Complexes $Au(Dmit)_2$ and $Au(Dddt)_2$. *Crystals* **2018**, *8* (10), 382. https://doi.org/10.3390/cryst8100382.

[20]

[21] Spitsyna, N.; Ovanesyan, N.; Blagov, M.; Krapivin, V.; Lobach, A.; Dmitriev, A.; Simonov, S.; Zorina, L.; Pilia, L.; Deplano, P.; Vasiliev, A.; Maximova, O.; Yagubskii, E. Multi-Magnetic Properties of a Novel SCO [Fe(3-OMe-Sal_2Trien)][$Fe(Tdas)_2$]·CH_3CN Salt. *European Journal of Inorganic Chemistry* **2020**, *2020* (48), 4556–4567. https://doi.org/10.1002/ejic.202000873.

[22] Maglic, J.B.; Lavendomme, R. J. MoloVol: an easy-to-use program for analyzing cavities, volumes and surface areas of chemical structures. *J. Appl. Cryst.* **2022**, *55*, 1033-1044. https://doi.org/10.1107/S1600576722004988
